# Mechanistic Insights into the DABCO-Catalyzed Cloke–Wilson Rearrangement: A DFT Perspective

**DOI:** 10.1021/acs.joc.3c02011

**Published:** 2023-10-27

**Authors:** Sebastián Gallardo-Fuentes, Lucas Lodeiro, Ricardo Matute, Israel Fernández

**Affiliations:** † Instituto de Química, Facultad de Ciencias, Pontificia Universidad Católica de Valparaíso, Avenida Universidad 330, Curauma, Valparaíso 2373223, Chile; ‡ Departamento de Química, Facultad de Ciencias, Universidad de Chile, Las Palmeras 3425, Ñuñoa, Santiago 7800003, Chile; § Centro Integrativo de Biología y Química Aplicada (CIBQA), Universidad Bernardo O’Higgins, Santiago 8370854, Chile; ∥ Departamento de Química Orgánica I, Facultad de Ciencias Químicas, Universidad Complutense de Madrid, Madrid 28040, Spain

## Abstract

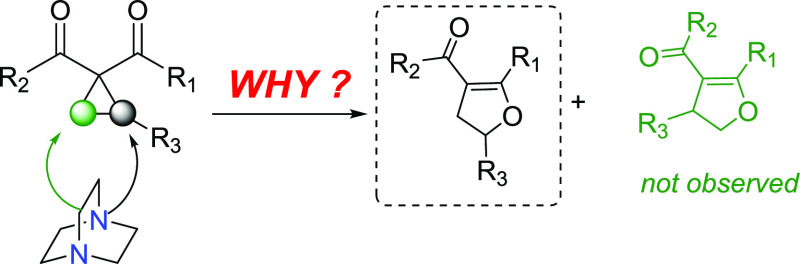

The mechanism and selectivity patterns of the DABCO-catalyzed Cloke–Wilson rearrangement were computationally studied in detail using density functional theory calculations. Our computations suggest that the process occurs stepwise involving the initial ring opening of the cyclopropane promoted by a DABCO molecule followed by a ring-closure reaction of the readily formed zwitterionic intermediate. The regioselectivity of the initial nucleophilic ring-opening step strongly depends on the nature of the substituent attached to the cyclopropane moiety. The physical factors governing the preference for the more sterically hindered C2 (tertiary) position have been quantitatively analyzed by applying the combined activation strain model–energy decomposition analysis method. In addition, our calculations revealed a new mechanism for the analogous transformation involving vinylcyclopropanes consisting of an initial S_N_2′ ring-opening process followed by a *5-exo-trig* cyclization step, which proceeds without facial selectivity.

## Introduction

According to IUPAC, nucleophilic catalysis can be defined as the process by which a Lewis base catalyzes a chemical transformation involving a Lewis adduct as a reaction intermediate.^1^ This Lewis binding interaction elicits a transfer of electron density to the Lewis acidic fragment, enhancing the nucleophilic power of the acceptor moiety.^2^ In terms of the frontier molecular orbital perspective, the activation mode in Lewis base catalysis relies mainly on the HOMO-raising activation concept.^3,4^ Although the activation of unsaturated functionalities such as allenes, alkenes, and alkynes represents the most recognized forms of Lewis base catalysis,^5^ it can also efficiently catalyze the ring-opening reaction of small rings such as epoxides, aziridines, and activated cyclopropanes.^6^

**Scheme 1:**
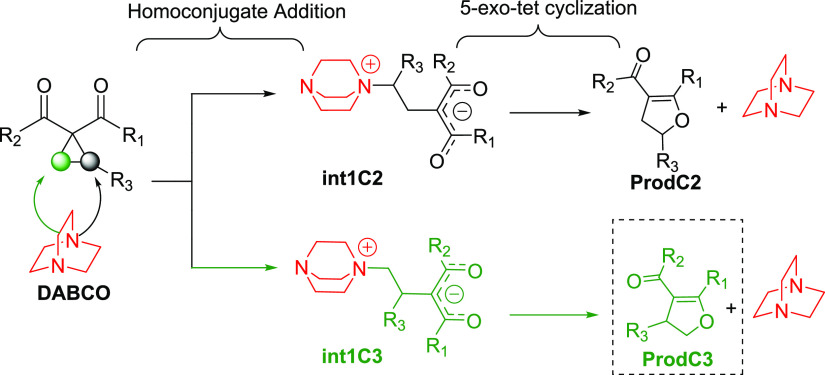
Plausible Reaction Mechanism for the DABCO-Catalyzed Cloke–Wilson Rearrangement of Alkyl- and Aryl-Substituted Cyclopropanes

In this context, the homoconjugate addition of nucleophilic amines to furnish functionalized compounds from activated cyclopropanes constitutes a well-established synthetic methodology.^7,8^ For instance, Liang and co-workers described the synthesis of γ-lactams from donor–acceptor (DA) cyclopropanes catalyzed by 1,4-diazabicyclo[2.2.2]octane (DABCO).^9^ In this process, the DA cyclopropane is activated through a nucleophilic ring-opening reaction to generate a 1,3-zwitterionic intermediate that can engage in a subsequent Michael addition mechanism. Related to this transformation, Xu and co-workers reported the Cloke–Wilson rearrangement of cyclopropyl ketones catalyzed by DABCO to furnish 2,3-dihydrofurans.^10^ The key step in this elegant transformation involves the formation of a zwitterionic intermediate (**int1C2**) through a nucleophilic addition process (Scheme 1). From a mechanistic point of view, the DABCO-catalyzed Cloke–Wilson rearrangement could be described as a tandem reaction consisting of a homoconjugate addition followed by a *5-exo-tet* cyclization leading preferentially to the 2-substituted dihydrofuran **ProdC2**, as depicted in Scheme 1.

Although some mechanistic issues of the DABCO-catalyzed Cloke–Wilson rearrangement of cyclopropyl ketones have been computationally addressed by Wei and co-workers,^11^ the origin of the regio- and stereoselectivity patterns remains not completely understood so far. This prompted us to perform a density functional theory (DFT) study to elucidate the ultimate factors controlling the reactivity and selectivity patterns of the Cloke–Wilson rearrangement. For this purpose, we shall apply state-of-the-art computational methods, namely, the activation strain model (ASM) of chemical reactivity (also called the distortion-interaction model)^12^ in combination with the energy decomposition analysis (EDA) method.^13^ This approach was chosen because it has greatly contributed to our current understanding of fundamental reactions in organic and organometallic chemistry,^12,14^ including catalyzed transformations.^15^

This article is organized as follows: first, we perform a full exploration of the potential energy surface (PES) for the DABCO-catalyzed Cloke–Wilson rearrangement of cyclopropyl ketones containing a phenyl group as a representative process to unambiguously define the regio-determining step along the reaction pathway. Next, we focus on the factors responsible for the experimentally observed regioselectivity patterns for the DABCO-catalyzed Cloke–Wilson rearrangement of different aryl- and alkyl-substituted cyclopropanes by applying the ASM-EDA approach. Finally, and for the sake of completeness, the mechanism and the origin of the stereoselectivity for the strongly related DABCO-catalyzed Cloke–Wilson rearrangement involving vinylcyclopropanes (VCPs) are revisited.

## Results and Discussion

### Reaction Mechanism

**Figure 1:**
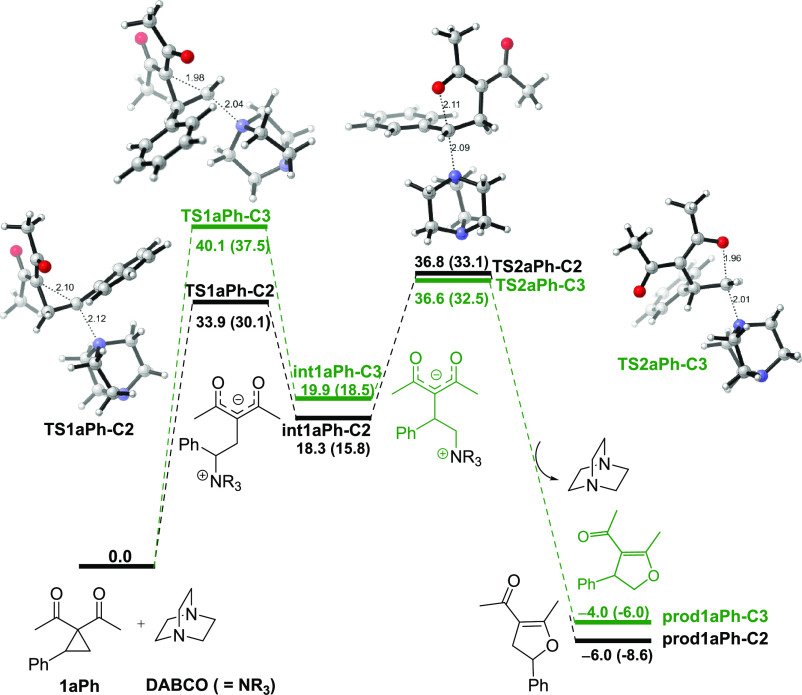
Computed reaction profile for the DABCO-catalyzed Cloke–Wilson rearrangement of phenyl-substituted cyclopropyl ketone **1aPh**. The black-dashed lines describe the reaction pathways associated with the initial nucleophilic attack at the C2-site, while the green-dashed lines represent the energetically disfavored ones associated with the initial C3-attack. Relative free energies (Δ*G*, at 298 K) and bond distances are given in kcal/mol and angstroms (Å), respectively. All data were computed at the SMD(toluene)-M06-2*X*/6-31+G(d,p) level. Values within parentheses refer to SMD(toluene)-ωB97xD/6-31+G(d,p) values. Complete numerical data in the SI, Table S2.

We first explored the DABCO-catalyzed Cloke–Wilson rearrangement of the phenyl-substituted cyclopropyl ketone **1aPh** as a representative system of the experimental reaction described by Xu and co-workers.^10^Figure 1 shows the reaction profiles computed for the formation of the two isomeric 2,3-dihydrofurans, namely, the 2-substituted derivative **prod1aPh–C2**, formed exclusively in the experiments, and the corresponding 3-substituted isomer, denoted as **prod1aPh–C3**. Our calculations indicate that the formation of 2,3-dihydrofuran **prod1aPh–C2** involves the initial nucleophilic attack of the DABCO catalyst to the more sterically hindered C2 (tertiary) site via the transition state **TS1aPh–C2** (Δ*G*^‡^ = 33.9 kcal/mol) leading to the endergonic formation (Δ*G* = 18.3 kcal/mol) of the zwitterionic intermediate **int1aPh–C2**. Then, this species can undergo an intramolecular nucleophilic ring closure following a *5-exo-tet* trajectory via **TS2aPh–C2** to form the observed dihydrofuran **prod1aPh–C2** with the concomitant release of DABCO as a leaving group. This intramolecular cyclization step is predicted to have an activation barrier of 18.5 kcal/mol (relative to **int1aPh–C2**) and is exergonic by 6.0 kcal/mol (relative to the separate reactants), which compensates for the endergonicity of the initial step, driving the entire process forward.

On the other hand, the green profile in Figure 1 describes the pathway associated with the formation of the regioisomeric 2,3-dihydrofuran involving the initial nucleophilic attack at the less hindered C3 (secondary) site of the cyclopropyl moiety. Similar to the formation of **prod1aPh–C2**, this nucleophilic ring-cleavage step proceeds via **TS1aPh–C3** with a barrier of 40.1 kcal/mol leading to the endergonic formation (Δ*G* = 19.9 kcal/mol) of the corresponding zwitterionic intermediate **int1aPh–C3**. This process is followed by an intramolecular nucleophilic ring closure following a *5-exo-tet* trajectory through **TS2aPh–C3** (Δ*G*^‡^ = 16.7 kcal/mol, relative to the zwitterionic intermediate **int1aPh–C3**) leading to the exergonic formation of dihydrofuran **prod1aPh–C3** (Δ*G* = −4.0 kcal/mol relative to the separate reactants).

By comparing the computed energy pathways associated with the formation of both regioisomeric dihydrofurans, it becomes evident that the formation of **prod1aPh–C2** is both kinetically and thermodynamically favored and the initial homoconjugate addition step constitutes the regio-determining step along the PES. In addition, the initial ring-opening step exhibits a relatively high activation barrier, which is consistent with the experimental findings that this process requires the use of harsh conditions such as high temperatures (120 °C) and long reaction times (15–48 h).^10^

### Origin of the Regioselectivity

**Scheme 2:**
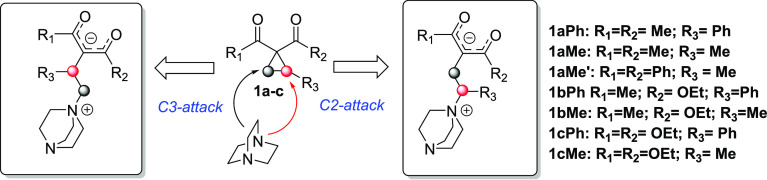
Regioselective Pathways Associated with the Nucleophilic Ring Cleavage of Donor–Acceptor Cyclopropanes Catalyzed by DABCO

We next investigated the factors controlling the regioselectivity observed in the DABCO-catalyzed Cloke–Wilson rearrangement. In this context, the nature of the donor group attached to the C2-site was previously found to significantly affect the reaction outcome.^8^ For instance, Danishefsky and Rovnyak reported that the ring-opening reactions of 2-alkylcyclopropane-1,1-diesters with *N*-nucleophiles proceed with low regioselectivity, yielding a mixture of products associated with the initial homoconjugate addition on both C2- and C3-sites.^16^ Interestingly, Sato and co-workers showed that the ring-opening reactions of DA-cyclopropanes bearing a phenyl group as the donor fragment take place with high selectivity forming exclusively the acyclic product derived from the C2-attack.^17^ Therefore, to rationalize the site selectivity at the crucial homoconjugate addition step, TS structures were located for a set of nucleophilic ring-opening reactions involving some representative methyl- and phenyl-substituted cyclopropanes as donor fragments (Scheme 2), and the resulting selectivities (ΔΔ*G*^‡^ values)^18^ are summarized in Table 1. For illustrative purposes, the computed regioisomeric TS geometries for cyclopropanes analogs to **1aPh** (**1bPh** and **1cPh**) and their corresponding methyl-substituted derivatives (**1aMe**, **1bMe**, and **1cMe**) are presented in the Supporting Information (SI, Figures S2, S3 and S4).

**Table 1:** Activation Energy (at 298 K) Differences between the C2 and C3 DABCO Attacking Sites for the Studied Systems^a^

entry	cyclopropane	ΔΔ*G*‡ (kcal/mol)	ΔΔ*E*‡ (kcal/mol)
1	**1aPh**	6.3	6.3
2	**1bPh**	6.9	6.6
3	**1cPh**	5.3	6.2
4	**1aMe**	0.5	0.5
5	**1aMe’**	1.3	1.3
6	**1bMe**	1.4	1.6
7	**1cMe**	1.6	1.0

a All values were computed at the SMD(toluene)-M06-2*X*/6-31+G(d,p) level.

The analysis of the resulting regioisomeric TS structures reveals that for phenyl-substituted cyclopropanes (compounds **1aPh**, **1bPh,** and **1cPh** in Scheme 2), the computed ΔΔ*G*^‡^ values range from 5.3 to 6.9 kcal/mol (see Table 1, entries 1–3), in good agreement with previous theoretical and experimental findings that C2 is the preferred site for the homoconjugate addition step.^10,19^ Although the presence of an alkyl substituent at the C2-position (compounds **1aMe**, **1aMe′**, **1bMe**, and **1cMe**) significantly reduces the free activation barrier difference (ΔΔ*G*^‡^ values ranging from 0.5 to 1.6 kcal/mol, Table 1, entries 4–7; see also data in Table S5 in the Supporting Information), the addition to the C2-position is still preferred in these species. Interestingly, our calculations reveal that the presence of an alkyl group at the C2 also entails an increase in the activation barriers compared to its C2-unsubstituted cyclopropyl ketone analog (see the SI, entry **TS1aH–CH** in Table S3), which qualitatively agrees with the lower experimental yields reported by Xu and co-workers for the ring rearrangement of both the methyl-substituted and unsubstituted DA-cyclopropanes.^10^ Although the change of vicinal donor and acceptor functionalities affects the relative free energies for the C2-and C3-approaches, there are no appreciable changes in the computed C1–C3 and C1–C2 bond lengths for the different DA-cyclopropane rings under study (Δ*r* < 5 pm, see the SI, Table S6), suggesting that the inherent polarization of the C–C bond vicinally substituted with donor and acceptor groups is not the main factor behind the observed regioselectivity.^20^

**Figure 2:**
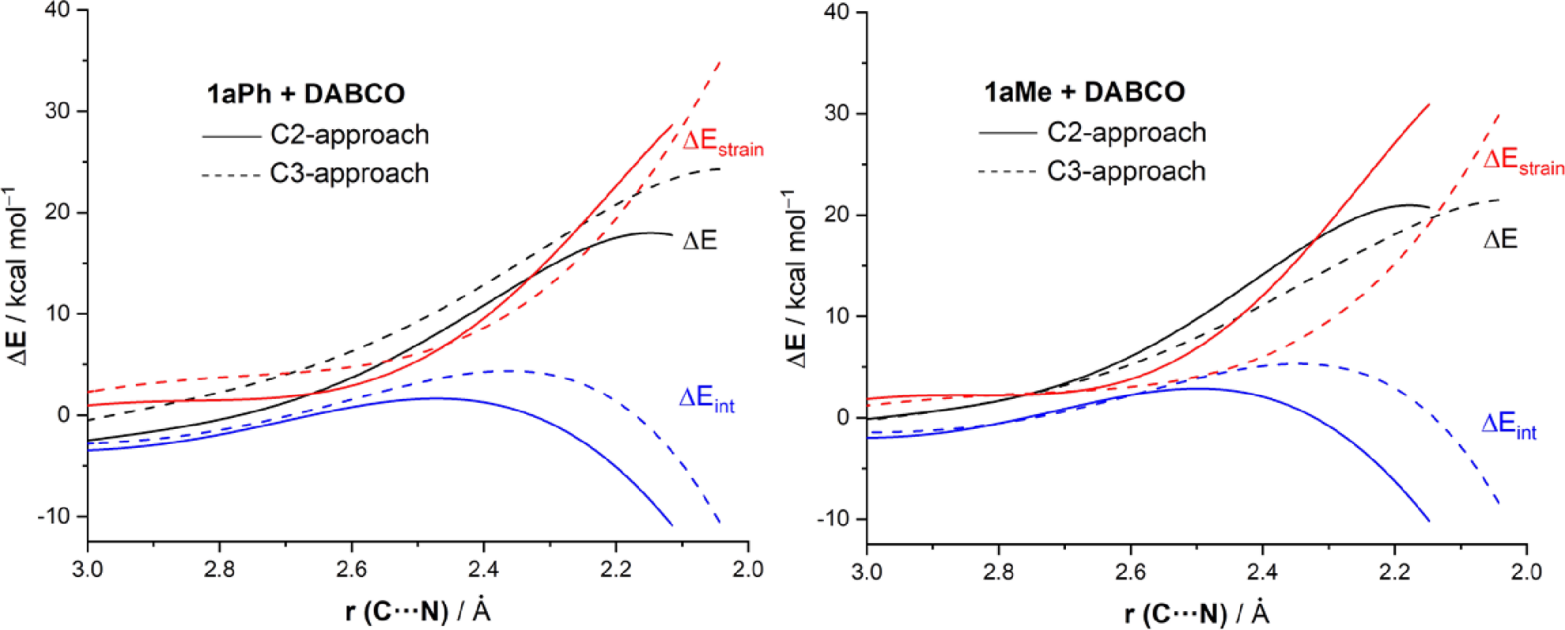
Comparative activation strain diagrams for the ring-opening reaction of cyclopropyl ketones **1aPh** (left) and **1aMe** (right) involving a nucleophilic attack at the C2-site (solid lines) and C3-site (dotted lines), projected onto the C···N bond-forming bond. All data have been computed at the SMD(toluene)-M06-2*X*/6-31+G(d,p) level.

To gain more quantitative insight into the factors governing the regioselectivity of this process, the ASM of reactivity was applied next. Figure 2 compares the activation strain diagrams (ASDs) computed for the C2-attack (solid lines) and C3-attack (dotted lines) for the nucleophilic ring opening of substrates **1aPh** and **1aMe**, from the beginning of the processes up to the corresponding transition states, and projected onto the C···N bond-forming distance. The resulting ASDs for substrate **1aPh** (Figure 2, left) reveal that the required strain energy to achieve the respective TS structure is rather similar for both approaches and even slightly less destabilizing for the C3-pathway. Therefore, the Δ*E*_strain_ term is not at all responsible for the observed C2-regioselectivity. At variance, the C2-pathway benefits from a stronger interaction between the deformed reactants practically along the entire reaction coordinate and particularly in the TS region. For instance, at the same consistent C···N bond-forming distance of 2.2 Å,^21^ ΔΔ*E*_int_ = 6.6 kcal/mol favoring the C2-approach whereas ΔΔ*E*_strain_ = 3.3 kcal/mol favoring the C3-approach. Thus, the preference for the C2-pathway derives solely from the stronger interaction between the deformed reactants computed for this reaction path.

Similarly, for substrate **1aMe**, the C2-approach also benefits from a stronger interaction along the entire reaction coordinate (Figure 2, right). However, in this case, the strain term is much less destabilizing for the C3-approach, which nearly offsets the more stabilizing Δ*E*_int_ for the C2-approach. For instance, at the same consistent C···N bond-forming distance of 2.2 Å,^21^ ΔΔ*E*_int_ = 9.2 kcal/mol favoring the C2-approach whereas ΔΔ*E*_strain_ = 11.8 kcal/mol favoring the C3-approach, which reduces the barrier difference between both pathways. Similar results are obtained for systems **1bPh**, **1bMe** and **1cPh**, **1cMe** (see Figures S6 and S7 in the SI). Therefore, it can be concluded that the C2-approach always benefits from a stronger interaction between the reactants regardless of the nature of the substituent. However, the strain term shows a clear dependence on the substitution and is responsible for the lower barrier difference observed for the alkyl-substituted systems.

**Figure 3:**
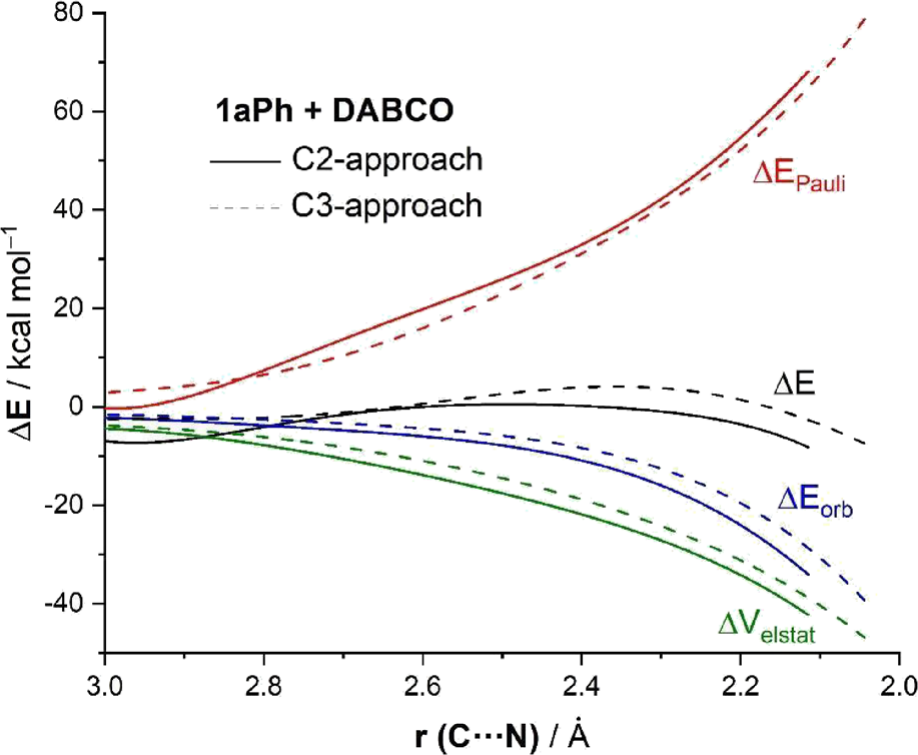
Decomposition of the interaction energy along the reaction pathway for the C2-attack (solid lines) and C3-attack (dotted lines) associated with the initial homoconjugate addition involving cyclopropane **1aPh** and projected onto the C···N bond-forming distance. All data were computed at the ZORA-M06-2X/TZ2P//SMD(toluene)-M06-2*X*/6-31+G(d,p) level.

The EDA was then applied to understand, in a quantitative manner, the origin of the stronger interaction between the deformed reactants for the preferred C2-approach. Figure 3 graphically shows the evolution of the EDA terms along the reaction coordinate for both pathways involving **1aPh** once again from the initial stages of the transformation up to the corresponding TSs. From the data in Figure 3, it becomes evident that the stronger interaction computed for the C2-pathway results from both stronger electrostatic attractions and orbital interactions between the deformed reactants and not from the Pauli repulsion term, which is actually less destabilizing for the C3-pathway. For instance, at the same consistent C···N bond-forming distance of 2.2 Å,^21^ ΔΔ*V*_elstat_ = 2.9 kcal/mol and ΔΔ*E*_orb_ = 4.5 kcal/mol, both favoring the C2-approach, whereas ΔΔ*E*_Pauli_ = 2.5 kcal/mol favoring the C3-approach. Therefore, the stronger interaction computed for the C2-approach, which is mainly responsible for the observed complete regioselectivity of the process, results from stronger orbital interactions and, to a lesser extent, also from more stabilizing electrostatic interactions between the deformed reactants along the entire reaction coordinate.

**Figure 4:**
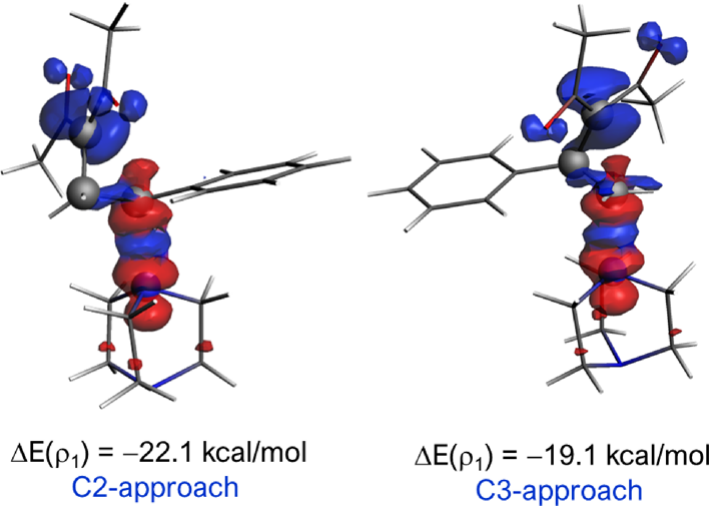
Contour plots of the NOCV deformation densities ρ (isosurface value of 0.002 au) and the associated energies Δ*E*(ρ) for the main orbital interaction present at the initial homoconjugate addition involving cyclopropane **1aPh**. The electronic charge flowed from red to blue. All data have been computed at the ZORA-M06-2X/TZ2P//SMD(toluene)-M06-2*X*/6-31+G(d,p) level.

The critical role of the orbital interactions can be further analyzed with the help of the natural orbital for chemical valence (NOCV) extension of the EDA.^22^ This method allows us to not only visualize but also quantify the main orbital interactions contributing to the total Δ*E*_orb_ term. The NOCV approach indicates, as expected, that the main orbital interaction in both approaches involves the electron flow from the HOMO(DABCO), which corresponds to the lone pair at the nitrogen atom, to the acceptor σ*(C–C) molecular orbital involving the cyclopropyl ketone moiety (denoted as ρ_1_, Figure 4). Interestingly, this interaction is stronger for the pathway involving the C2-approach along the entire reaction coordinate, which significantly contributes to the stronger interaction computed for the C2-pathway. For instance, at the same consistent C···N bond-forming distance of 2.2 Å, the stabilization energy involving this LP(N) → σ*(C–C) interaction is clearly stronger for the C2-pathway (Δ*E*(ρ_1_) = −22.1 kcal/mol) than for the analogous C3-pathway (Δ*E*(ρ_1_) = −19.1 kcal/mol, Figure 4).

### Revisiting the Mechanism of the DABCO-Catalyzed Cloke–Wilson Rearrangement of VCPs

Even though the low-lying pathway described in Figure 1 appears as the predominant mechanism for the Cloke–Wilson rearrangement, the mechanistic picture could be rather different when dealing with VCPs as substrates. In this regard, Xu and co-workers also studied the DABCO-catalyzed rearrangement of an enantioenriched VCP affording the corresponding 2,3-dihydrofuran with almost complete racemization.^10^ Therefore, this stereochemical outcome strongly disfavors a mechanism consisting of two consecutive S_N_2-type nucleophilic substitutions occurring onto the same carbon atom, which should proceed in a stereospecific fashion.^23^

**Scheme 3:**
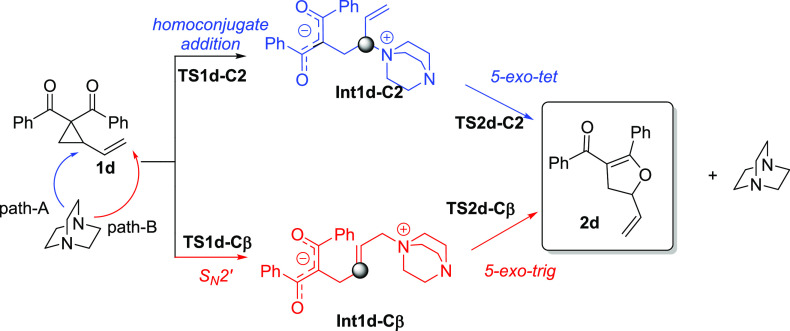
Mechanistic Pathways for the DABCO-Catalyzed Cloke–Wilson Rearrangement of Vinylcyclopropane **1d**

To rationalize this stereochemical issue, we assessed the energetic feasibility of an alternative mechanism involving the nucleophilic attack at the less substituted sp^2^-hybridized vinyl carbon atom in VCP **1d** (i.e., an S_N_2′ ring-opening mechanism) followed by a *5-exo-trig* ring-closing step. This alternative mechanistic scenario is denoted as **Path-B** in Scheme 3 (red pathway) whereas the original proposal, involving the addition to C2,^10^ is defined as **Path-A** (blue pathway in Scheme 3).

**Figure 5:**
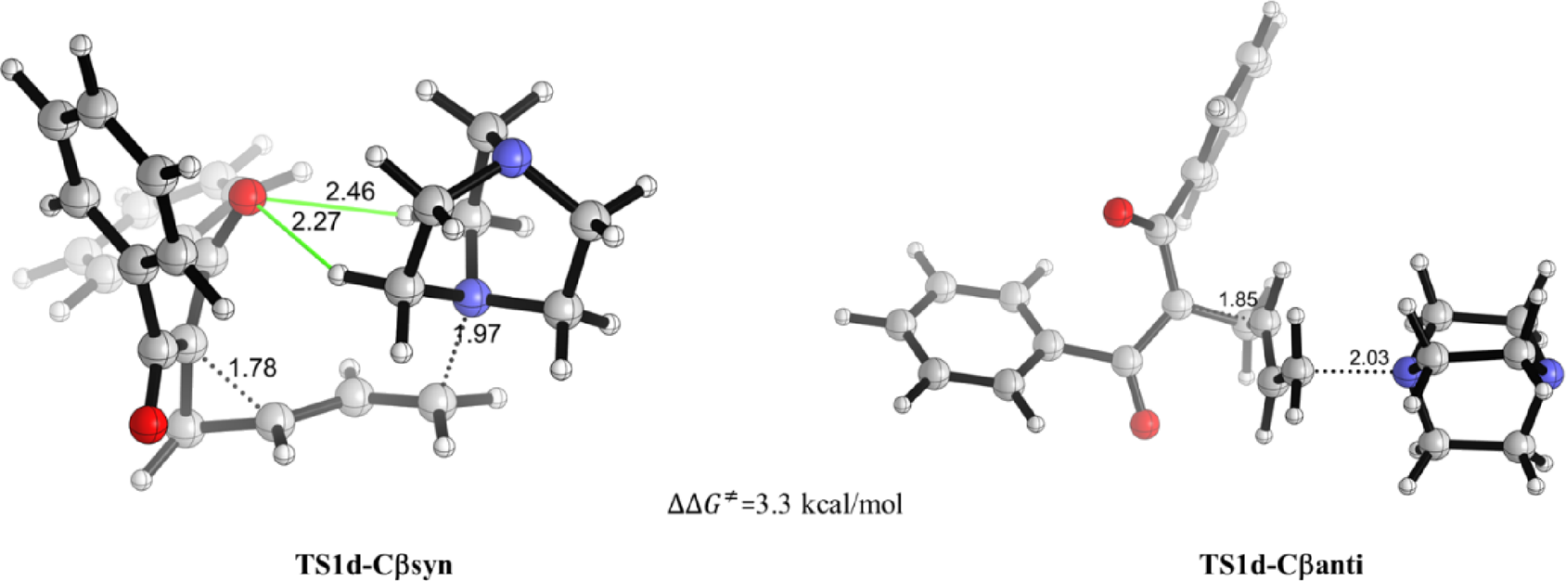
Different orientations of the attacking nucleophile for the S_N_2′ ring opening of cyclopropyl ketone **1d**. Key bond lengths are given in angstroms (Å). Activation free energy difference (computed selectivities, ΔΔ*G*^‡^, at 298 K) is also displayed in the figure. Possible unconventional hydrogen bonds are represented by green lines. All data were computed at the SMD(DMSO)-M06-2*X*/6-31+G(d,p) level.

The initial S_N_2′ ring-opening step taking place via **TS1d-Cβ** may proceed through two competitive reaction channels depending on the orientation of the attacking nucleophile with respect to the cyclopropyl moiety. In the first one, denoted as *syn*-attack, the nucleophile and cyclopropyl ring are placed on the same side (**TS1d-Cβsyn)**, whereas the alternative approach, denoted as *anti-*attack, involves the attacking nucleophile antiperiplanar to the cyclopropyl ring (**TS1d-Cβanti)**. The resulting *syn-* and *anti*-TS structures for the S_N_2′ ring-opening reaction of VCP **1d** are presented in Figure 5.

**Figure 6:**
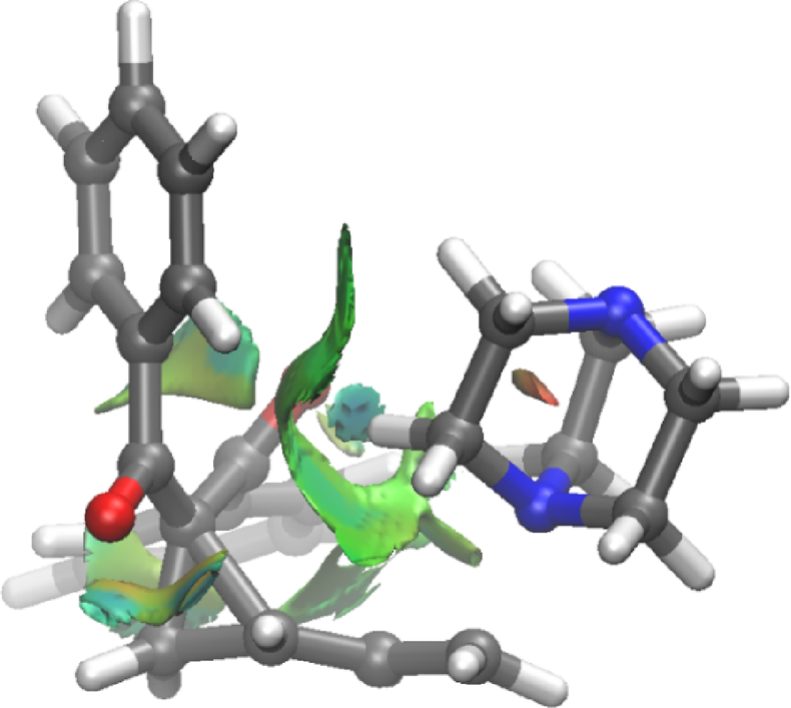
NCIplot isosurfaces for all noncovalent interactions present at the **TS1d-Cβ***syn* structure computed at the SMD(DMSO)-M06-2*X*/6-31+G(d,p) level surfaces (isosurface value of 0.04 au).

Analysis of the corresponding TS structures reveals that the *anti*-approach (**TS1d-Cβ***anti*) is 3.3 kcal/mol less favorable than the *syn*-attack (**TS1d-Cβ***syn*). This kinetic preference is even predicted by other dispersion-corrected density functionals,^24^ with ΔΔ*E*^‡^ (*anti*-*syn*) values ranging from 3.8 to 5.3 kcal/mol, and exhibits a low dependence concerning the basis set size (see the SI, Table S9), confirming that the *syn*-approach is consistently the preferred reaction pathway. At first glance, this *syn* selectivity could be ascribed to the occurrence of stabilizing noncovalent interactions involving the developing oxyanion at the TS region. Indeed, the optimized structure for **TS1d-Cβ***syn* reveals two C–H···O interactions with lengths of 2.27 and 2.46 Å (green lines in Figure 5, left), which are significantly shorter than the sum of the van der Waals radii of hydrogen and oxygen atoms (∼2.7 Å), suggesting an unconventional C–H···O hydrogen bond.^25^ The relative strength of these unconventional hydrogen bonds was estimated by means of second-order perturbation theory (SOPT) energy analysis of the natural bond orbital (NBO) method^26^ at the SMD(DMSO)-M06-2*X*/6-31G+(d,p) level. In **TS1d-Cβ***syn*, the SOPT-NBO method locates two significant donor–acceptor interactions, involving mainly LP(O) → σ*(C–H) molecular orbital interactions, whose associated SOPT-stabilization energies (Δ*E*^2^) amount −4.4 and −2.1 kcal/mol, for the O···H–C bonds of 2.27 and 2.46 Å, respectively (more details in the SI, Table S7). Conversely, similar stabilizing DA interactions were not found in **TS1d-Cβ***anti*, which is not surprising considering the much longer spatial separation. Therefore, it can be concluded that the *syn* approach, which in principle should be disfavored because of higher steric congestion, is preferred due, at least in part, to the occurrence of these stabilizing C–H···O unconventional hydrogen bond interactions. Complementary to the SOPT results, we carried out a topological analysis based on the NCI introduced by Johnson and co-workers (Figure 6).^27^ This analysis enables visualization of these weak interactions in real space, allowing the characterization of both the strength and nature of these interactions. In the NCI analysis on **TS1d-Cβ***syn*, the existence of a molecular region displaying weak attractive interactions (green isosurfaces) associated with each unconventional C–H···O hydrogen bond interaction is clearly confirmed.

**Figure 7:**
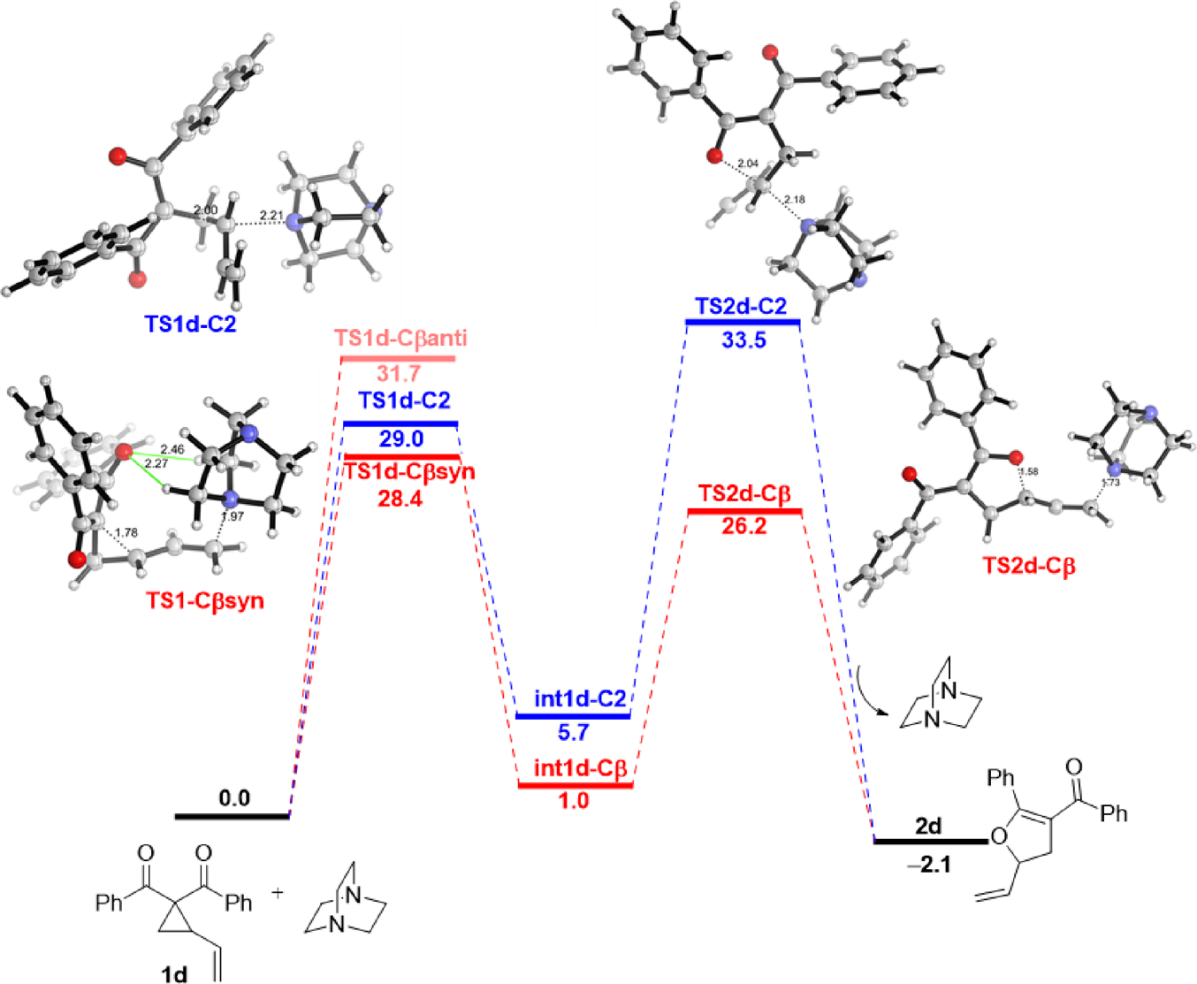
Computed reaction profile for the DABCO-catalyzed Cloke–Wilson rearrangement of cyclopropyl ketone **1d**. The original proposal (involving the homoconjugate addition) is shown in blue, whereas the alternative reaction pathway (involving the S_N_2′) is shown in red. Relative free energies (Δ*G*, at 298 K) and bond distances are given in kcal/mol and angstroms (Å), respectively. All data have been computed at the SMD(DMSO)-M06-2*X*/6-31+G(d,p) level. Complete numerical data are given in the SI, Table S8.

Having established the energetically favored approach for the nucleophilic ring opening of VCP **1d**, through a S_N_2′ mechanism, the stationary points for the overall catalytic transformation were then explored. The resulting free energy diagram for both competitive reaction pathways, i.e., homoconjugate addition vs S_N_2′, is shown in Figure 7.

According to the data in Figure 7, **Path-A** begins with nucleophilic attack via homoconjugate addition onto the C2-site of the cyclopropyl ketone **1d**, which is again favored over the analogous addition at the C3-site by 5.2 kcal/mol. This nucleophilic attack step has an activation barrier of 29.0 kcal/mol (via **TS1d-C2**) and leads to the slightly endergonic (Δ*G* = 5.7 kcal/mol) formation of the open-chain intermediate **int1d-C2**. Then, this zwitterionic intermediate can undergo an intramolecular nucleophilic attack with the departure of DABCO as a leaving group following a *5-exo-tet* cyclization mechanism (via **TS2d-C2**) with an activation barrier of Δ*G*^‡^ = 27.8 kcal/mol (relative to **int1d-C2**), yielding 33.5 kcal/mol as the overall barrier and leading to the exergonic (Δ*G* = −2.1 kcal/mol) formation of the observed 2,3-dihydrofuran **2d**. On the other hand, the nucleophilic ring opening for the low-lying reaction channel associated with **Path-B** (i.e., the *syn*-approach) proceeds with a slightly lower barrier of 28.4 kcal/mol (via **TS1d-Cβ***syn*) to afford the zwitterionic intermediate **int1d-Cβ** in an almost thermoneutral reaction (Δ*G* = 1.0 kcal/mol). This intermediate evolves to **2d** through *5-exo-trig* cyclization with a free energy barrier of 25.2 kcal/mol via **TS2d-Cβ** (with 26.2 kcal/mol as the overall barrier). Therefore, the analysis of the overall reaction pathways reveals that the S_N_2′ mechanism, although only slightly kinetically favored over the corresponding S_N_2 pathway in the initial nucleophilic ring-opening step (ΔΔ*G*^‡^ = 0.6 kcal/mol), is clearly preferred along the entire reaction coordinate and particularly during the subsequent cyclization step. Thus, our calculations strongly suggest that this alternative mechanism is the most likely reaction pathway for the Cloke–Wilson rearrangement involving VCPs.

**Scheme 4:**
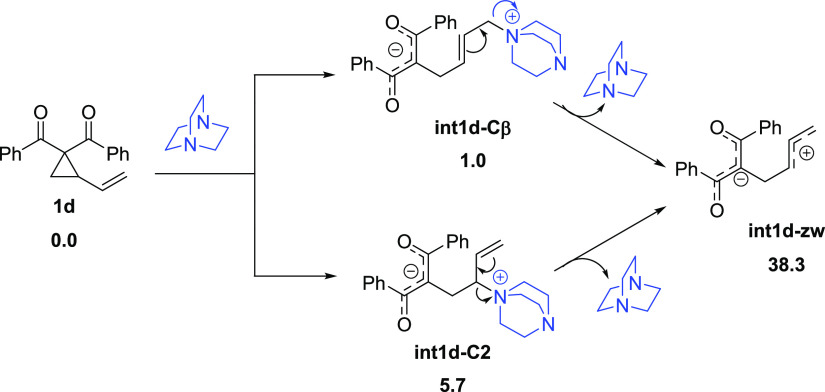
Relative Free Energies (Δ*G*, at 298 K, in kcal/mol) Computed for the Hypothetical Formation of the Allilyc-Like Carbocation Intermediate **int1d-zw** All data have been computed at the SMD(DMSO)-M06-2*X*/6-31+G(d,p) level.

**Figure 8:**
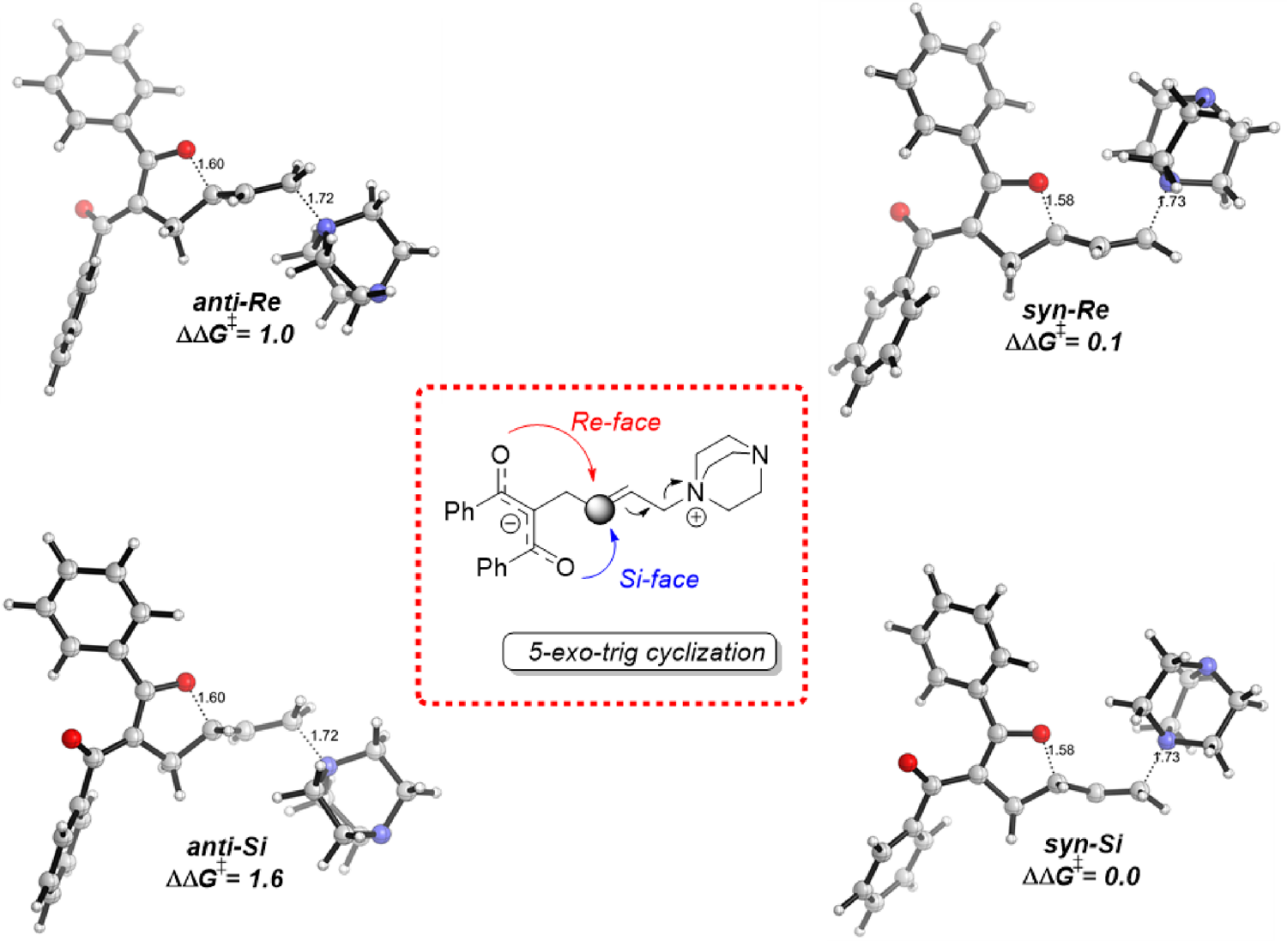
Computed SMD(DMSO)-M06-2*X*/6-31+G(d,p) stereoisomeric transition state structures associated with the *5-exo-trig* cyclization of intermediate **int1d-Cβ** of cyclopropyl ketone **1d** (via **TS2d-Cβ**). Key bond lengths are given in angstroms (Å), and energies are given in kcal/mol. Relative activation free energy differences (ΔΔ*G*^‡^, at 298 K) with respect to *syn*-Si are also displayed in the figure. Complete numerical data are in the SI, Table S10.

According to the profile depicted in Figure 7, the cyclization step involving the zwitterionic intermediate **int1d-Cβ** through **TS2d-Cβ** determines the stereochemical outcome for the Cloke–Wilson rearrangement. In this intermediate, the oxyanion moiety can attack either at the *Re*-face or *Si*-face of the vinyl fragment, leading to four possible stereoisomeric TS structures depending also on the relative orientation of the departing leaving group (*syn* or *anti*). The analysis of the resulting TS structures (Figure 8) reveals that (i) the *syn*-approach is favored over the *anti*-pathway and, (ii) strikingly, the relative difference in the activation barriers (ΔΔ*G*‡ values) for the two low-lying stereodetermining TS structures is only 0.1 kcal/mol. These results fully agree with the experimental findings that the formation of the corresponding 2,3-dihydrofurans proceeds without π-facial discrimination, leading to a nearly racemic mixture of enantiomers (2% ee) when using the enantioenriched (94% ee) VCP **1d**.^10^ It is worth mentioning that this stereochemical outcome was rationalized by Xu and co-workers on the basis of a plausible S_N_1-like mechanism. However, this mechanistic picture can be ruled out because the formation of the possible allylic-like carbocation intermediate (**int1d-zw**) is highly endergonic (Scheme 4), lying 12.1 kcal/mol above the corresponding *5-exo-trig***TS2d-Cβ** structure (see Figure 7).

**Figure 9:**
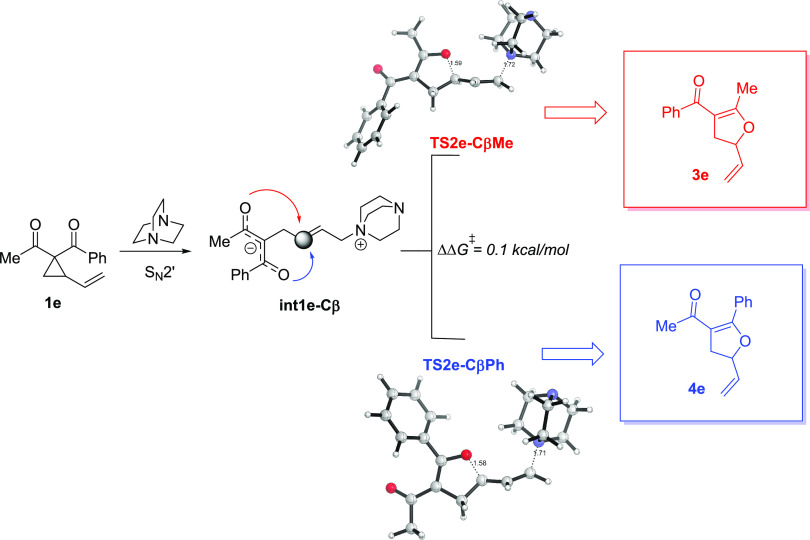
Lowest-energy transition state structures for the 5-*exo-trig* cyclization of cyclopropyl ketone **1e**. Activation free energy difference (ΔΔ*G*^‡^, at 298 K) with respect to **TS2e-CβMe** and bond lengths are given in kcal/mol and angstroms, respectively. All data have been computed at the SMD(DMSO)-M06-2*X*/6-31+G(d,p) level.

Remarkably, this newly proposed mechanism not only rationalizes the stereochemical outcome of the process but also cogently reproduces the regioselectivity patterns observed for cyclopropyl ketones bearing different geminal carbonyl groups. For instance, Xu and co-workers also reported that the VCPs substituted with acetyl and benzoyl groups (vinylcyclopropane **1e**) furnish 2,3-dihydrofurans with an experimental ratio of 57:43 favoring the cyclization through the acetyl oxyanion functionality, as depicted in Figure 9.^10^ According to our proposed S_N_2′ mechanism, the initially formed zwitterionic intermediate **int-1e** may react at either the acetyl (**TS2e-CβMe**) or benzoyl (**TS2e-CβPh**) oxyanion moiety affording the regioisomeric dihydrofurans **3e** and **4e**, respectively (Figure 9). The computed activation free energy difference (ΔΔ*G*^‡^) between the transition states associated with the cyclization step is only 0.1 kcal/mol, which translates into a very low selectivity at room temperature of 54:46, almost matching the observed experimental ratio. Note that the resulting optimized structures of the two low-lying regioisomeric TS structures are featured by a *syn*-approach of the DABCO fragment regarding the oxyanion moiety (see Figure S8 in the SI). At variance, in the originally proposed *5-exo-tet* cyclization (see also Figure S9 in the SI),^10^ the computed regioselectivity was clearly overestimated (ca. 98:2 vs experimental 57:43). This further supports that the S_N_2′ ring-opening mechanism followed by the *5-exo-trig* ring-closing step appears as the prevalent mechanism for the DABCO-catalyzed Cloke–Wilson rearrangement of VCPs.

## Conclusions

We herein presented a detailed study of the mechanism and selectivity patterns of the DABCO-catalyzed ring-opening reactions of donor–acceptor cyclopropanes by means of density functional theory calculations. The transformation occurs stepwise involving an initial ring opening of the cyclopropane promoted by a DABCO molecule followed by a ring-closure reaction of the readily formed zwitterionic intermediate, which produces the observed 2,3-dihydrofurans with concomitant regeneration of the catalyst. In line with experimental evidence, our calculations indicate that the nucleophilic attack at the more sterically hindered side of the cyclopropyl moiety (C2-approach) constitutes the dominant pathway for the initial nucleophilic ring-opening step, regardless of the nature of the substituent attached to this position. According to our ASM-EDA analysis, this is mainly due to a significant enhancement of the interaction energy between the deformed reactants for the C2-approach, which mainly derives from a stronger LP(N) → σ*(C–C) orbital interaction together with more stabilizing electrostatic interactions.

On the other hand, for the particular DABCO-catalyzed Cloke–Wilson rearrangement involving VCPs, our calculations revealed that the more favored catalytic pathway involves an initial S_N_2′-type mechanism followed by a *5-exo-trig* cyclization. The initial nucleophilic ring-opening step is mainly controlled by stabilizing C–H···O unconventional hydrogen bond interactions between the catalyst and the substrate, whereas the subsequent *5-exo-trig* cyclization proceeds without facial selectivity, which is fully consistent with the experimental observations.

In summary, our computational study sheds light on the mechanism and origins of the selectivity of the DABCO-catalyzed Cloke–Wilson rearrangement of donor–acceptor cyclopropanes and also provides a new mechanistic rationale for understanding the regio- and stereochemical outcome of the particular process involving VCPs. We hope that the contents of this article are valuable for future developments of this synthetically useful transformation.

## Computational Methods

Full optimization of all stationary structures was carried out using the hybrid meta-GGA M06-2X functional^28^ in conjunction with the 6-31+G(d,p) basis set.^29^ This level of theory is well suited for computing activation barriers and capturing the noncovalent interactions relevant to reaction kinetics and has been proven to provide accurate results for organic chemistry reactions.^30^ Computed selectivities (ΔΔ*G*^‡^ values) at the M06-2*X*/6-31+G(d,p) level were supported by computations with dispersion-corrected functionals^24^ (ωB97XD^31^ and B3LYP^32^-D3(BJ)^33^; see the SI, Tables S4, S5, S9, S11, and S12). Additional single-point energy refinements were carried out at the same DFT level using the larger 6-311++G(3df,3pd) basis sets^29^ for selected steps of the transformation to check the reliability of the selected M06-2*X*/6-31+(d,p) level (see Tables S4, S9, S11, and S12 in the SI). It was found that the relative energy differences are not significant, which indicates that the selected M06-2*X*/6-31+G(d,p) level is sufficient for the purpose of the present study. All species were optimized with SMD corrections to mimic solvation effects^34^ by dimethyl sulfoxide (DMSO) or toluene used as the reaction medium in the experimental study.^10^ Harmonic analysis and intrinsic reaction coordinate calculations^35^ were performed to confirm the nature of the proposed TS geometries. All calculations were performed with the Gaussian 16 suite of programs.^36^

### ASM of Reactivity and EDA

Within the ASM method,^12^ the potential energy surface Δ*E*(ζ) is decomposed along the reaction coordinate, ζ, into two contributions, namely, the strain Δ*E*_strain_(ζ) associated with the deformation (or distortion) required by the individual reactants during the process and the interaction Δ*E*_int_(ζ) between these increasingly deformed reactants:
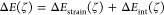


Within the EDA method,^13^ the interaction energy can be further decomposed into the following chemically meaningful terms:
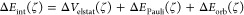


The term Δ*V*_elstat_ corresponds to the classical electrostatic interaction between the unperturbed charge distributions of the deformed reactants and is usually attractive. The Pauli repulsion Δ*E*_Pauli_ comprises destabilizing interactions between occupied orbitals and is responsible for any steric repulsion. The orbital interaction Δ*E*_orb_ accounts for bond pair formation, charge transfer (interaction between occupied orbitals on one moiety and unoccupied orbitals on the other, including HOMO–LUMO interactions), and polarization (empty-occupied orbital mixing on one fragment due to the presence of another fragment). The EDA calculations were carried out in the gas phase with the ADF 2022.103 program package^37^ using the SMD(Solvent)-M06-2*X*/6-31+G(d,p) optimized geometries at the same M06-2X level in conjunction with a triple-ζ-quality basis set using uncontracted Slater-type orbitals augmented by two sets of polarization functions with a frozen-core approximation for the core electrons.^38^ Auxiliary sets of s, p, d, f, and g STOs were used to fit the molecular densities and to represent the Coulomb and exchange potentials accurately in each SCF cycle.^39^ Scalar relativistic effects were incorporated by applying the zeroth-order regular approximation (ZORA).^40^ This level of theory is denoted ZORA-M06-2X/TZ2P//SMD(solvent)-M06-2*X*/6-31+G(d,p). 3D structures were generated by using the CYLview program.^41^

## Data Availability

The data underlying this study are available in the published article and its Supporting Information.

## Supporting Information Available

DFT-optimized geometries of the corresponding regio- and stereodetermining TS structures (Figures S2, S3, S4, S8, and S9), activation strain diagrams (ASDs) for the N–C bond formation step (Figures S5, S6, and S7), ASM calculations (Table S3), C–C bond lengths of cyclopropyl ketones (Table S6), SOPT calculations (Table S7), single-point energy calculations (Tables S4, S5, S9, S11, S12, and S13), relative Gibbs free energies (Tables S2, S8, S10), general structures and nomenclature of substrates (Figure S1 and Table S1), and Cartesian coordinates (in Å) and energies (in Hartrees) of the structures discussed in the text (PDF)
