# Supplementary material for: Mechanistic Insights into the DABCO-Catalyzed Cloke–Wilson Rearrangement: A DFT Perspective
Source: J Org Chem. 2023 Oct 27;88(22):15902–12. doi: 10.1021/acs.joc.3c02011 (PMC10661052; doi:10.1021/acs.joc.3c02011)
Supplement: Supplementary file 1 — jo3c02011_si_001.pdf [file jo3c02011_si_001.pdf]

SUPPORTING INFORMATION

Mechanistic Insights into the DABCO–Catalyzed Cloke–Wilson Rearrangement: A DFT Perspective.

Sebastián Gallardo-Fuentes,[1] Lucas Lodeiro,[2,*] Ricardo Matute,[3] and Israel Fernández[4]

[1]*Instituto de Química, Facultad de Ciencias, Pontificia Universidad Católica de Valparaíso, Avenida Universidad 330, Curauma 2373223, Valparaiso, Chile*

[2]*Departamento de Química, Facultad de Ciencias, Universidad de Chile, Las Palmeras 3425, Ñuñoa 7800003, Santiago, Chile*

[3]*Centro Integrativo de Biología y Química Aplicada (CIBQA), Universidad Bernardo O'Higgins, Santiago 8370854, Chile*

[4]*Departamento de Química Orgánica I and Centro de Innovación en Química Avanzada (ORFEO-CINQA), Facultad de Ciencias Químicas, Universidad Complutense de Madrid, 28040-Madrid, Spain*

* lucas.lodeiro@ug.uchile.cl

CONTENTS

Supporting Information S1

List of Tables S3

List of Figures S7

Origin of the Regioselectivity S10

Revisiting the Mechanism of the DABCO–Catalyzed Cloke–Wilson Rearrangement of VCPs S17

Cartesian coordinates (in Angstroms), total and free energies (in Hartree, at 298 K) S22

LIST OF TABLES

S1 Composition and nomenclature of different substrates under study, according to Figure S1. . . . . S9

S2 Relative free energy (ΔG at 298 K) and total energy profiles for the DABCO–catalyzed Cloke–Wilson rearrangement of phenyl-substituted cyclopropyl ketone **1aPh**, shown in main text Scheme 1 and Figure 1. All quantities in kcal mol^{-1}. In all cases the geometries were optimized at the SMD(Toluene)–M06–2X/6-31G+(d,p) level. Energies of optimized geometries at the SMD(Toluene)–ωB97X–D/6-31G+(d,p) level shown in parenthesis. As additional data, single points at the SMD(Toluene)–M06–2X/6-311++G(3df,3pd) level were computed at the SMD(Toluene)–M06–2X/6-31G+(d,p) optimized geometries and informed in square brackets; Gibbs free energy corrections added from reference optimization level. S10

S3 Activation energy and their decomposition (Activation Strain Model) in strain (substrate, DABCO and system) and interaction terms, for studied systems at the transition state structure, C_2 and C_3 DABCO attacking site. All quantities in kcal mol^{-1}. All data have been computed at the SMD(Toluene)–M06–2X/6-31+G(d,p) level. S10

S4 Activation energy difference, $\Delta\Delta E^{\ddagger}_{C_3-C_2}$, between C_2 and C_3 DABCO attacking site transition state, for studied systems, using different setups: the main text basis set (6-31+G(d,p)) and a bigger one (6-311++G(3df,3pd)) with the hybrid meta–GGA functional M06–2X, and two different functionals, the hybrid GGA functional with Grimme's dispersion correction B3LYP–D3(BJ) and range–separated hybrid GGA functional with Grimme's dispersion correction ωB97X–D, in conjunction with the main text basis set (6-31+G(d,p)). All quantities in kcal mol^{-1}. In all cases the geometries were the SMD(Toluene)–M06–2X/6-31G+(d,p) optimized ones, and the implicit solvent was introduced by means of SMD(Toluene) correction. S11

S5 Activation free energy difference (selectivities), $\Delta\Delta G^{\ddagger}_{C_3-C_2}$ at 298 K, between C_2 and C_3 DABCO attacking site transition state, for studied systems, using the main text basis set (6-31+G(d,p))with the hybrid meta–GGA functional M06–2X, and two different functionals, the hybrid GGA functional with Grimme's dispersion correction B3LYP–D3(BJ) and range–separated hybrid GGA functional with Grimme's dispersion correction ωB97X–D. All quantities in kcal mol^{-1}. In all cases the geometries were optimized at the current level of theory, and the implicit solvent was introduced by means of SMD(Toluene) correction. S11

S6 Bond length (C–C) of cyclopropyl ring for studied substrates at their optimized structures, computed at the SMD(Toluene)–M06–2X/6-31+G(d,p) level. All quantities in Angstroms (Å). S12

S7 Second–Order Perturbation Theory energy analysis for the **TS1d–C$_\beta$*syn*** structure at the SMD(DMSO)–M06–2X/6-31G+(d,p) level, for proposed unconventional hydrogen bonds. Donations (top) and corresponding backdonations (bottom). All quantities in kcal mol^{-1}. S17

S8 Relative free energy (ΔG at 298 K) and total energy profiles for the DABCO–catalyzed Cloke–Wilson rearrangement of vinylcyclopropane **1d**, shown in main text Scheme 3 and Figure 7. All quantities in kcal mol^{-1}. In all cases the geometries were optimized at the SMD(DMSO)–M06–2X/6-31G+(d,p) level. S17

S9 Activation energy difference, $\Delta\Delta E^{\ddagger}_{anti-syn}$, between DABCO *anti*– and *syn*–approach transition state (**TS1d–C$_{\beta}$*anti*** and **TS1d–C$_{\beta}$*syn***, respectively) of **1d** substrate, using different setups: the main text basis set (6-31+G(d,p)) and a bigger one (6-311++G(3df,3pd)) with the hybrid meta–GGA functional M06–2X, and two different functionals, the hybrid GGA functional with Grimme's dispersion correction B3LYP–D3(BJ) and range–separated hybrid GGA functional with Grimme's dispersion correction ωB97X–D, in conjunction with the main text basis set (6-31+G(d,p)). All quantities in kcal mol^{-1}. In all cases the geometries were the SMD(DMSO)–M06–2X/6-31G+(d,p) optimized ones, and the implicit solvent was introduced by means of SMD(DMSO) correction. S18

S10 Free energy and total energy differences ($\Delta\Delta E^{\ddagger}$ at 298 K) for the DABCO–catalyzed Cloke–Wilson rearrangement of vinylcyclopropane **1d** stereoisomeric transition state structures associated with the 5–*exo–trig* cyclization of intermediate **int1d–C$_{\beta}$** via **TS2d–C$_{\beta}$** (w.r.t. *syn–Si*). All quantities in kcal mol^{-1}. In all cases the geometries were optimized at the SMD(DMSO)–M06–2X/6-31G+(d,p) level. The vibrational entropy contribution was computed by means of QRRHO approximation. S18

S11 Total energy difference, $\Delta\Delta E^{\ddagger}$, for the DABCO–catalyzed Cloke–Wilson rearrangement of vinylcyclopropane **1d** stereoisomeric transition state structures associated with the 5–*exo–trig* cyclization of intermediate **int1d–C$_{\beta}$** via **TS2d–C$_{\beta}$** (w.r.t. *syn–Si*), using different setups: the main text basis set (6-31+G(d,p)) and a bigger one (6-311++G(3df,3pd)) with the hybrid meta–GGA functional M06–2X, and two different functionals, the hybrid GGA functional with Grimme's dispersion correction B3LYP–D3(BJ) and range–separated hybrid GGA functional with Grimme's dispersion correction ωB97X–D, in conjunction with the main text basis set (6-31+G(d,p)). All quantities in kcal mol^{-1}. In all cases the geometries were the SMD(DMSO)–M06–2X/6-31G+(d,p) optimized ones, and the implicit solvent was introduced by means of SMD(DMSO) correction. S18

S12 Total energy difference, $\Delta\Delta E^{\ddagger}$, for the DABCO–catalyzed Cloke–Wilson rearrangement of vinylcyclopropane **1e** stereoisomeric transition state structures associated with the 5–*exo–trig* cyclization of intermediate **int1e–C$_\beta$** (w.r.t. **TS2e–C$_\beta$Me–*syn***), using different setups: the main text basis set (6-31+G(d,p)) and a bigger one (6-311++G(3df,3pd)) with the hybrid meta–GGA functional M06–2X, and two different functionals, the hybrid GGA functional with Grimme's dispersion correction B3LYP–D3(BJ) and range–separated hybrid GGA functional with Grimme's dispersion correction ωB97X–D, in conjunction with the main text basis set (6-31+G(d,p)). All quantities in kcal mol^{-1}. In all cases the geometries were the SMD(DMSO)–M06–2X/6-31G+(d,p) optimized ones, and the implicit solvent was introduced by means of SMD(DMSO) correction. S20
S13 Total energy difference, $\Delta\Delta E^{\ddagger}$, for the DABCO–catalyzed Cloke–Wilson rearrangement of vinylcyclopropane **1e** stereoisomeric transition state structures associated with the 5–*exo–tet* cyclization of intermediate **int1e–C$_2$** (w.r.t. **TS2e–C$_2$Me**), using different setups: the main text basis set (6-31+G(d,p)) and a bigger one (6-311++G(3df,3pd)) with the hybrid meta–GGA functional M06–2X, and two different functionals, the hybrid GGA functional with Grimme's dispersion correction B3LYP–D3(BJ) and range–separated hybrid GGA functional with Grimme's dispersion correction ωB97X–D, in conjunction with the main text basis set (6-31+G(d,p)). All quantities in kcal mol^{-1}. In all cases the geometries were the SMD(DMSO)–M06–2X/6-31G+(d,p) optimized ones, and the implicit solvent was introduced by means of SMD(DMSO) correction. S21

LIST OF FIGURES

S1 General structure of donor–acceptor cyclopropanes used to study the Cloke–Wilson rearragement. S9

S2 Computed SMD(Toluene)–M06–2X/6-31+G(d,p) regioisomeric transition state structures for the nucleophilic ring–opening of activated cyclopropanes **1aPh** (top) and **1aMe** (bottom) catalyzed by DABCO. Key bond lengths are given in Angstroms (Å). Activation free energy differences (computed selectivities, $\Delta\Delta G^{\ddagger}_{C_3\text{–}C_2}$ values at 298 K) are also displayed in the figure. S12

S3 Computed SMD(Toluene)–M06–2X/6-31+G(d,p) regioisomeric transition state structures for the nucleophilic ring–opening of activated cyclopropanes **1bPh** (top) and **1bMe** (bottom) catalyzed by DABCO. Key bond lengths are given in Angstroms (Å). Activation free energy differences (computed selectivities, $\Delta\Delta G^{\ddagger}_{C_3\text{–}C_2}$ values at 298 K) are also displayed in the figure. S13

S4 Computed SMD(Toluene)–M06–2X/6-31+G(d,p) regioisomeric transition state structures for the nucleophilic ring–opening of activated cyclopropanes **1cPh** (top) and **1cMe** (bottom) catalyzed by DABCO. Key bond lengths are given in Angstroms (Å). Activation free energy differences (computed selectivities, $\Delta\Delta G^{\ddagger}_{C_3\text{–}C_2}$ values at 298 K) are also displayed in the figure. S14

S5 Comparative activation strain diagrams for the ring–opening reaction of cyclopropyl ketones, **1a** structure, involving a nucleophilic attack at the C_2 site (solid lines) and C_3 site (dotted lines). All data have been computed at the SMD(Toluene)–M06–2X/6-31+G(d,p) level. S15

S6 Comparative activation strain diagrams for the ring–opening reaction of cyclopropyl ester–ketone, **1b** structure, involving a nucleophilic attack at the C_2 site (solid lines) and C_3 site (dotted lines). All data have been computed at the SMD(Toluene)–M06–2X/6-31+G(d,p) level. S15

S7 Comparative activation strain diagrams for the ring–opening reaction of cyclopropyl esters, 1c structures, involving a nucleophilic attack at the C_2 site (solid lines) and C_3 site (dotted lines). All data have been computed at the SMD(Toluene)–M06–2X/6-31+G(d,p) level. S16

S8 Computed SMD(DMSO)–M06–2X/6-31+G(d,p) regioisomeric transition state structures for the DABCO–catalyzed Cloke–Wilson rearrangement of vinylcyclopropane **1e**, 5–*exo–trig* cyclization of intermediate **int1e–C$_{\beta}$** via **TS2e–C$_{\beta}$Me** and **TS2e–C$_{\beta}$Ph**. Key bond lengths are given in Angstroms (Å). Relative free energy differences ($\Delta\Delta G^{\ddagger}$ values at 298 K) are also displayed in the figure. S19
S9 Computed SMD(DMSO)–M06–2X/6-31+G(d,p) regioisomeric transition state structures for the DABCO–catalyzed Cloke–Wilson rearrangement of vinylcyclopropane **1e**, 5–*exo–tet* cyclization of intermediate **int1e–C$_2$** via **TS2e–C$_2$Me** and **TS2e–C$_2$Ph**. Key bond lengths are given in Angstroms (Å). Relative free energy differences ($\Delta\Delta G^{\ddagger}$ values at 298 K) are also displayed in the figure. S20

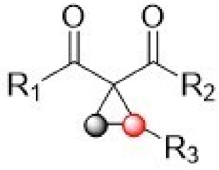

Figure S1. General structure of donor–acceptor cyclopropanes used to study the Cloke–Wilson rearragement.

Table S1. Composition and nomenclature of different substrates under study, according to Figure S1.

Substrate	R_1	R_2	R_3
1aPh	Me	Me	Ph
1aMe	Me	Me	Me
1aMe’	Ph	Ph	Me
1aH	Me	Me	H
1bPh	Me	OEt	Ph
1bMe	Me	OEt	Me
1bH	Me	OEt	H
1cPh	OEt	OEt	Ph
1cMe	OEt	OEt	Me
1cH	OEt	OEt	H
1d	Ph	Ph	$-CH=CH_2$
1e	Me	Ph	$-CH=CH_2$

ORIGIN OF THE REGIOSELECTIVITY

Table S2. Relative free energy (ΔG at 298 K) and total energy profiles for the DABCO–catalyzed Cloke–Wilson rearrangement of phenyl-substituted cyclopropyl ketone **1aPh**, shown in main text Scheme 1 and Figure 1. All quantities in kcal mol^{-1}. In all cases the geometries were optimized at the SMD(Toluene)–M06–2X/6-31G+(d,p) level. Energies of optimized geometries at the SMD(Toluene)–ωB97X–D/6-31G+(d,p) level shown in parenthesis. As additional data, single points at the SMD(Toluene)–M06–2X/6-311++G(3df,3pd) level were computed at the SMD(Toluene)–M06–2X/6-31G+(d,p) optimized geometries and informed in square brackets; Gibbs free energy corrections added from reference optimization level.

Structure	ΔG	ΔE
1aPh + DABCO	0.0 (0.0) [0.0]	0.0 (0.0) [0.0]
TS1aPh–C_2	33.9 (30.1) [35.5]	17.9 (15.0) [19.5]
TS1aPh–C_3	40.1 (37.5) [41.8]	24.1 (22.2) [25.8]
int1aPh–C_2	18.3 (15.8) [20.3]	−1.2 (−3.6) [0.8]
int1aPh–C_3	19.9 (18.5) [21.8]	0.7 (−1.5) [2.7]
TS2aPh–C_2	36.8 (33.1) [38.6]	20.5 (16.2) [22.3]
TS2aPh–C_3	36.6 (32.5) [38.3]	19.3 (16.0) [21.0]
prod1aPh–C_2 + DABCO	−6.0 (−8.6) [−5.3]	−6.9 (−8.5) [−6.2]
prod1aPh–C_3 + DABCO	−4.0 (−6.0) [−3.4]	−5.8 (−7.5) [−5.2]

Table S3. Activation energy and their decomposition (Activation Strain Model) in strain (substrate, DABCO and system) and interaction terms, for studied systems at the transition state structure, C_2 and C_3 DABCO attacking site. All quantities in kcal mol^{-1}. All data have been computed at the SMD(Toluene)–M06–2X/6-31+G(d,p) level.

TS	$\Delta E^{\ddagger}$	$\Delta E^{\ddagger}_{strain,subs}$	$\Delta E^{\ddagger}_{strain,DABCO}$	$\Delta E^{\ddagger}_{strain}$	$\Delta E^{\ddagger}_{int}$
TS1aPh–C_2	17.8	28.4	0.3	28.7	−10.8
TS1aPh–C_3	24.1	34.1	0.3	34.3	−10.2
TS1aMe–C_2	20.8	30.7	0.3	30.9	−10.2
TS1aMe–C_3	21.3	29.3	0.2	29.5	−8.2
TS1aH–C_H	18.5	25.8	0.3	26.1	−7.6
TS1bPh–C_2	16.7	27.1	0.3	27.4	−10.7
TS1bPh–C_3	23.3	33.3	0.3	33.6	−10.2
TS1bMe–C_2	19.4	29.1	0.2	29.4	−10.0
TS1bMe–C_3	21.0	29.1	0.3	29.3	−8.4
TS1bH–C_H	18.2	25.5	0.3	25.8	−7.5
TS1cPh–C_2	21.0	31.7	0.3	32.0	−11.0
TS1cPh–C_3	27.2	37.5	0.3	37.8	−10.6
TS1cMe–C_2	23.6	33.6	0.3	33.9	−10.3
TS1cMe–C_3	24.7	33.1	0.3	33.4	−8.7
TS1cH–C_H	21.3	28.8	0.3	29.2	−7.8

Table S4. Activation energy difference, $\Delta\Delta E^{\ddagger}_{C_3-C_2}$, between C_2 and C_3 DABCO attacking site transition state, for studied systems, using different setups: the main text basis set (6-31+G(d,p)) and a bigger one (6-311++G(3df,3pd)) with the hybrid meta–GGA functional M06–2X, and two different functionals, the hybrid GGA functional with Grimme's dispersion correction B3LYP–D3(BJ) and range–separated hybrid GGA functional with Grimme's dispersion correction ωB97X–D, in conjunction with the main text basis set (6-31+G(d,p)). All quantities in kcal mol^{-1}. In all cases the geometries were the SMD(Toluene)–M06–2X/6-31G+(d,p) optimized ones, and the implicit solvent was introduced by means of SMD(Toluene) correction.

System	M06–2X<br>6-31+G(d,p)	M06–2X<br>6-311++G(3df,3pd)	B3LYP–D3(BJ)<br>6-31+G(d,p)	ωB97X–D<br>6-31+G(d,p)
1aPh	6.3	6.3	9.1	7.1
1aMe	0.5	0.9	1.8	0.7
1bPh	6.6	6.6	9.3	7.4
1bMe	1.6	1.8	3.0	2.1
1cPh	6.2	6.1	8.5	6.7
1cMe	1.0	1.3	2.2	1.4

Table S5. Activation free energy difference (selectivities), $\Delta\Delta G^{\ddagger}_{C_3-C_2}$ at 298 K, between C_2 and C_3 DABCO attacking site transition state, for studied systems, using the main text basis set (6-31+G(d,p))with the hybrid meta–GGA functional M06–2X, and two different functionals, the hybrid GGA functional with Grimme's dispersion correction B3LYP–D3(BJ) and range–separated hybrid GGA functional with Grimme's dispersion correction ωB97X–D. All quantities in kcal mol^{-1}. In all cases the geometries were optimized at the current level of theory, and the implicit solvent was introduced by means of SMD(Toluene) correction.

System	M06–2X<br>6-31+G(d,p)	B3LYP–D3(BJ)<br>6-31+G(d,p)	ωB97X–D<br>6-31+G(d,p)
1aPh	6.3	9.6	7.5
1aMe	0.5	2.4	0.5
1bPh	6.9	9.7	6.1
1bMe	1.4	4.0	1.7
1cPh	5.3	10.7	8.6
1cMe	1.6	3.0	0.8

Table S6. Bond length (C–C) of cyclopropyl ring for studied substrates at their optimized structures, computed at the SMD(Toluene)–M06–2X/6-31+G(d,p) level. All quantities in Angstroms (Å).

Substrate	$r(C_1–C_2)$	$r(C_1–C_3)$	$r(C_2–C_3)$
1aPh	1.55	1.50	1.49
1aMe	1.54	1.51	1.48
1aH	1.53	1.53	1.48
1bPh	1.55	1.51	1.48
1bMe	1.54	1.52	1.48
1bH	1.53	1.53	1.47
1cPh	1.54	1.51	1.49
1cMe	1.53	1.51	1.49
1cH	1.52	1.52	1.48

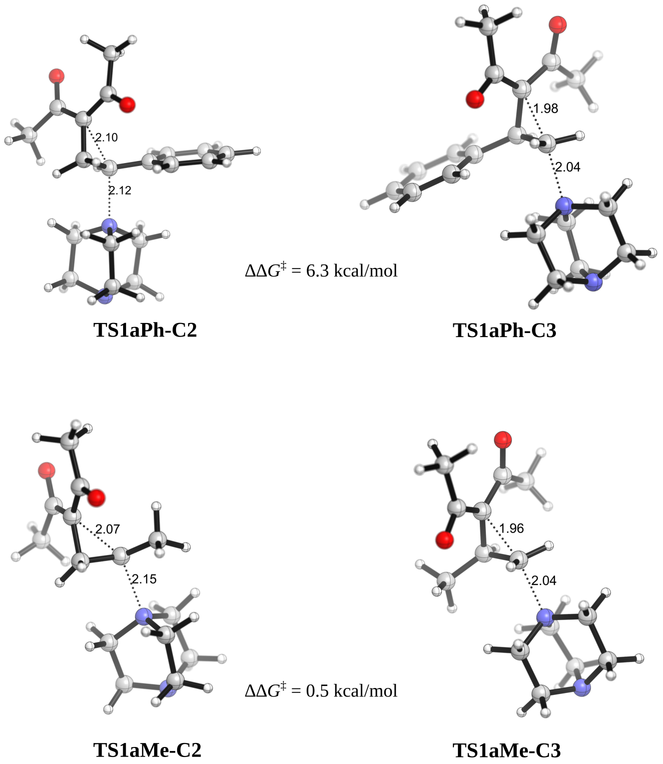

Figure S2. Computed SMD(Toluene)–M06–2X/6-31+G(d,p) regioisomeric transition state structures for the nucleophilic ring–opening of activated cyclopropanes **1aPh** (top) and **1aMe** (bottom) catalyzed by DABCO. Key bond lengths are given in Angstroms (Å). Activation free energy differences (computed selectivities, $\Delta\Delta G^{\ddagger}_{C_3-C_2}$ values at 298 K) are also displayed in the figure.

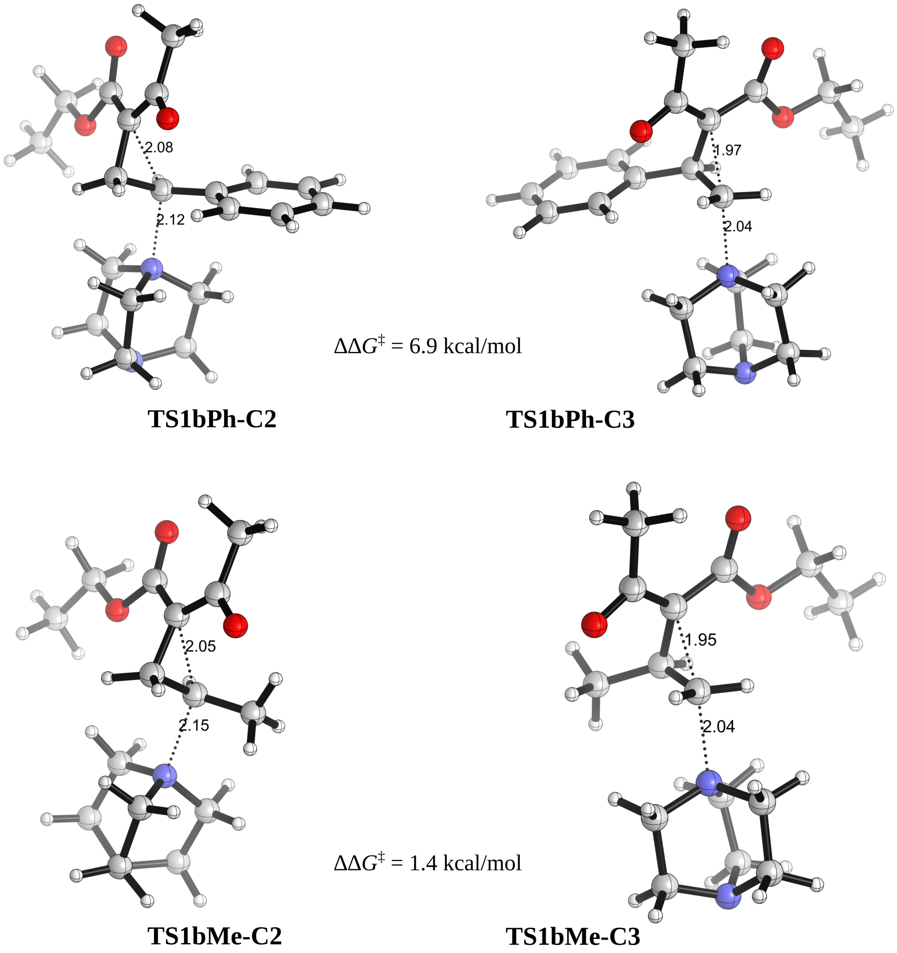

Figure S3. Computed SMD(Toluene)–M06–2X/6-31+G(d,p) regioisomeric transition state structures for the nucleophilic ring–opening of activated cyclopropanes **1bPh** (top) and **1bMe** (bottom) catalyzed by DABCO. Key bond lengths are given in Angstroms (Å). Activation free energy differences (computed selectivities, $\Delta\Delta G^{\ddagger}_{C_3-C_2}$ values at 298 K) are also displayed in the figure.

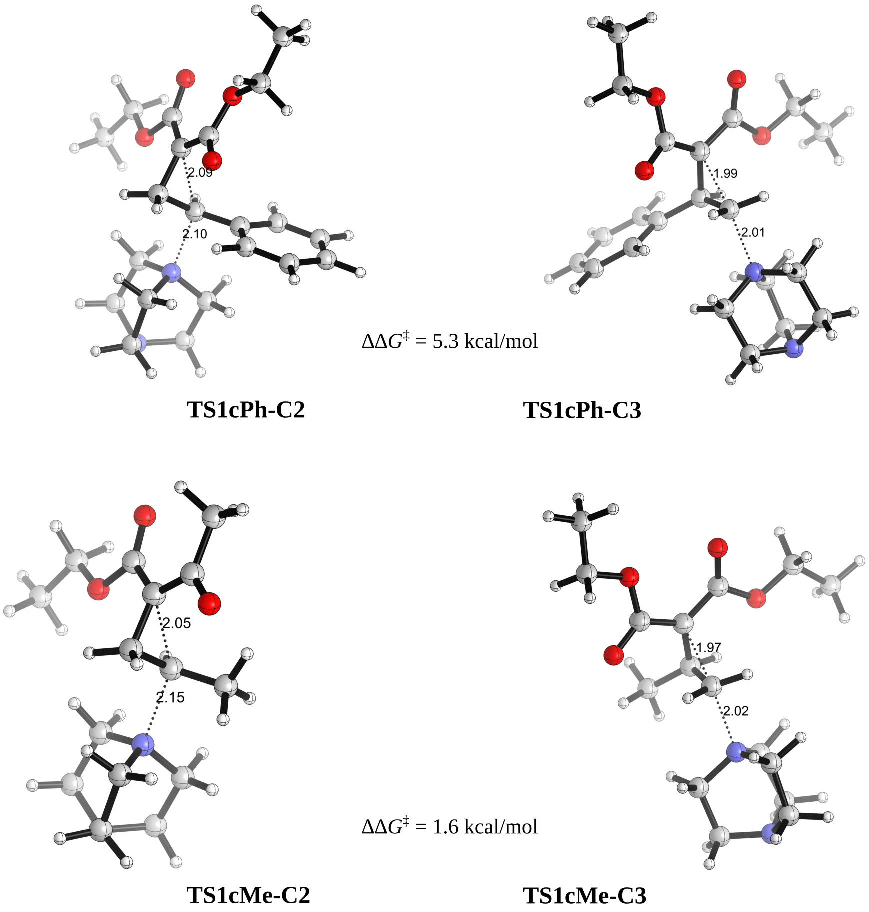

Figure S4. Computed SMD(Toluene)–M06–2X/6-31+G(d,p) regioisomeric transition state structures for the nucleophilic ring–opening of activated cyclopropanes **1cPh** (top) and **1cMe** (bottom) catalyzed by DABCO. Key bond lengths are given in Angstroms (Å). Activation free energy differences (computed selectivities, $\Delta\Delta G^{\ddagger}_{C_3-C_2}$ values at 298 K) are also displayed in the figure.

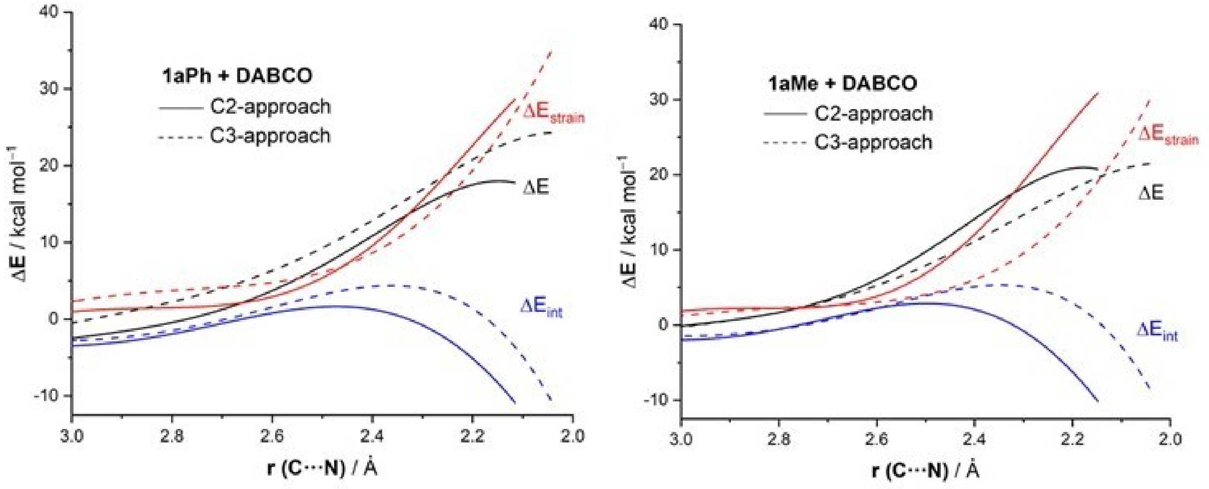

(a) 1aPh   **(b)** 1aMe

Figure S5. Comparative activation strain diagrams for the ring–opening reaction of cyclopropyl ketones, **1a** structure, involving a nucleophilic attack at the C_2 site (solid lines) and C_3 site (dotted lines). All data have been computed at the SMD(Toluene)–M06–2X/6-31+G(d,p) level.

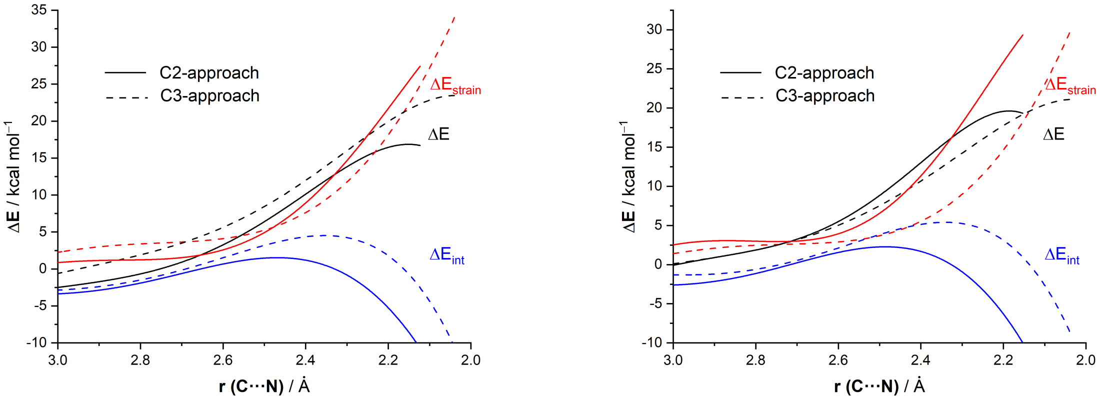

(a) 1bPh   **(b)** 1bMe

Figure S6. Comparative activation strain diagrams for the ring–opening reaction of cyclopropyl ester–ketone, **1b** structure, involving a nucleophilic attack at the C_2 site (solid lines) and C_3 site (dotted lines). All data have been computed at the SMD(Toluene)–M06–2X/6-31+G(d,p) level.

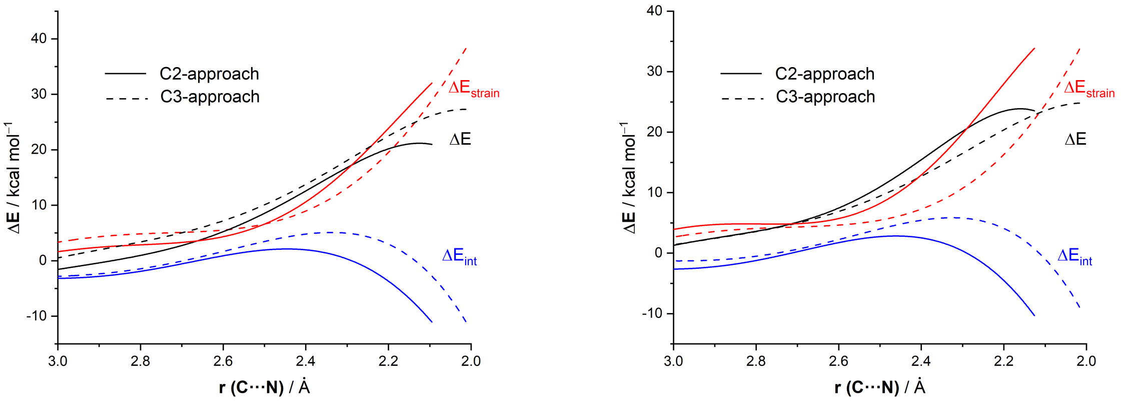

(a) 1cPh **(b)** 1cMe

Figure S7. Comparative activation strain diagrams for the ring–opening reaction of cyclopropyl esters, 1c structures, involving a nucleophilic attack at the C_2 site (solid lines) and C_3 site (dotted lines). All data have been computed at the SMD(Toluene)–M06–2X/6-31+G(d,p) level.

Table S7. Second–Order Perturbation Theory energy analysis for the **TS1d–C$_\beta$*syn*** structure at the SMD(DMSO)–M06–2X/6-31G+(d,p) level, for proposed unconventional hydrogen bonds. Donations (top) and corresponding backdonations (bottom). All quantities in kcal mol^{-1}.

Hydrogen bond, 2.46 Å					Hydrogen bond, 2.27 Å				
Donor	→	Acceptor	Orbitals	$E^{(2)}$	Donor	→	Acceptor	Orbitals	$E^{(2)}$
C–O	→	H	$\sigma \to$ LP*	0.06	C–O	→	H	$\sigma \to$ LP*	0.10
C–O	→	H	$\sigma \to$ LP*	0.32	C–O	→	H	$\sigma \to$ LP*	0.14
C–O	→	H–C	$\sigma \to \sigma^*$	0.94	C–O	→	H–C	$\sigma^* \to \sigma^*$	0.46
C–O	→	H–C	$\sigma^* \to \sigma^*$	0.07	O	→	H–C	LP $\to \sigma^*$	0.90
O	→	H–C	LP $\to \sigma^*$	0.43	O	→	H–C	LP $\to \sigma^*$	2.27
					O	→	H	LP → LP*	0.30
C–H	→	O	$\sigma \to$ LP*	0.15	C–H	→	O	$\sigma \to$ LP*	0.13
C–H	→	O–C	$\sigma^* \to \sigma^*$	0.10	C–H	→	O	$\sigma \to$ LP*	0.05
					C–H	→	O–C	$\sigma \to \sigma^*$	0.06

Table S8. Relative free energy (ΔG at 298 K) and total energy profiles for the DABCO–catalyzed Cloke–Wilson rearrangement of vinylcyclopropane **1d**, shown in main text Scheme 3 and Figure 7. All quantities in kcal mol^{-1}. In all cases the geometries were optimized at the SMD(DMSO)–M06–2X/6-31G+(d,p) level.

Structure	ΔG	ΔE
1d + DABCO	0.0	0.0
TS1d–C$_2$	29.0	15.3
TS1d–C$_\beta$*syn*	28.4	13.9
TS1d–C$_\beta$*anti*	31.7	18.7
int1d–C$_2$	5.7	−12.0
int1d–C$_\beta$*syn*	1.0	−16.7
int1d–C$_\beta$*anti*	2.1	−14.2
TS2d–C$_2$	33.5	18.7
TS2d–C$_\beta$	26.2	10.7
2d + DABCO	−2.1	−3.1

Table S9. Activation energy difference, $\Delta\Delta E^{\ddagger}_{anti-syn}$, between DABCO *anti*– and *syn*–approach transition state (**TS1d–C$_\beta$*anti*** and **TS1d–C$_\beta$*syn***, respectively) of **1d** substrate, using different setups: the main text basis set (6-31+G(d,p)) and a bigger one (6-311++G(3df,3pd)) with the hybrid meta–GGA functional M06–2X, and two different functionals, the hybrid GGA functional with Grimme's dispersion correction B3LYP–D3(BJ) and range–separated hybrid GGA functional with Grimme's dispersion correction ωB97X–D, in conjunction with the main text basis set (6-31+G(d,p)). All quantities in kcal mol^{-1}. In all cases the geometries were the SMD(DMSO)–M06–2X/6-31G+(d,p) optimized ones, and the implicit solvent was introduced by means of SMD(DMSO) correction.

M06–2X 6-31+G(d,p)	M06–2X 6-311++G(3df,3pd)	B3LYP–D3(BJ) 6-31+G(d,p)	ωB97X–D 6-31+G(d,p)
4.8	4.6	3.8	5.3

Table S10. Free energy and total energy differences ($\Delta\Delta E^{\ddagger}$ at 298 K) for the DABCO–catalyzed Cloke–Wilson rearrangement of vinylcyclopropane **1d** stereoisomeric transition state structures associated with the 5–*exo*–*trig* cyclization of intermediate **int1d–C$_\beta$** via **TS2d–C$_\beta$** (w.r.t. *syn*–*Si*). All quantities in kcal mol^{-1}. In all cases the geometries were optimized at the SMD(DMSO)–M06–2X/6-31G+(d,p) level. The vibrational entropy contribution was computed by means of QRRHO approximation.

TS2	$\Delta\Delta G^{\ddagger}$	$\Delta\Delta E^{\ddagger}$
anti–*Re*	1.0	1.6
syn–*Si*	0.0	0.0
syn–*Re*	0.1	−0.2
anti–*Si*	1.6	1.6

Table S11. Total energy difference, $\Delta\Delta E^{\ddagger}$, for the DABCO–catalyzed Cloke–Wilson rearrangement of vinylcyclopropane **1d** stereoisomeric transition state structures associated with the 5–*exo*–*trig* cyclization of intermediate **int1d–C$_\beta$** via **TS2d–C$_\beta$** (w.r.t. *syn*–*Si*), using different setups: the main text basis set (6-31+G(d,p)) and a bigger one (6-311++G(3df,3pd)) with the hybrid meta–GGA functional M06–2X, and two different functionals, the hybrid GGA functional with Grimme's dispersion correction B3LYP–D3(BJ) and range–separated hybrid GGA functional with Grimme's dispersion correction ωB97X–D, in conjunction with the main text basis set (6-31+G(d,p)). All quantities in kcal mol^{-1}. In all cases the geometries were the SMD(DMSO)–M06–2X/6-31G+(d,p) optimized ones, and the implicit solvent was introduced by means of SMD(DMSO) correction.

TS2d–C$_\beta$	M06–2X 6-31+G(d,p)	M06–2X 6-311++G(3df,3pd)	B3LYP–D3(BJ) 6-31+G(d,p)	ωB97X–D 6-31+G(d,p)
anti–*Re*	1.6	1.3	1.4	1.5
syn–*Si*	0.0	0.0	0.0	0.0
syn–*Re*	−0.2	−0.1	−0.2	−0.3
anti–*Si*	1.6	1.3	1.4	1.5

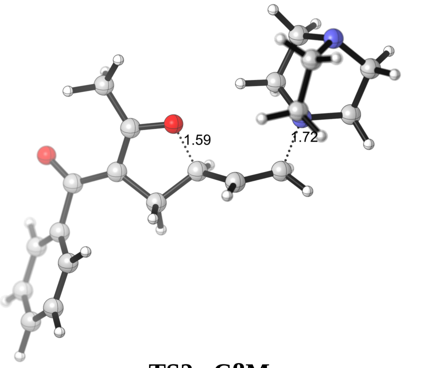

TS2e-CβMe-syn
$\Delta\Delta G^{\ddagger}$ = 0.0 kcal/mol

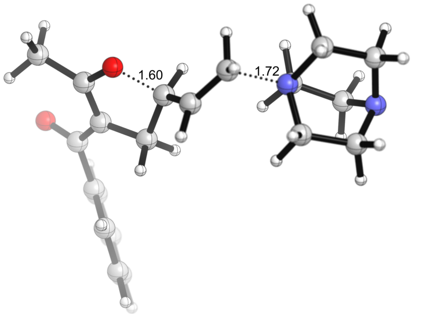

TS2e-CβMe-anti
$\Delta\Delta G^{\ddagger}$ = 1.1 kcal/mol

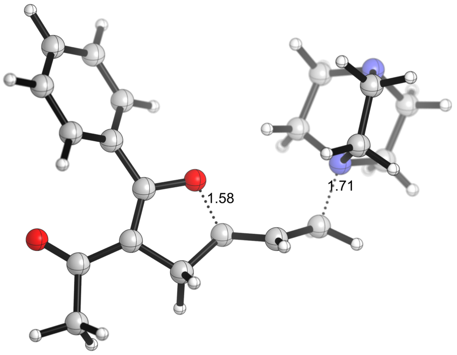

TS2e-CβPh-syn
$\Delta\Delta G^{\ddagger}$ = 0.1 kcal/mol

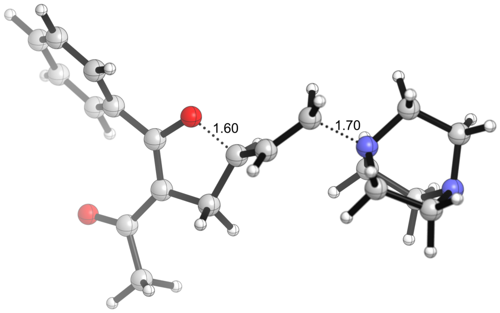

TS2e-CβPh-anti
$\Delta\Delta G^{\ddagger}$ = 3.2 kcal/mol

Figure S8. Computed SMD(DMSO)–M06–2X/6-31+G(d,p) regioisomeric transition state structures for the DABCO–catalyzed Cloke–Wilson rearrangement of vinylcyclopropane **1e**, 5–*exo*–*trig* cyclization of intermediate **int1e–C$_\beta$** via **TS2e–C$_\beta$Me** and **TS2e–C$_\beta$Ph**. Key bond lengths are given in Angstroms (Å). Relative free energy differences ($\Delta\Delta G^{\ddagger}$ values at 298 K) are also displayed in the figure.

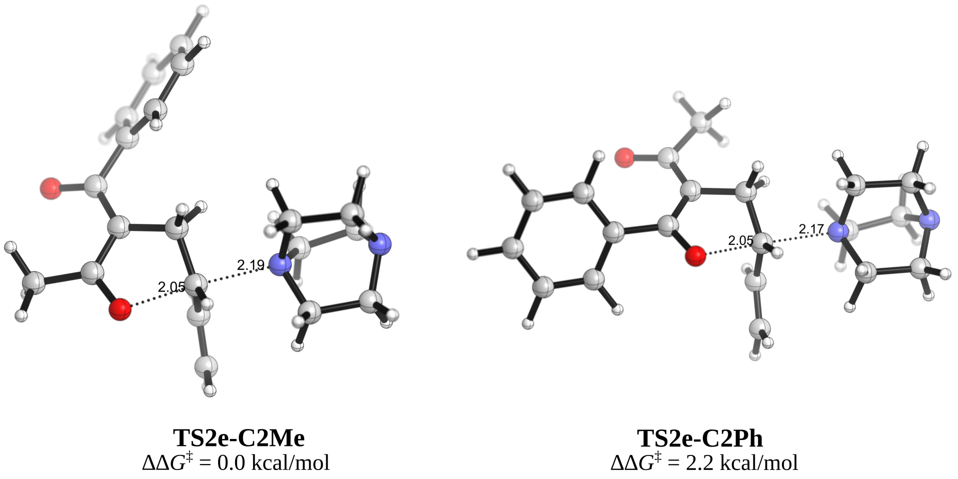

Figure S9. Computed SMD(DMSO)–M06–2X/6-31+G(d,p) regioisomeric transition state structures for the DABCO–catalyzed Cloke–Wilson rearrangement of vinylcyclopropane **1e**, 5–*exo*–*tet* cyclization of intermediate **int1e–C**$_2$ via **TS2e–C**$_2$**Me** and **TS2e–C**$_2$**Ph**. Key bond lengths are given in Angstroms (Å). Relative free energy differences ($\Delta\Delta G^{\ddagger}$ values at 298 K) are also displayed in the figure.

Table S12. Total energy difference, $\Delta\Delta E^{\ddagger}$, for the DABCO–catalyzed Cloke–Wilson rearrangement of vinylcyclopropane **1e** stereoisomeric transition state structures associated with the 5–*exo*–*trig* cyclization of intermediate **int1e–C**$_\beta$ (w.r.t. **TS2e–C**$_\beta$**Me–*syn***), using different setups: the main text basis set (6-31+G(d,p)) and a bigger one (6-311++G(3df,3pd)) with the hybrid meta–GGA functional M06–2X, and two different functionals, the hybrid GGA functional with Grimme's dispersion correction B3LYP–D3(BJ) and range–separated hybrid GGA functional with Grimme's dispersion correction ωB97X–D, in conjunction with the main text basis set (6-31+G(d,p)). All quantities in kcal mol^{-1}. In all cases the geometries were the SMD(DMSO)–M06–2X/6-31G+(d,p) optimized ones, and the implicit solvent was introduced by means of SMD(DMSO) correction.

TS2e–C$_\beta$	M06–2X 6-31+G(d,p)	M06–2X 6-311++G(3df,3pd)	B3LYP–D3(BJ) 6-31+G(d,p)	ωB97X–D 6-31+G(d,p)
Ph–*syn*	0.9	0.8	0.9	0.8
Me–*syn*	0.0	0.0	0.0	0.0
Ph–*anti*	3.2	2.8	3.1	3.0
Me–*anti*	1.4	1.2	1.1	1.2

Table S13. Total energy difference, $\Delta\Delta E^{\ddagger}$, for the DABCO–catalyzed Cloke–Wilson rearrangement of vinylcyclopropane **1e** stereoisomeric transition state structures associated with the 5–*exo*–*tet* cyclization of intermediate **int1e–C$_2$** (w.r.t. **TS2e–C$_2$Me**), using different setups: the main text basis set (6-31+G(d,p)) and a bigger one (6-311++G(3df,3pd)) with the hybrid meta–GGA functional M06–2X, and two different functionals, the hybrid GGA functional with Grimme's dispersion correction B3LYP–D3(BJ) and range–separated hybrid GGA functional with Grimme's dispersion correction ωB97X–D, in conjunction with the main text basis set (6-31+G(d,p)). All quantities in kcal mol^{-1}. In all cases the geometries were the SMD(DMSO)–M06–2X/6-31G+(d,p) optimized ones, and the implicit solvent was introduced by means of SMD(DMSO) correction.

TS	M06–2X 6-31+G(d,p)	M06–2X 6-311++G(3df,3pd)	B3LYP–D3(BJ) 6-31+G(d,p)	ωB97X–D 6-31+G(d,p)
TS2e–C$_2$Ph	2.0	1.9	2.3	2.3
TS2e–C$_2$Me	0.0	0.0	0.0	0.0

CARTESIAN COORDINATES (IN ANGSTROMS), TOTAL AND FREE ENERGIES (IN HARTREE, AT 298 K)

```
DABCO(Toluene)
E(RM062X) =  -345.202995326
Sum of electronic and thermal Free Energies =  -345.048639
Charge =  0 Multiplicity = 1

C       0.0061661387      -0.0054344454      -0.0010286481
C      -0.0075872926       0.0036517574       1.5561903886
H       1.0286809682      -0.0178214496      -0.3931691172
H      -0.4958751417       0.8779392439      -0.4102279943
H       1.0011079273      -0.1182232865       1.9655526555
H      -0.4139942396       0.94204547         1.9482715595
C      -0.0750144368      -2.3916317522       0.0803763513
H      -0.5491743231      -3.274648502       -0.3611213817
H       0.9803030588      -2.3996749813      -0.2130406023
C      -0.2339901348      -2.3732400979       1.629424666
H      -0.8844498455      -3.1843738243       1.9741235974
H       0.7319768492      -2.4852594096       2.1332267134
C      -2.1773081761      -0.99236487         1.4748001516
H      -2.8089978356      -1.7704768517       1.916321503
H      -2.5918196386      -0.0217929926       1.7681162367
C      -2.0989718698      -1.1320260005      -0.0742204207
H      -2.59618093        -2.0451828924      -0.4188965659
H      -2.575066624       -0.284152084       -0.578116451
N      -0.8336479953      -1.1025130807       2.0582371227
N      -0.6953243699      -1.1945714887      -0.5030729717
```

1aPh
E(RM062X) = -654.010895362
Sum of electronic and thermal Free Energies = -653.814715
Charge = 0 Multiplicity = 1

C	0.0028145555	-0.0085129596	-0.0874826684
C	0.1406628661	-0.0912989078	1.4477487563
C	1.3517212493	-0.0662630475	0.5857794794
H	-0.1022820986	0.8427842153	1.9478958208
H	1.9629490104	0.8290906521	0.5853069857
H	1.8907833433	-0.9917041251	0.4118802456
C	-0.4658082068	1.2664566419	-0.7153551564
O	-1.004707609	1.2388220999	-1.8074276634
C	-0.4810425011	-1.2775860165	-0.7585689965
O	0.2941352816	-2.1691471891	-1.0371014865
C	-0.3533855141	-1.2947048583	2.1852879023
C	0.2737027428	-2.5430048662	2.1191346456
C	-1.507623993	-1.1633175631	2.9686120767
C	-0.2468509284	-3.6334145518	2.8149316576
H	1.1690624337	-2.6735656459	1.5202457649
C	-2.0258821219	-2.2504009331	3.6668233069
H	-2.0010321803	-0.195987205	3.0312449827
C	-1.396300469	-3.4926792971	3.5897054424
H	0.2521457331	-4.5954842008	2.7502783514
H	-2.9199529233	-2.1276102256	4.2705514249
H	-1.7971066942	-4.3433156636	4.132146391
C	-0.2794235143	2.5737673807	0.0195003399
H	0.6475051243	2.6202699143	0.5956626442
H	-0.3064504759	3.3884536693	-0.7047003543
H	-1.1148946998	2.7040788619	0.7169670431
C	-1.9633894713	-1.4224382708	-0.9947897149
H	-2.1701528932	-1.2543792711	-2.0555930608
H	-2.2559236348	-2.4436067981	-0.738136907
H	-2.5472205235	-0.7061648917	-0.4131461582

TS1aPh-C2
E(RM062X) = -999.185416446
Sum of electronic and thermal Free Energies = -998.809354
Charge = 0 Multiplicity = 1

C	0.0659117429	-0.1890406328	0.0245370168
C	0.0135510573	-0.0740585164	2.120783636
C	1.0987836727	-0.2002000929	1.1332506268
H	-0.4827186532	0.892257658	2.1655198828
C	-0.3220726666	-1.4890535204	-0.4961429303
O	0.2241209988	-2.5244047838	-0.0877149275
C	-0.3965112845	1.0688142295	-0.5238515445
O	-1.2786858163	1.1752572808	-1.3836994263
C	0.2895182662	2.3525680754	-0.0567504764
H	0.3323073464	2.4599524293	1.032272241
H	1.3211529675	2.3778069162	-0.4267272543
H	-0.254436743	3.2001208757	-0.4753060913
H	1.6277853015	-1.1524347945	1.1641778236
H	1.7961273683	0.6381456017	1.1179418298
C	2.8849414952	-0.0294137619	5.439203383
C	2.21120941	-0.3805488403	4.0794532511
H	3.9529797616	0.1642444242	5.3042448903
H	2.7796007761	-0.8520827747	6.1528459976
H	2.8702485558	-0.1586613872	3.234727165
H	1.9258229797	-1.4356371104	4.0263858035
C	2.2665381399	2.2377533339	5.0300418436
H	1.9105867632	3.1578217963	5.5024372133
H	3.3014075681	2.4005055761	4.714129259
C	1.3678639234	1.855803436	3.8170091148
H	0.4428209474	2.4421893552	3.799530057
H	1.8869511764	2.0076055064	2.8653208018
C	0.1359539565	0.2531066242	5.1121580186
H	-0.8202976474	0.7528811527	4.9265988335
H	-0.0614205342	-0.817761856	5.2201955678
C	0.8651788545	0.8402773524	6.3559020157
H	0.3789004429	1.7586787833	6.6989850026
H	0.8551252614	0.1256061792	7.1837829315
N	2.2566557893	1.1600731185	6.0229642298
N	0.9928611056	0.4341491761	3.9253886533
C	-1.3998866204	-1.6045256075	-1.5506326429
H	-1.1242637928	-1.0539408529	-2.4536406865
H	-1.5401532403	-2.6632512305	-1.7761432504
H	-2.3350274028	-1.1598203208	-1.1998140032
C	-0.7996565236	-1.2012364692	2.605608376
C	-0.2850494692	-2.499163187	2.7248113235
C	-2.1110028713	-0.9465553444	3.0350992585
C	-1.0641130158	-3.5126894297	3.2794544065
H	0.7120278353	-2.7218218004	2.3633501129
C	-2.8903256132	-1.9603338586	3.5802925034
H	-2.5194109325	0.0555198006	2.9258929401
C	-2.3630816507	-3.2468961653	3.7098087929
H	-0.6583974623	-4.5160989479	3.3619462002
H	-3.9067474216	-1.7514904347	3.8990553685
H	-2.9683493925	-4.0420206114	4.1345012811

TS1aPh-C3
E(RM062X) = -999.175415377
Sum of electronic and thermal Free Energies = -998.799382
Charge = 0 Multiplicity = 1

C	-0.0516564702	-0.082246225	0.0244790819
C	-0.0490175562	-0.1638159873	2.0074944576
C	1.0745003706	-0.1254273258	1.0708274158
H	-0.5677224923	0.7699611466	2.1902061476
C	-0.6304204326	-1.3401536422	-0.4370938785
O	-0.4792481189	-2.3892861467	0.2008663296
C	-0.4567752277	1.2054427585	-0.5194377811
O	-1.360786744	1.3417048247	-1.3480957101
C	0.253123872	2.4702866654	-0.0424506591
H	1.3002449249	2.4751508514	-0.3644987353
H	-0.2565077384	3.3252021516	-0.4875212876
H	0.2416448623	2.5819003083	1.0481791069
H	1.5736472446	0.8421331696	1.0941289938
C	1.0568068641	-1.3217786169	6.0707855429
C	0.8160688469	-1.4729180201	4.5408167877
H	1.9712156618	-1.8420749329	6.3698126245
H	0.2269856892	-1.7429935477	6.6464069995
H	1.7007101344	-1.8661352283	4.0354757017
H	-0.0296530812	-2.1320020305	4.3177429375
C	2.1985526757	0.7157951337	5.5707807789
H	2.3684019646	1.7406907265	5.9131846709
H	3.1321899646	0.159951896	5.69809325
C	1.7411060852	0.6956456881	4.0826824103
H	1.4856708868	1.6981479553	3.7207331557
H	2.5096722374	0.2770387709	3.4253723958
C	-0.5704292319	0.4846483341	4.7201328738
H	-0.8502515966	1.4137624898	4.2126314429
H	-1.4264519671	-0.1968161614	4.6778870783
C	-0.106941186	0.7516929389	6.1799907612
H	0.0166152068	1.8236344456	6.3620372221
H	-0.8398097091	0.3714704842	6.8973224808
N	1.1802037	0.0959944118	6.4255394939
N	0.5335686907	-0.1420848043	3.966440931
H	-0.6050901554	-1.0898896236	2.1160296938
C	-1.4281726667	-1.3927018263	-1.7227104126
H	-2.4140487223	-0.9448232789	-1.5714435015
H	-0.952053212	-0.8246224381	-2.5242739022
H	-1.5371589129	-2.4430717066	-1.9996591452
C	2.1429599174	-1.1896276119	0.9876329218
C	2.0203612995	-2.4792794421	1.5121843193
C	3.3451756527	-0.8365249899	0.3633314056
C	3.0757971838	-3.3854073459	1.4211501395
H	1.0846595205	-2.7852286715	1.9642276589
C	4.3995795793	-1.7419862362	0.2618349499
H	3.454279351	0.1635915992	-0.0515555602
C	4.2682764138	-3.0224879418	0.7959310063
H	2.9588451148	-4.3850073662	1.8294781454
H	5.3209339675	-1.4454065833	-0.2304083117
H	5.0858335631	-3.7332329519	0.722216607

int1aPh-C2
E(RM062X) = -999.215770547
Sum of electronic and thermal Free Energies = -998.834137
Charge = 0 Multiplicity = 1

C	0.0157167219	-0.0329281553	0.0219605438
C	0.0508863571	-0.1201810947	2.5128361644
C	0.9163081772	-0.1140537677	1.2369557811
H	-0.7818855377	0.5647871941	2.3409582909
C	-0.6195014218	1.2333803139	-0.1385146629
O	-0.4333897938	2.1611852536	0.695465535
C	-0.1525187615	-1.126940104	-0.8875006159
O	-0.8983608147	-1.1164268314	-1.8845934274
C	-1.538752902	1.518351019	-1.3085586381
H	-1.8684177182	2.5573501736	-1.2353209573
H	-1.0327456134	1.3414086755	-2.261011162
H	-2.399411309	0.8437648947	-1.3031115004
C	0.6403421532	-2.4130653587	-0.6397542303
H	0.3529936693	-2.8835125524	0.3067698984
H	0.4217354621	-3.1031193177	-1.4560963559
H	1.7202708689	-2.2294187098	-0.6105082057
H	1.5510645314	-1.0032795228	1.2167387618
H	1.5728507597	0.760989453	1.2403782257
C	1.4498845558	2.7035090333	4.7262634578
C	1.1002660453	1.9735370671	3.4137455324
H	0.6115659916	3.3100576906	5.0799740073
H	2.2979379335	3.3715963197	4.5562554856
H	0.2665700034	2.4020797013	2.8545249417
H	1.9619176505	1.9376174175	2.7488326088
C	0.5595696116	1.0476062023	6.1637539114
H	0.8242306586	0.2064273663	6.8107316198
H	-0.0711835844	1.7294680983	6.739534439
C	-0.2060119775	0.5568655838	4.9136744753
H	-0.599119606	-0.4504883582	5.0470089691
H	-1.0252765341	1.2231269926	4.630043118
C	2.0031217405	-0.1582185519	4.1798742488
H	1.7060641507	-1.1125736888	4.6171493294
H	2.612105679	-0.3406804133	3.2921615096
C	2.7176152159	0.7482111267	5.2147209182
H	3.119723915	0.1316494779	6.022591268
H	3.5513160051	1.283957743	4.7528327551
N	0.7435743852	0.5344657395	3.7405051678
N	1.7898040813	1.7346480579	5.7722844236
C	-0.5018476988	-1.4930871541	2.8397131012
C	-1.8857887889	-1.6542263012	2.9606610642
C	0.3159264667	-2.6222879069	2.9717770641
C	-2.4420697574	-2.9044365911	3.2262283808
H	-2.533741061	-0.7912599789	2.8276897557
C	-0.234441359	-3.8710787083	3.2531556422
H	1.3901861829	-2.540667856	2.8282705287
C	-1.6154440398	-4.0150068408	3.3830915718
H	-3.519776573	-3.0102166336	3.3016868876
H	0.4150451992	-4.7356930263	3.3502796162
H	-2.044530148	-4.9907737067	3.5884982567

int1aPh-C3
E(RM062X) = -999.212788303
Sum of electronic and thermal Free Energies = -998.831699
Charge = 0 Multiplicity = 1

C	-0.0334078138	0.0086926592	0.1817563267
C	0.1274020528	0.6265983374	2.6317023266
C	0.9101313052	0.4077671583	1.3201244503
H	-0.0850643857	1.6801457801	2.8115060722
C	-1.2446965895	0.7548722587	0.0532096806
O	-1.5356787813	1.6797361765	0.8537355486
C	0.3414902125	-1.0309401266	-0.7315209746
O	-0.3620817212	-1.4470598204	-1.6709262016
C	-2.2361124889	0.461841698	-1.0552739383
H	-3.0650427881	1.1663418382	-0.9562844338
H	-1.765154242	0.5585150703	-2.036992151
H	-2.5985587429	-0.5679611454	-1.0021779099
C	1.7109283954	-1.7012958333	-0.5677744838
H	1.7915097705	-2.2490385436	0.3793832197
H	1.8308724285	-2.4128554022	-1.3858151367
H	2.5276953215	-0.9731988363	-0.6075469317
H	1.5802314233	-0.4412799875	1.4835409837
C	2.7452560361	0.3531164964	5.4412374686
C	2.2290436557	0.6726563658	4.0174521796
H	2.7263497962	1.2465220361	6.0712548762
H	3.777934968	0.0002207336	5.3893163745
H	2.1866237908	1.7436282722	3.8059886211
H	2.8281881859	0.1910472258	3.243039907
C	0.5934864801	-0.0988403024	6.34769554
H	-0.0712807863	-0.9039335724	6.6718975855
H	0.6736855879	0.6218295185	7.165054679
C	0.0399198432	0.599347165	5.0840139992
H	-1.0088582063	0.3640268026	4.8921235905
H	0.1544896848	1.6852905928	5.1190460361
C	0.8801415291	-1.3738435835	3.9372494935
H	-0.1555079129	-1.7093854926	4.032971919
H	1.2755011815	-1.735977003	2.987770236
C	1.754509869	-1.791407613	5.1431880753
H	1.2901841324	-2.6334639083	5.6621123785
H	2.7484112199	-2.1051946705	4.8129496816
N	0.8307862935	0.1304999928	3.8883909295
N	1.9124241741	-0.6717964067	6.0733258707
H	-0.8198933075	0.0857930987	2.6098640142
C	1.8202381007	1.588356529	0.983474176
C	3.1941186122	1.3903701744	0.8049501452
C	1.3120950297	2.8859921773	0.8247135012
C	4.0437441033	2.4485643555	0.4797523455
H	3.6105992393	0.3908561489	0.9198735255
C	2.1588520731	3.9450585517	0.5033811567
H	0.2427377883	3.0451903086	0.9332852247
C	3.5278465263	3.7337898547	0.330917925
H	5.1055903801	2.2657385193	0.3416478438
H	1.7444055185	4.941378295	0.3782692589
H	4.1831125402	4.5614754277	0.0768062168

TS2aPh-C2
E(RM062X) = -999.181153306
Sum of electronic and thermal Free Energies = -998.804714
Charge = 0 Multiplicity = 1

C	0.0101473353	0.0135118422	-0.0050330962
C	-0.0027487851	-0.0641888826	2.4890119403
C	0.863024781	-0.0146196772	1.2504516359
H	-0.1218644905	0.8148597903	3.1074666276
C	-1.2421153195	0.5916700109	0.2229863885
O	-1.5351906807	0.9237315934	1.4182463778
C	0.4992596268	-0.4233824596	-1.280288849
O	-0.1552038602	-0.3909375155	-2.3334815661
C	-2.26785791	0.8283811523	-0.8532517255
H	-3.123503539	1.3498581302	-0.4203640176
H	-1.8406256252	1.4033135552	-1.6780541878
H	-2.5874848641	-0.126129744	-1.2815281831
C	1.9230377447	-0.9715788547	-1.331774896
H	1.9985502922	-1.913278418	-0.7738872377
H	2.1885972446	-1.160494755	-2.3728146347
H	2.6429652632	-0.2708186197	-0.894616595
H	1.524833773	-0.8884665108	1.2465480702
H	1.5060903613	0.8727040154	1.3167011871
C	3.6707032562	0.1064752646	4.8166393291
C	2.6882116063	0.253532583	3.6198181207
H	3.679801898	1.0121529733	5.4306282514
H	4.6891738711	-0.0654911115	4.4571523612
H	2.3654740151	1.290818686	3.4831542729
H	3.1461276225	-0.0888353031	2.6882670809
C	1.9827377159	-0.6844259708	6.2893685931
H	1.6250292572	-1.5694066707	6.8239958075
H	2.1378393526	0.1117138261	7.0230568177
C	0.9586511465	-0.2411831033	5.2063011656
H	-0.0004653722	-0.7488432007	5.3335756699
H	0.7858672177	0.8400960048	5.2297522036
C	1.8793822865	-2.0019232152	3.860876708
H	1.0099776751	-2.5837885096	4.1788384871
H	2.1250369033	-2.293251999	2.8344047861
C	3.0868634603	-2.2009940052	4.8222950106
H	2.9249626023	-3.0729714766	5.4622470082
H	4.0131611234	-2.3622504058	4.2626253031
N	1.4889765617	-0.5772992071	3.8675079422
N	3.2667203358	-1.0158619172	5.6664081729
C	-0.8993798098	-1.2121053448	2.7448753908
C	-1.9165053644	-1.0994794169	3.7015929961
C	-0.763075686	-2.4113515844	2.036249848
C	-2.7617881215	-2.1716192524	3.967423155
H	-2.0580191157	-0.1522807036	4.2155238458
C	-1.6122824821	-3.485055224	2.2994044386
H	0.0005730958	-2.5077554544	1.2706731933
C	-2.6071622712	-3.3704942848	3.2686336148
H	-3.5493901106	-2.0690845175	4.7075112301
H	-1.5001986182	-4.4086070729	1.7400565395
H	-3.2694740835	-4.206967964	3.4697894356

TS2aPh-C3
E(RM062X) = -999.183103708 A.U. after 2 cycles
Sum of electronic and thermal Free Energies = -998.804972
Charge = 0 Multiplicity = 1

C	0.1534340759	0.1534940302	0.1273243945
C	0.2348972462	0.3397033258	2.5655836918
C	1.0866079091	0.3544923715	1.3132801014
H	0.1672825333	1.1942940485	3.2264922783
C	-1.1083873155	0.6640056678	0.4050180552
O	-1.3017462851	1.0814312605	1.6063741775
C	0.6908262057	-0.4606499642	-1.0577574736
O	1.8711521754	-0.8403723816	-1.0940204507
C	-2.2831163186	0.7772534082	-0.5342356088
H	-3.0054327163	1.4683483666	-0.0966125036
H	-1.9922094739	1.1368015159	-1.5220152896
H	-2.7712503556	-0.1958079806	-0.6542493641
C	-0.1680212759	-0.6728680213	-2.2927205386
H	-0.3423715154	0.2803110836	-2.8031063833
H	0.3746624579	-1.3338385022	-2.9703998626
H	-1.1400049806	-1.1120387661	-2.0559122133
H	1.7805110574	-0.4910419349	1.3270291889
C	3.6414728321	-0.3723082427	5.1323286094
C	2.8560832889	0.1527698997	3.8968338553
H	3.7356841381	0.4047315056	5.8970559003
H	4.6497264218	-0.6829190583	4.8443524694
H	2.7317664235	1.2400000676	3.9149321847
H	3.3541761472	-0.1115211111	2.9599464323
C	1.667255388	-1.0499795968	6.28077406
H	1.102589825	-1.9250040565	6.6170100793
H	1.8648395504	-0.4218747189	7.154037575
C	0.8789076627	-0.2588731198	5.2011602231
H	-0.1650993645	-0.5828923074	5.1337377875
H	0.8884523139	0.8176298952	5.4004490337
C	1.6551816599	-1.9228166962	3.6528603491
H	0.6627282302	-2.3709618609	3.7677437994
H	1.9843933262	-2.0838254659	2.6222077418
C	2.6695892039	-2.5012849683	4.6792475094
H	2.2770603029	-3.4125559467	5.1395759624
H	3.6173879807	-2.7517093172	4.1935319346
N	1.514677715	-0.4700614631	3.8836751811
N	2.9436373863	-1.5157977545	5.7300157477
C	1.9064113063	1.6280908261	1.1577036284
C	3.1989897722	1.5576227854	0.6278208127
C	1.384075454	2.8855015377	1.4862017114
C	3.9621861772	2.7107798585	0.4498712419
H	3.5935091526	0.5875902679	0.3362377452
C	2.146666785	4.0395248044	1.3098942224
H	0.3686951736	2.9625347099	1.8667791144
C	3.440991116	3.9568426704	0.7966555644
H	4.9640713912	2.6349434132	0.037036785
H	1.7258986638	5.0064631886	1.5708499397
H	4.0345614375	4.8559857942	0.6610940116
H	-0.4070969619	-0.5120903494	2.7587820359

prod1aPh-C2
E(RM062X) = -654.021877943
Sum of electronic and thermal Free Energies = -653.824296
Charge = 0 Multiplicity = 1

C	-0.0917017277	0.040396556	-0.3354043089
C	-0.035878719	-0.4460244284	1.9899801397
C	0.792762052	-0.6322978628	0.6941668949
H	0.5606517082	-0.0165927605	2.7983019553
C	-1.0821000351	0.6959184635	0.3143533955
O	-1.049557652	0.5318868011	1.6566527923
C	0.1391178072	0.0278854535	-1.7831936054
O	-0.602111549	0.580388321	-2.5881478188
C	-2.1847843417	1.566197846	-0.1650607894
H	-2.1449773878	2.523476552	0.3642474971
H	-2.1080006915	1.7233817235	-1.2385511253
H	-3.147399074	1.100124676	0.0717604479
C	1.3631284769	-0.7255449613	-2.253913277
H	1.2661852058	-1.7889057014	-2.0078899674
H	1.4699156936	-0.6108518626	-3.3328519972
H	2.262860638	-0.3562456644	-1.750120261
H	0.9657611735	-1.6937577834	0.492641369
H	1.7707577344	-0.1430641088	0.7723090008
C	-0.6882949637	-1.7240989588	2.4658067924
C	-1.9390880662	-2.1252784882	1.9920405078
C	0.0048779221	-2.5520122813	3.3526520657
C	-2.4857820107	-3.3420636167	2.3980654998
H	-2.4912110705	-1.4817244241	1.3133160861
C	-0.5398722442	-3.7690398033	3.7568974474
H	0.9755581634	-2.2398591351	3.7317574227
C	-1.7882191355	-4.1671510864	3.2791316691
H	-3.4606608762	-3.6437648823	2.0271481691
H	0.0057521439	-4.4015492816	4.450589646
H	-2.2176869741	-5.1122360687	3.5968348075

prod1aPh-C3
E(RM062X) = -654.020176433
Sum of electronic and thermal Free Energies = -653.821028
Charge = 0 Multiplicity = 1

C	-0.0209428202	-0.0495279671	-0.0049930729
C	0.0370434225	0.0484376668	2.3461508237
C	0.9900954239	-0.0450615425	1.133123127
H	0.3506679881	0.7688950868	3.1023840324
C	-1.236218809	0.2886065828	0.4920882331
O	-1.2416954325	0.4745102026	1.8291412471
C	0.2828711947	-0.2390407756	-1.4264923899
O	-0.5953661871	-0.369183103	-2.2729248413
C	-2.5445761137	0.5242584705	-0.1697819114
H	-3.0618448836	1.3458346421	0.3321860688
H	-2.4071351703	0.7375552715	-1.2284733391
H	-3.1668300986	-0.373006022	-0.0810704674
C	1.7411475456	-0.2747022998	-1.8265646832
H	2.1630822066	-1.2563663769	-1.5824594224
H	1.8191877736	-0.1136913523	-2.9026043511
H	2.3294416848	0.4748975169	-1.2882066119
H	1.6206236302	0.8527651045	1.0772045381
H	-0.1062642186	-0.9320398577	2.8123193602
C	1.9043207026	-1.2516809843	1.2141578356
C	3.2696349083	-1.0893120094	1.4566199911
C	1.3976385145	-2.546312399	1.0516426086
C	4.117115086	-2.1951599571	1.535813356
H	3.6743343451	-0.0868184086	1.576430371
C	2.2403545823	-3.6523143105	1.1286136832
H	0.3378961979	-2.6855780508	0.8487595399
C	3.604374915	-3.4798619914	1.3705640325
H	5.1770994056	-2.0505958258	1.7221567234
H	1.8339508828	-4.6502143124	0.9935037918
H	4.2618042545	-4.3419853443	1.4265403205

TS1aMe-C2
E(RM062X) = -807.504028882
Sum of electronic and thermal Free Energies = -807.177280
Charge = 0 Multiplicity = 1

C	-0.1306143465	-0.0288476224	0.0492810714
C	-0.0302087872	-0.0378696893	2.1153260317
C	0.9945439508	-0.0425645919	1.068957359
H	-0.576544444	0.8893232358	2.266190146
C	-0.5287711657	-1.31959425	-0.4821567314
O	-0.0390177067	-2.3676904157	-0.0360420398
C	-0.6516611802	1.2360418502	-0.4294360562
O	-1.5800108466	1.3453204944	-1.2377208194
C	0.0096760979	2.5228330395	0.0620469548
H	1.0247780252	2.6014281604	-0.344081148
H	-0.580118294	3.3664200979	-0.2981695628
H	0.0894752099	2.5848062196	1.1529360621
H	1.5679075036	-0.9690221451	1.0215475345
H	1.6315817766	0.8390382996	1.0269480801
C	2.4916177834	-0.2321944981	5.7858899532
C	1.609846351	-0.7242997147	4.60098738
H	3.5561117978	-0.3415125581	5.5567541428
H	2.2822239041	-0.8097087277	6.6909028247
H	2.1919251463	-1.2797771153	3.8582461701
H	0.7982940988	-1.3693056635	4.9494914058
C	2.6846487273	1.9738949748	4.9062427908
H	2.3790517151	3.0128391474	5.0646888021
H	3.7774284266	1.9482011075	4.8670136354
C	2.0725143826	1.4027421902	3.5952046524
H	1.6427317737	2.1922106528	2.9680118261
H	2.8225059155	0.8683403591	3.0049304438
C	0.0621678405	1.1001039245	4.8620489099
H	-0.2762270779	2.0272731121	4.386664303
H	-0.8089384745	0.4523792358	4.9995664013
C	0.7847817606	1.378583114	6.2114626476
H	0.596933345	2.4017542133	6.5498143429
H	0.4340958218	0.6965461767	6.9920473309
N	2.2298139519	1.1859064636	6.0557561984
N	1.0041735541	0.4441225464	3.9353012925
C	-0.6789232287	-1.2984373386	2.6108965328
H	0.0513863836	-2.1036278671	2.7096483227
H	-1.1661363399	-1.1177536544	3.5717884324
H	-1.4452321654	-1.6458045948	1.9155415251
C	-1.5560520504	-1.414572781	-1.588773228
H	-1.2762184195	-0.7986460424	-2.446622069
H	-1.6432544077	-2.4634487474	-1.878206844
H	-2.5227570868	-1.0326029412	-1.2483979029

TS1aMe-C3
E(RM062X) = -807.503219667
Sum of electronic and thermal Free Energies = -807.176546
Charge = 0 Multiplicity = 1

C	0.1037696474	-0.0072197051	-0.0395586787
C	0.0729202935	0.008289157	1.9216150218
C	1.2193385513	0.006907289	1.0114511223
H	-0.4501698983	0.943057945	2.0962460575
C	-0.4470109933	-1.2960659929	-0.4495948753
O	-0.2445628273	-2.325095801	0.2103570484
C	-0.3214596823	1.2481546679	-0.6422430086
O	-1.2136159941	1.3362225753	-1.4901704538
C	0.3635813913	2.5423177894	-0.2096706057
H	1.4096513431	2.5529641347	-0.5346298875
H	-0.1617037908	3.3719679525	-0.6836212342
H	0.354153015	2.6912251394	0.8758870986
H	1.7327816414	0.9677573943	0.9951556056
C	1.5031205461	-1.3068825099	5.7976832743
C	1.0245140257	-1.3716991873	4.317722328
H	2.5796160175	-1.489736188	5.8692010463
H	0.994277378	-2.0591573659	6.4072347523
H	1.8127928591	-1.7229067265	3.6475578869
H	0.1586152887	-2.029874965	4.1938128275
C	2.0160560137	1.0200351917	5.6349865932
H	1.7472778333	2.0082308393	6.0204967449
H	3.0761158769	0.8537954531	5.8470903297
C	1.7334093136	0.919360856	4.1068181239
H	1.4520106209	1.8872710183	3.6769113264
H	2.5998020018	0.5408613008	3.5558455943
C	-0.5454513666	0.4119325842	4.6788219728
H	-0.7868987453	1.4381053454	4.3815142378
H	-1.3925987289	-0.2283282348	4.4110708562
C	-0.1963733727	0.3188723957	6.1923897709
H	-0.4212778366	1.2591330903	6.7041024814
H	-0.7705332339	-0.4746785686	6.6797545776
N	1.2287540841	0.0188564437	6.3622012894
N	0.6216150554	-0.019797917	3.8890213796
H	-0.4743795677	-0.9207017608	2.0468174824
C	2.2408017236	-1.1208538453	1.0213842787
H	3.0087408485	-0.9240760617	1.7771905846
H	1.781315326	-2.0897532971	1.2117873336
H	2.7381736998	-1.167552832	0.0472179377
C	-1.2854703963	-1.4105737684	-1.7051667544
H	-2.266219692	-0.9539755442	-1.5467490439
H	-0.8354964905	-0.880891669	-2.5472226061
H	-1.4016289022	-2.4728077695	-1.9287492274

TS1aMe'-C2
E(RM062X) = -1190.86030245
Sum of electronic and thermal Free Energies = -1190.438381
Charge = 0 Multiplicity = 1

C	0.0671504064	-0.0963797528	-0.037511955
C	0.0559234133	-0.0963983415	1.9470348858
C	1.1713547099	-0.1590103468	1.0082319406
H	-0.4400836269	0.8571225549	2.0876370851
C	-0.4142676719	-1.3646145249	-0.5620942612
O	0.2148472603	-2.4182638355	-0.4064686702
C	-0.3252607313	1.1824476223	-0.5897183601
O	-1.0126502609	1.3343881106	-1.606525678
H	1.6948727589	-1.1138782488	0.9959655778
H	1.8493179276	0.6885698337	0.994593183
C	2.4488472586	-0.0405486394	5.7945928888
C	1.6423492168	-0.6285610592	4.6002484992
H	3.5254033666	-0.0980254608	5.6057057457
H	2.2334957019	-0.5911778355	6.7151533083
H	2.2883048948	-1.1803563943	3.9090800696
H	0.8506040753	-1.300353731	4.9461663843
C	2.5526861001	2.1414963018	4.8253715335
H	2.1742798024	3.164095958	4.9244033226
H	3.6460721303	2.1802153826	4.8269611419
C	2.0302097299	1.4777423117	3.5214281073
H	1.5822363577	2.2129068206	2.840534531
H	2.8377056164	0.9633074573	2.9919019603
C	-0.0083951018	1.1122510956	4.7203340638
H	-0.3771723831	1.9983681879	4.1910724263
H	-0.8442325542	0.4191636275	4.8580246955
C	0.6368779284	1.4889013284	6.0836265072
H	0.3771467776	2.5136837864	6.3654538899
H	0.2927927431	0.8179134373	6.8768653806
N	2.0993945334	1.3744281997	5.994808153
N	1.0069342529	0.4753238241	3.8646446774
C	-0.6333033665	-1.331964243	2.454767629
H	0.0637128946	-2.17062024	2.519257874
H	-1.0531497008	-1.1367567466	3.4434336642
H	-1.4676680618	-1.6255239382	1.8105606703
C	-1.76866713	-1.4551634497	-1.2105170721
C	-1.9522810843	-2.3321542606	-2.2826413628
C	-2.8687559328	-0.7687290275	-0.6856335986
C	-3.2165098007	-2.4991449223	-2.8472055673
H	-1.0976853455	-2.8760897087	-2.6750242865
C	-4.136837605	-0.9558516769	-1.2307940066
H	-2.7294982676	-0.0911392748	0.1534792967
C	-4.3115573786	-1.8144502317	-2.3188160417
H	-3.3483319966	-3.1683435843	-3.6924487702
H	-4.9891779493	-0.4299001615	-0.8106921033
H	-5.2990334828	-1.9516757875	-2.7498245924
C	0.1622955767	2.4402652551	0.1056639555
C	1.4418664988	2.9451664834	-0.144013148
C	-0.6973109789	3.1369147641	0.9604488479
C	1.8602507213	4.1297398508	0.462478156
H	2.1130074112	2.407615317	-0.8101337227
C	-0.2759536651	4.3178993186	1.5730880399
H	-1.6963852187	2.7504706844	1.151508697
C	1.0044594878	4.8149489289	1.326700393
H	2.8556789778	4.5153683328	0.2624759666
H	-0.9481431484	4.8485493754	2.2411971878
H	1.3329807724	5.7336047861	1.8036250142

TS1aMe'-C3
E(RM062X) = -1190.85863863
Sum of electronic and thermal Free Energies = -1190.436345
Charge = 0 Multiplicity = 1

C	0.0383513683	0.0024141544	0.0671724574
C	0.0216735418	-0.0479541362	1.9737875648
C	1.1808229154	-0.018495563	1.0902639421
H	-0.4770134531	0.8850071846	2.2156859394
C	-0.4619315559	-1.2626938734	-0.4607138667
O	-0.4108321892	-2.3255542001	0.1696854402
C	-0.5486712941	1.2577826749	-0.3785941839
O	-1.6799264225	1.3354594203	-0.8703865103
H	1.692717698	0.9422239197	1.1126694154
C	1.5837124955	-1.3630284828	5.8938383206
C	1.0442558695	-1.4475992718	4.4368491647
H	2.6664066934	-1.521252814	5.9182439645
H	1.1132118606	-2.1205570754	6.5276147995
H	1.8062905124	-1.8203226931	3.7471154349
H	0.1663751851	-2.0981754809	4.3673046961
C	2.0606587929	0.9726546504	5.6992308592
H	1.7693650176	1.9610788436	6.0678814468
H	3.1280863932	0.8355766235	5.8957500536
C	1.7576343322	0.8335961611	4.179772232
H	1.4786868538	1.7934212762	3.7307912579
H	2.6174172356	0.4332888824	3.6332877008
C	-0.5052983493	0.3456691294	4.81315182
H	-0.7337137932	1.3770139078	4.5246987345
H	-1.3682375662	-0.281866746	4.5664872941
C	-0.1297992289	0.245157166	6.3182425762
H	-0.3600728693	1.1792186874	6.8392134574
H	-0.681744395	-0.5640193797	6.8063179002
N	1.3064250279	-0.0348349915	6.4603672065
N	0.637638079	-0.1020945011	4.0013395918
H	-0.5096009381	-0.9826316957	2.1212365514
C	2.1724954223	-1.1656092189	1.0499337702
H	2.9212089686	-1.0329622984	1.836267497
H	1.6859004663	-2.1324328227	1.1846619802
H	2.6921515626	-1.1699376605	0.0854132883
C	-0.9859348024	-1.3204427694	-1.8739091311
C	-2.0140827646	-2.2191530036	-2.1719720645
C	-0.4085418857	-0.5719645199	-2.9046084869
C	-2.481705296	-2.3479414916	-3.4788545153
H	-2.4483065987	-2.8096735287	-1.3704534126
C	-0.8591154253	-0.7162916486	-4.2151522593
H	0.4008378835	0.119193559	-2.6825135257
C	-1.9026095807	-1.598078617	-4.5037150277
H	-3.2924695391	-3.0362687945	-3.6989555112
H	-0.3974377207	-0.1400846686	-5.0116083262
H	-2.2595450814	-1.7031597323	-5.5240991677
C	0.2203380166	2.5435279321	-0.1981134402
C	-0.4283902905	3.6495610388	0.3591743593
C	1.5372105305	2.6735878675	-0.6532303528
C	0.237997159	4.8681203281	0.4806235428
H	-1.4555488553	3.5470676782	0.6986363265
C	2.1949934509	3.8988163192	-0.5561766351
H	2.0428148342	1.8162321825	-1.0926670314
C	1.5491253298	4.9949099268	0.0188882818
H	-0.2664459589	5.719229197	0.9284996261
H	3.2114685008	3.9974582744	-0.9253406037
H	2.0660387208	5.9461835244	0.1044500891

TS1bPh-C2
E(RM062X) = -1113.69923269 A.U. after 2 cycles
Sum of electronic and thermal Free Energies = -1113.294363
Charge = 0 Multiplicity = 1

C	-0.0302716227	-0.0396455127	-0.0460258398
C	-0.0263020469	0.0161012996	2.0325324877
C	1.0408325906	0.0048736959	1.0215115803
H	-0.5877060931	0.9438846016	2.1046966462
C	-0.3327256253	-1.3286883755	-0.6308209836
O	0.2937739145	-2.3413804777	-0.2864921215
C	-0.6245317936	1.1933028245	-0.5063877098
O	-1.459954392	1.3455489861	-1.3879751344
C	-1.4292615255	-1.4631233046	-1.6656745515
H	-2.3759862142	-1.0679265708	-1.287578008
H	-1.1915564148	-0.884899225	-2.5623491557
H	-1.5305373932	-2.5206558957	-1.9149724592
H	1.6447593162	-0.9016376344	1.0028864359
H	1.6486271523	0.9083077243	0.996060156
C	2.9615579784	0.312331463	5.2392748578
C	2.2650320279	-0.0909326014	3.9060845852
H	3.9943162464	0.6243687738	5.0587812796
H	2.9816475482	-0.5265273899	5.9416929564
H	2.8547698222	0.2145391786	3.0368590377
H	2.0943495957	-1.1704266887	3.8445817298
C	2.0820485444	2.5024396471	4.8981958457
H	1.6587464724	3.3755629996	5.4034203772
H	3.0772549992	2.7751155291	4.5339659809
C	1.1661725206	2.0425834776	3.7267362078
H	0.1806113809	2.5175050681	3.7748543508
H	1.6018151062	2.2706769252	2.7489160836
C	0.1856196446	0.2941981948	5.0444930347
H	-0.8273752797	0.6880704698	4.9132078207
H	0.1121336556	-0.7935802413	5.1412760934
C	0.9052969728	0.9419290488	6.2641100378
H	0.3382577721	1.7960935693	6.6470937153
H	1.0141565176	0.2195071724	7.078228489
N	2.2361444964	1.4180973797	5.8732457288
N	0.9592664755	0.5861370128	3.8242054683
C	-0.7173319646	-1.1880347672	2.5224166933
C	-0.0940061141	-2.4418400092	2.5752142179
C	-2.0233992634	-1.0565199974	3.0179509286
C	-0.7614720961	-3.5331683201	3.1280781421
H	0.900338859	-2.5700551756	2.1638151199
C	-2.691045458	-2.1470902174	3.5634709814
H	-2.5160265872	-0.0885593354	2.9619996218
C	-2.055884998	-3.3892160827	3.6247992172
H	-0.2718028801	-4.5014398139	3.1581746972
H	-3.7043321831	-2.0325138573	3.9358321273
H	-2.5736123618	-4.2441404124	4.0491090384
C	-0.6580373998	3.5512674466	-0.2277855289
H	-0.4264406274	3.7130489916	-1.2852349395
H	-1.7477473473	3.5483459056	-0.1237176041
C	-0.015791779	4.6040447993	0.6491348043
H	1.0732993556	4.5821329614	0.5482766702
H	-0.368378369	5.5972448854	0.3565037402
H	-0.2723696132	4.4463882431	1.7010477099
O	-0.1391842576	2.2815951099	0.1775565493

TS1bPh-C3
E(RM062X) = -1113.68870294 A.U. after 2 cycles
Sum of electronic and thermal Free Energies = -1113.283435
Charge = 0 Multiplicity = 1

C	-0.0607394387	-0.0517905627	-0.0615695059
C	-0.0369678609	0.0376549173	1.9103680819
C	1.0793904183	0.0092430814	0.9675439861
H	-0.5650170242	0.9798547298	1.9981330077
C	-0.6361949036	-1.3313314986	-0.4388718638
O	-0.4434431007	-2.3447284202	0.2445456811
C	-0.4920653746	1.1910902545	-0.6757378065
O	-1.289374421	1.3319362712	-1.5916416383
C	-1.5013644218	-1.4480691451	-1.6769934857
H	-2.442502122	-0.9086837109	-1.5391990475
H	-1.0179914743	-1.0058987646	-2.5508438782
H	-1.7007610092	-2.5083348078	-1.8413119099
H	1.5539049998	0.9853631043	0.8869495762
C	-0.1371299914	1.3002016377	5.985762485
C	-0.5895223837	0.8607273846	4.564373884
H	-0.8072974454	0.8970810513	6.7504757671
H	-0.1393822091	2.39050291	6.0780713207
H	-1.3746236623	0.0981936446	4.5998014262
H	-0.9655592686	1.708958633	3.9830124515
C	1.2604950225	-0.6267634313	6.0321002637
H	2.2406245362	-1.0036790309	6.3378989091
H	0.5070694397	-1.0860446375	6.6793304085
C	0.9902880815	-0.9529813482	4.5339375968
H	1.892076606	-1.3120114916	4.0332750901
H	0.2054756644	-1.7068245702	4.4104988247
C	1.6763470278	1.2439395978	3.8455876394
H	2.4771510089	0.8336702902	3.2223563003
H	1.3167793624	2.1672576611	3.3774119498
C	2.144398755	1.4732095679	5.3125872338
H	3.1422866631	1.0540735956	5.4722181067
H	2.1870358267	2.5407682676	5.547449235
N	1.2225883532	0.8229531078	6.2506533169
N	0.5610121795	0.2801904965	3.8452566021
H	-0.5688679952	-0.885478585	2.1208443398
C	2.1504996294	-1.0539809372	0.9597005926
C	2.0455264931	-2.2895073663	1.6045726134
C	3.3382043487	-0.7548314562	0.2823542419
C	3.1038485948	-3.1959145869	1.5781959707
H	1.121413101	-2.5547597698	2.1046475523
C	4.3959837167	-1.6617369453	0.2454528264
H	3.4311788651	0.202871735	-0.2255574354
C	4.2823367481	-2.8873706521	0.8987969893
H	3.0011054529	-4.1533119449	2.0806416464
H	5.306440896	-1.4082676075	-0.2894194616
H	5.1028687577	-3.5982301396	0.8761566126
C	-0.3195844201	3.5576366089	-0.6149737179
H	-0.0431421439	3.6038741355	-1.6728319055
H	-1.4077447837	3.6546977265	-0.5505323892
C	0.3869886085	4.6229594614	0.1943174815
H	0.1182856419	5.6141984484	-0.1816972853
H	0.0989347111	4.5661668493	1.2484862921
H	1.4726527191	4.5104277776	0.1233814752
O	0.0730019362	2.2878183452	-0.0829698637

TS1bMe-C2
E(RM062X) = -922.018791956
Sum of electronic and thermal Free Energies = -921.661576
Charge = 0 Multiplicity = 1

C	-0.2268529847	-0.1632619907	0.0556713688
C	-0.2329789156	-0.0361620925	2.1013583823
C	0.8409787533	-0.2405758796	1.128772954
H	-0.6776217524	0.9537042886	2.1253789746
C	-0.7488689298	-1.4044161898	-0.4677786495
O	-0.4150600505	-2.49064976	0.0311498457
C	-0.5701001989	1.1294218932	-0.4967079487
O	-1.3354628636	1.3742017035	-1.4189978847
H	1.2900466881	-1.2330237484	1.1707917087
H	1.5754964337	0.5600293534	1.0566154477
C	2.7423370681	0.2015748372	5.3715843765
C	2.0268767692	-0.2352363967	4.0594969662
H	3.8068323633	0.3798159261	5.1934658306
H	2.6557999029	-0.5692716906	6.1436709318
H	2.6560622967	-0.0583294937	3.1821457718
H	1.7573014279	-1.2964310611	4.0750839993
C	2.1107063182	2.4340382826	4.8185902681
H	1.7864838261	3.3918364192	5.2361342098
H	3.1330918135	2.5587122609	4.4485074727
C	1.1567133964	1.9723883556	3.6805618917
H	0.2261831482	2.550320489	3.6755043279
H	1.6154762224	2.0699824911	2.6914897596
C	-0.0105430852	0.4574924784	5.1244357496
H	-0.9804417449	0.9328129007	4.9414396817
H	-0.1840489368	-0.6039579442	5.3251913637
C	0.7560529648	1.1415084877	6.2945574195
H	0.2797784187	2.0849700022	6.5790695324
H	0.7747331238	0.4956645064	7.1773488508
N	2.1353378895	1.4323739655	5.8903771546
N	0.7968593935	0.5586529971	3.8959633838
C	-0.9944855128	-1.1849051812	2.6899240235
H	-0.3110291399	-1.8712202252	3.2006390618
H	-1.750194927	-0.831919296	3.3941357101
H	-1.4836842485	-1.7721969389	1.9095731237
C	-1.7313863087	-1.403590668	-1.6197907245
H	-1.3033932827	-0.9213233612	-2.5021434334
H	-1.9893863998	-2.4402894852	-1.842179337
H	-2.630003839	-0.8343694949	-1.3667974168
C	-0.1955667175	3.4673057166	-0.3371540664
H	0.0929626551	3.5243158549	-1.3913246758
H	-1.27103389	3.6615648526	-0.2763617127
C	0.5966582281	4.4394895858	0.5094292979
H	1.6667683963	4.2179029762	0.4578276152
H	0.4395774003	5.4595061713	0.1471154905
H	0.2798518832	4.3965341213	1.5557463659
O	0.0764632425	2.1502515271	0.1521372172

TS1bMe-C3
E(RM062X) = -922.016247030
Sum of electronic and thermal Free Energies = -921.659305
Charge = 0 Multiplicity = 1

C	0.0566546219	-0.0666653773	-0.1278306505
C	0.0340967058	-0.0221521545	1.8261405772
C	1.1775364361	-0.0021371986	0.9145956607
H	-0.5181803687	0.9014345273	1.9650788024
C	-0.4632851272	-1.3565985711	-0.5498433039
O	-0.2096825895	-2.3910883615	0.0838071091
C	-0.4157707468	1.175078638	-0.7146445825
O	-1.2107965282	1.3126169136	-1.6334635406
H	1.6494681551	0.9778490433	0.8682435046
C	1.5585793114	-1.1006706437	5.7403284067
C	1.0526665283	-1.2609912771	4.2767505141
H	2.6432628857	-1.2334219301	5.795988731
H	1.0961133684	-1.8417736871	6.3985914554
H	1.8382072029	-1.6229567302	3.608717646
H	0.2060032189	-1.9517271279	4.2082724407
C	1.9730903814	1.2343806826	5.4534904148
H	1.6630571581	2.2289596304	5.7887037142
H	3.0417078592	1.1284243202	5.661494203
C	1.6804268477	1.0378173961	3.9372042206
H	1.3654840074	1.9681029701	3.4513583611
H	2.5539781991	0.6544335667	3.4010328958
C	-0.571508764	0.4854210007	4.5714785096
H	-0.84714826	1.4889886518	4.2301198045
H	-1.4026341874	-0.1912575154	4.3463244628
C	-0.1995426669	0.4740545936	6.0822547253
H	-0.4564608107	1.4263442299	6.5553202152
H	-0.7341235181	-0.3196486177	6.6127338503
N	1.2388838616	0.2399153348	6.2436879057
N	0.5974970724	0.0521922001	3.7857796173
H	-0.4719040038	-0.9701057823	1.9847211892
C	2.2224526655	-1.1067820036	0.935045783
H	2.9964373964	-0.8712897417	1.6735365949
H	1.7892224255	-2.0811277476	1.1574351582
H	2.7075163729	-1.1686274936	-0.0443953771
C	-1.3486196849	-1.4653585544	-1.7749171132
H	-2.3031314334	-0.9595482364	-1.6058192742
H	-0.8962007582	-0.9828552101	-2.6440946698
H	-1.5179029874	-2.5261987905	-1.9671303687
C	-0.3224954809	3.5430375901	-0.5960382627
H	-0.0484567562	3.6235576026	-1.652570626
H	-1.4132776293	3.6042502177	-0.5296659267
C	0.3500976223	4.6113861949	0.2380010957
H	0.0486136851	5.6021347216	-0.1138516112
H	0.0659942639	4.5197452986	1.2907688112
H	1.4386494817	4.535655756	0.1632634693
O	0.110754852	2.2749650934	-0.0938155812

TS1cPh-C2
E(RM062X) = -1228.20107409
Sum of electronic and thermal Free Energies = -1227.764871
Charge = 0 Multiplicity = 1

C	0.0221532924	-0.1035655082	-0.0037780023
C	0.0157075369	-0.063158223	2.0815448766
C	1.0857200582	-0.074629226	1.0700597766
H	-0.5515264998	0.8616852338	2.1418047064
C	-0.2704676675	-1.3996582042	-0.5743516689
O	0.3490116486	-2.4240577574	-0.2897317343
C	-0.5584567715	1.1342455395	-0.4691606834
O	-1.3749609166	1.316969671	-1.3563813448
H	1.6888577348	-0.9815181472	1.0541165341
H	1.7011307321	0.8236121312	1.0517949536
C	2.9338772607	0.205273648	5.3240099567
C	2.2651829507	-0.1954167747	3.976197639
H	3.9753516741	0.5006277455	5.1672267535
H	2.9237174479	-0.6299472824	6.0309056315
H	2.876193524	0.1043177773	3.1197168109
H	2.0873929911	-1.2735646778	3.913258457
C	2.0892085318	2.4031988338	4.9477223604
H	1.6696379967	3.2867248779	5.4376115789
H	3.0947880775	2.6582354559	4.5996003244
C	1.188428512	1.9474789105	3.7637401062
H	0.2064035669	2.4308750514	3.7936452569
H	1.6410403079	2.1671768893	2.7919687048
C	0.1658225607	0.2144633138	5.0713195855
H	-0.8419193163	0.6128503959	4.9159794243
H	0.0846762796	-0.8719769221	5.175348359
C	0.8644999203	0.868384592	6.2993786358
H	0.2987874171	1.7335948344	6.6587388801
H	0.9452812031	0.1543198402	7.1240094517
N	2.2093927023	1.3248311282	5.9341534573
N	0.9663486157	0.492293269	3.8640241342
C	-0.6860678391	-1.2738713945	2.5474443428
C	-0.0612495522	-2.5264804805	2.6053218507
C	-2.0037858956	-1.150096815	3.0121022241
C	-0.7379401801	-3.6242342595	3.1332190424
H	0.942997737	-2.6502176373	2.2164259238
C	-2.6809556183	-2.2470695314	3.5332441527
H	-2.4988297797	-0.1839461469	2.948881461
C	-2.0443109325	-3.4878726106	3.6005619918
H	-0.2458662167	-4.5912091247	3.1676395061
H	-3.7034373071	-2.1379801566	3.8813539696
H	-2.5694478186	-4.3474179037	4.0060081056
C	-0.548285333	3.4900350199	-0.1589202124
H	-0.2985981406	3.6580906461	-1.2114375489
H	-1.6394609282	3.5087166312	-0.0715162
C	0.1006194848	4.5235586071	0.7363329602
H	1.190618271	4.4818153269	0.6514955272
H	-0.2283877335	5.5259822084	0.4474109758
H	-0.1745368506	4.3623058947	1.7830917396
O	-0.0604605945	2.2085723807	0.2418702787
C	-1.5879637504	-2.6984900594	-2.0285340842
H	-0.6970332365	-3.0750152752	-2.5420202852
H	-1.8395433694	-3.4093481737	-1.2342442929
C	-2.7418417387	-2.4987369272	-2.9867729123
H	-2.4759668638	-1.7798636199	-3.766472895
H	-3.000073938	-3.4500917999	-3.4615376681
H	-3.6225036962	-2.122658246	-2.4589523788
O	-1.2968475933	-1.4248808347	-1.4474060348

TS1cPh-C3
E(RM062X) = -1228.19126737
Sum of electronic and thermal Free Energies = -1227.756482
Charge = 0 Multiplicity = 1

C	-0.0996504986	-0.1105517117	0.0636783269
C	-0.0381940387	-0.0630504546	2.0541039999
C	1.0513399875	-0.0692714501	1.0743491847
H	-0.5777256496	0.8715214807	2.1541106776
C	-0.6327410384	-1.4013086649	-0.3346460164
O	-0.5242954328	-2.4287160431	0.3305213261
C	-0.5935603424	1.1364598346	-0.4884357144
O	-1.4800817651	1.3059364644	-1.307581208
H	1.5265684937	0.9070753607	1.0029651711
C	-0.0481986325	1.1426562675	6.1243534071
C	-0.5330839057	0.7354606103	4.7041244403
H	-0.7061321388	0.7293843027	6.8941585974
H	-0.0401372157	2.2307979036	6.2382052096
H	-1.3279559864	-0.0167665871	4.7395883615
H	-0.9066726391	1.5991590693	4.1448400128
C	1.3332383497	-0.7960852093	6.1097282467
H	2.3173555882	-1.1853261709	6.3856274367
H	0.5919458619	-1.2602136669	6.7673241937
C	1.0250907568	-1.09668552	4.613651533
H	1.9105951344	-1.4595606643	4.0874231155
H	0.2273335362	-1.8378213739	4.4967812729
C	1.7231074502	1.1040106385	3.9450445904
H	2.507378996	0.6926520496	3.3019853959
H	1.3652126349	2.0369578309	3.4954207607
C	2.2200431784	1.3079587313	5.4062467438
H	3.2180817058	0.8804528416	5.5402762841
H	2.2740062156	2.3716126993	5.6557454218
N	1.3123561914	0.6498404132	6.3522831968
N	0.5963734755	0.1523917726	3.95258997
H	-0.5661917079	-0.9911853185	2.2531140511
C	2.1185757718	-1.1355450934	1.0273195125
C	2.0101651517	-2.3859978238	1.6420398805
C	3.2987709376	-0.8315185582	0.3385699265
C	3.0580892351	-3.3029816156	1.576096124
H	1.0920515495	-2.6546541283	2.1516961572
C	4.3458159567	-1.7479361799	0.2632282647
H	3.3928571264	0.1368318539	-0.1482731928
C	4.2291630957	-2.9889241166	0.8872644775
H	2.9528710094	-4.271542469	2.0561705072
H	5.2507690223	-1.4906701581	-0.2790884671
H	5.0420153611	-3.7069637479	0.8344118864
C	-0.4189498301	3.5017750445	-0.3642185226
H	-0.2614684095	3.5829361322	-1.4442681797
H	-1.49392049	3.5924275604	-0.1783133828
C	0.3695752869	4.5476215827	0.3938698884
H	0.0593214414	5.5482783151	0.0795073889
H	0.199678041	4.4586413957	1.4713826801
H	1.4411958206	4.4429951466	0.2010926805
O	0.0324643687	2.2207557544	0.0819574388
C	-1.8598398553	-2.6474591242	-1.9110697917
H	-2.5667794876	-2.9668173306	-1.1381036588
H	-1.0806739307	-3.4128272065	-1.9868610094
C	-2.5491120792	-2.4154784723	-3.2377563172
H	-3.0240924362	-3.3410513322	-3.5765000392
H	-3.316071445	-1.642066428	-3.1440616384
H	-1.8295283076	-2.0964169449	-3.9966352323
O	-1.2659936385	-1.4050425379	-1.5217498262

TS1cMe-C2
E(RM062X) = -1036.52040151
Sum of electronic and thermal Free Energies = -1036.133629
Charge = 0 Multiplicity = 1

C	0.0460798309	-0.1116599983	0.0042318842
C	0.0306710043	-0.0641010455	2.0589666177
C	1.1163776017	-0.0638969609	1.0741335668
H	-0.5684898645	0.8394688468	2.10982377
C	-0.2569018524	-1.4121957515	-0.5450249494
O	0.2524893438	-2.457187805	-0.1380581734
C	-0.497013783	1.1281059795	-0.5082004355
O	-1.2824005772	1.303139411	-1.4234832731
H	1.7183723881	-0.9725323973	1.0836239804
H	1.7209354975	0.8407978989	1.0329026655
C	2.9305091278	0.4709821345	5.3249547213
C	2.2850193417	-0.014622571	3.9946564942
H	3.9594695564	0.8029796392	5.1590282952
H	2.952828235	-0.3316183782	6.0686502524
H	2.8800355566	0.2858031236	3.1272894889
H	2.1717992413	-1.103455643	3.9686724406
C	1.9868716044	2.6085641875	4.8461470596
H	1.5337611807	3.4971159117	5.2956677312
H	2.9797781721	2.88741405	4.4801242519
C	1.1007941201	2.0567069274	3.6939754364
H	0.0957734808	2.4916281368	3.7138285379
H	1.5299566713	2.2570555257	2.7073100568
C	0.1713641568	0.3374904578	5.0806205978
H	-0.8585062415	0.6699286815	4.9110656034
H	0.1582124589	-0.7438448862	5.2463701477
C	0.8327834395	1.0886032345	6.2732646861
H	0.2269315005	1.9456079983	6.5837679851
H	0.9427658167	0.4254151689	7.1362606781
N	2.1564372286	1.585123617	5.8838303868
N	0.9515489203	0.5978966323	3.8569155812
C	-0.5444471595	-1.3419007566	2.5970761168
H	0.2422035914	-1.9472265025	3.0593768937
H	-1.32449605	-1.1363918844	3.3324399715
H	-0.9726230796	-1.9511206663	1.798963834
C	-0.4779017458	3.4865264267	-0.2319012167
H	-0.1904695556	3.6410953176	-1.2767896096
H	-1.5712725433	3.5097409598	-0.1831500027
C	0.1428020463	4.5279265607	0.6739626063
H	1.2350452657	4.4806945357	0.6305677101
H	-0.1701699364	5.5278163709	0.3595347699
H	-0.172989596	4.3823048909	1.7114018693
O	-0.0101825028	2.2067700927	0.1997778507
C	-1.466852041	-2.7057187414	-2.0966439824
H	-0.5453855522	-3.1535652416	-2.4829953286
H	-1.8528092291	-3.365374161	-1.3122579455
C	-2.485281907	-2.4875125901	-3.1941300984
H	-2.086065485	-1.8225789839	-3.9646705646
H	-2.7430885787	-3.4444485426	-3.6576470011
H	-3.3972071485	-2.0379383653	-2.7919374453
O	-1.1705831071	-1.4236675515	-1.5356613493

TS1cMe-C3
E(RM062X) = -1036.51875585
Sum of electronic and thermal Free Energies = -1036.131040
Charge = 0 Multiplicity = 1

C	0.0075966041	-0.1324214973	0.0071382581
C	0.0084474602	-0.0895044102	1.9780930501
C	1.1338328504	-0.0864467995	1.0394789695
H	-0.5380230252	0.8390748468	2.1072331348
C	-0.5007090222	-1.4302602701	-0.3992618182
O	-0.3128127996	-2.4739825551	0.2255571469
C	-0.4806092569	1.1107306217	-0.5632988016
O	-1.313773621	1.274306091	-1.4373621038
H	1.6212311093	0.8852713992	0.9852192803
C	1.5792936334	-1.153010559	5.8534930404
C	1.0464087191	-1.3212909749	4.4007562794
H	2.6643904466	-1.2888459893	5.8900578158
H	1.1266724347	-1.888711168	6.5244762619
H	1.8158312356	-1.6973602129	3.7220738549
H	0.1909939591	-2.002936224	4.3537395209
C	2.0000181379	1.1782348042	5.5471129796
H	1.6962344058	2.1761013223	5.8780785624
H	3.0708415647	1.071594921	5.7428004017
C	1.6890685007	0.9708874418	4.0362029519
H	1.3760861726	1.8989998861	3.5452292952
H	2.5531160115	0.5750043099	3.4937745362
C	-0.5590705265	0.4410680948	4.70616043
H	-0.8287068532	1.4459300777	4.3642321232
H	-1.3997049892	-0.2280340511	4.4942188193
C	-0.166531049	0.4313500103	6.2114440015
H	-0.4136612023	1.3856916906	6.6854555189
H	-0.6970660451	-0.359068054	6.7508119284
N	1.2729895452	0.1917098501	6.353746506
N	0.5955616802	-0.0072555606	3.9062134866
H	-0.5103135321	-1.0297129128	2.1418758007
C	2.1630662441	-1.2058879429	1.0501621876
H	2.9418026955	-0.9904652423	1.7896263386
H	1.716271182	-2.1759451649	1.2667590463
H	2.6451110773	-1.2663435916	0.0690310796
C	-0.3513224712	3.4766136164	-0.412796589
H	-0.1264469954	3.5724670459	-1.4797596494
H	-1.437531899	3.5464509228	-0.2957987189
C	0.3670782437	4.5288642164	0.4040062616
H	0.0577802009	5.5269370507	0.0804890608
H	0.1311460527	4.4243636982	1.4675423346
H	1.450547547	4.4464664815	0.2785052255
O	0.093851954	2.1998395125	0.0500397932
C	-1.7688309068	-2.6883007102	-1.9335850076
H	-2.4176609063	-3.0650491537	-1.1358219854
H	-0.9585297846	-3.4115236236	-2.0718490894
C	-2.5369070902	-2.4563191205	-3.2163640749
H	-2.9814532043	-3.3956917086	-3.558473701
H	-3.3362149174	-1.7266652336	-3.0617542085
H	-1.8746929501	-2.0790394422	-4.0003321237
O	-1.2177904415	-1.4285282019	-1.5390681325

DABCO(DMSO)
E(RM062X) = -345.204637295
Sum of electronic and thermal Free Energies = -345.050239
Charge = 0 Multiplicity = 1

C	-0.0037295404	0.0018997408	0.0002626308
C	0.0029495282	-0.0026784276	1.5554703082
H	1.0152356484	0.0090588374	-0.4004263879
H	-0.5275016626	0.880752459	-0.390542981
H	1.0174659643	-0.1341239427	1.9464469715
H	-0.3952496623	0.9353048383	1.956143484
C	-0.0566686704	-2.3876574403	0.0755047228
H	-0.51492693	-3.2768705817	-0.3696206031
H	1.0010229649	-2.3737254954	-0.208079775
C	-0.223869246	-2.382377718	1.6217449974
H	-0.8812051553	-3.1937860676	1.9516425038
H	0.741240197	-2.5023928751	2.1248646188
C	-2.1694175264	-0.9992250195	1.479790769
H	-2.8037195542	-1.7728304011	1.9248229533
H	-2.5765889726	-0.0229498753	1.7634675968
C	-2.0979747966	-1.1504155319	-0.0664996439
H	-2.5819579242	-2.0757056349	-0.3965708328
H	-2.5911682149	-0.3122984061	-0.5698023846
N	-0.8219413285	-1.111618495	2.0609187339
N	-0.6939805968	-1.1951748264	-0.5055423188

1d
E(RM062X) = -883.751890232
Sum of electronic and thermal Free Energies = -883.501777
Charge = 0 Multiplicity = 1

C	0.1896724522	-0.1627618122	-0.0364127278
C	0.2627488026	-0.2047633999	1.4959485455
C	1.5037465295	-0.3644176615	0.6844619238
H	0.1256201632	0.7492940301	1.9942887903
H	2.2060552402	0.4630480786	0.6677936807
H	1.931577802	-1.3577068061	0.5877011019
C	0.0524778475	1.1067936744	-0.819580519
O	-0.1015200362	1.0390020018	-2.0288496595
C	-0.4125346739	-1.3672465569	-0.7278453637
O	0.2597605329	-2.3447671593	-1.0009920633
C	-0.3425620192	-1.3607239103	2.1995663643
H	-0.1340030672	-2.3517945035	1.7956838216
C	-1.1062719612	-1.2319685245	3.2861390288
H	-1.52924143	-2.0999728117	3.782642868
H	-1.3241021781	-0.2540877804	3.7107748573
C	-1.8774423552	-1.3297653322	-1.0234168285
C	-2.4025557835	-2.2400700064	-1.947997543
C	-2.7290135176	-0.4283649687	-0.3743308243
C	-3.765149495	-2.2413536717	-2.2292367966
H	-1.7331531597	-2.9348958328	-2.4459304791
C	-4.0954132736	-0.4400005888	-0.6479285498
H	-2.3351692305	0.2713106004	0.3584139572
C	-4.6128297096	-1.3421258583	-1.5776424988
H	-4.1681688415	-2.9410801933	-2.9549082496
H	-4.7544028793	0.2541639392	-0.13587313
H	-5.6768033317	-1.3456623074	-1.7951108017
C	0.1992213734	2.4456152178	-0.1606492514
C	1.1572272244	3.3288431078	-0.6708737017
C	-0.6409667526	2.8533919771	0.8809847956
C	1.2913488735	4.6033065946	-0.1252595936
H	1.7981475236	3.0096962692	-1.4880063663
C	-0.5203995484	4.1390519325	1.4067600863
H	-1.4040308841	2.1824148495	1.2651905334
C	0.4506267629	5.00996907	0.9121390605
H	2.0461387209	5.2804137933	-0.5132180296
H	-1.1846163726	4.4586338761	2.2037846744
H	0.5495323048	6.0064497274	1.3321645121

TS1d-C2
E(RM062X) = -1228.93209264
Sum of electronic and thermal Free Energies = -1228.505787
Charge = 0 Multiplicity = 1

C	0.0825783174	-0.1284748383	-0.033711717
C	0.0375106098	-0.0950846941	1.9642494545
C	1.161843773	-0.2042556156	1.033551288
H	-0.4337555325	0.8730953979	2.1020355628
C	-0.7033428833	-1.2811763882	2.4108199377
H	-0.198599503	-2.239944729	2.309875005
C	-1.9307343442	-1.2061440679	2.9360283633
H	-2.4509615035	-2.0941332612	3.283086575
H	-2.4480901852	-0.252942016	3.0273478969
C	-0.4210384994	-1.3966413288	-0.5492218377
O	0.2161096724	-2.4491620708	-0.4268248933
C	-0.3046352454	1.1486375048	-0.5907833635
O	-0.9979433754	1.2937499549	-1.6054470102
H	1.6568588605	-1.1744193204	1.0422491104
H	1.8693234451	0.6185885975	1.0308605082
C	2.5727500258	-0.2058750608	5.6926220666
C	1.7541958669	-0.7336010888	4.4774997213
H	3.640722188	-0.1661908392	5.4566161381
H	2.4384848404	-0.8572284299	6.5613016068
H	2.4047441574	-1.1547549871	3.7038439299
H	1.0336177856	-1.4996791929	4.7812706835
C	2.4722077178	2.0621233961	4.9414910227
H	2.0335545644	3.0403127593	5.1625724033
H	3.5590677409	2.1775239426	4.8935783272
C	1.9232484579	1.4937873777	3.6022856892
H	1.3825922309	2.2544880308	3.0243276801
H	2.7352284871	1.1003741757	2.9840920027
C	-0.0140257852	0.8433924931	4.8548024163
H	-0.490781676	1.7357313947	4.4333450351
H	-0.7722197228	0.061605028	4.9607064959
C	0.6760918686	1.1490347531	6.2145761985
H	0.3601599404	2.1232717539	6.5992991782
H	0.4232459635	0.3882312493	6.9595393472
N	2.136091493	1.1535941596	6.0472298065
N	0.9996612692	0.3847106604	3.8941836516
C	-1.7990638516	-1.4807218531	-1.14522154
C	-2.0353946442	-2.3822658136	-2.1862888442
C	-2.8687061974	-0.7632038709	-0.5989718369
C	-3.3218192879	-2.5441147151	-2.6995875789
H	-1.2038305542	-2.9497226325	-2.5943166318
C	-4.158515584	-0.9441185387	-1.0927017596
H	-2.6890896594	-0.0668303471	0.2162322217
C	-4.3861934173	-1.82848443	-2.1497999617
H	-3.4948629601	-3.2330770407	-3.5211219088
H	-4.9867432138	-0.3936738787	-0.6560891053
H	-5.3909215258	-1.9611821811	-2.5404894621
C	0.1857624258	2.4107924321	0.0927744859
C	1.4949590893	2.8686406875	-0.0814715962
C	-0.7103093999	3.1640697846	0.8580126971
C	1.9052510553	4.0640616379	0.5097657258
H	2.1944458754	2.2895617171	-0.6800926144
C	-0.2969921128	4.3538049839	1.4577869565
H	-1.7317442598	2.8128466386	0.9892806001
C	1.0122872155	4.8054044016	1.2849176533
H	2.9232404692	4.4144999087	0.3665353645
H	-0.9975257262	4.9279683327	2.0571880815
H	1.3345976905	5.7321447261	1.7503037677

TS1d-C3
E(RM062X) = -1228.92603013
Sum of electronic and thermal Free Energies = -1228.500722
Charge = 0 Multiplicity = 1

C	-0.1942139577	0.1093929254	0.2428651455
C	-0.112459483	-0.0093811454	2.1329616023
C	1.0093212867	0.0358154704	1.1972548802
H	-0.5670016849	0.9276001444	2.4363349861
C	-0.7699729117	-1.1247352804	-0.2676065609
O	-0.6460546635	-2.2140009692	0.3084951033
C	-0.7261264146	1.4078397843	-0.170395584
O	-1.9046449256	1.5701281802	-0.4948323728
H	1.5413773206	0.9855039413	1.2324353577
C	1.6964570462	-1.5327540965	5.8572924358
C	1.0603970461	-1.5325198221	4.4370536974
H	2.7750376134	-1.7094633889	5.8007606393
H	1.2542403528	-2.3148073473	6.4815486759
H	1.773759606	-1.8635246708	3.6769993887
H	0.1769660838	-2.1773911705	4.3886536544
C	2.1960888438	0.8033915256	5.7464633826
H	1.955781146	1.7744913407	6.1900665164
H	3.2723411053	0.6370488395	5.8504713026
C	1.7730894354	0.7546025301	4.2502977288
H	1.4689409229	1.7402427873	3.880960142
H	2.5825647493	0.3813301783	3.614601381
C	-0.4434090019	0.2445519195	5.0237012652
H	-0.69077307	1.2879653382	4.8025318727
H	-1.3239010443	-0.3716983026	4.8134788851
C	0.0451911674	0.0728324151	6.4897155524
H	-0.1284116603	0.9863416138	7.0661277407
H	-0.4815005891	-0.7490769058	6.9842076331
N	1.4829977278	-0.2323103026	6.5090915476
N	0.6331822208	-0.1635530121	4.1074090959
C	1.9634479029	-1.1051307191	1.0653851707
H	1.5498794538	-2.1093830576	1.1000799928
C	3.2739251256	-0.9129259611	0.9026784901
H	3.7006335828	0.0883147959	0.8669782223
H	3.9594632202	-1.7498668934	0.8030083036
H	-0.6540593976	-0.9376325581	2.2783555642
C	-1.479883285	-1.1203134239	-1.5973853041
C	-2.5875424301	-1.9550403036	-1.7696012927
C	-1.0011461289	-0.3808046572	-2.6835602429
C	-3.2313691581	-2.0259413789	-3.004035718
H	-2.944626128	-2.5411638776	-0.9276920763
C	-1.6297907467	-0.4684808982	-3.9239809064
H	-0.1293148561	0.2579317272	-2.5627436722
C	-2.7518427088	-1.2837644871	-4.0844289324
H	-4.1024494823	-2.6632651409	-3.1247983653
H	-1.2451175733	0.1001158894	-4.7655384799
H	-3.2469834015	-1.3441111838	-5.0491810136
C	0.1822765119	2.6102419726	-0.182584054
C	-0.3073353594	3.8380848973	0.2729525865
C	1.4734875865	2.53762673	-0.7183032924
C	0.4922485301	4.9783655307	0.2145307382
H	-1.3152182747	3.8902191393	0.6750777242
C	2.2635970433	3.6827172156	-0.8008688251
H	1.8543003869	1.5845509366	-1.0798082498
C	1.7769178723	4.9022667259	-0.3261344904
H	0.1126797093	5.9261628027	0.5844887518
H	3.2588982313	3.6233932484	-1.2309551305
H	2.3974424865	5.7919724169	-0.3792052241

TS1d-Cbetaanti
E(RM062X) = -1228.92676076
Sum of electronic and thermal Free Energies = -1228.501484
Charge = 0 Multiplicity = 1

C	-0.2804725638	-0.0582220736	0.2038526885
C	-0.2726127658	-0.1152259575	2.0537368541
C	0.8895583173	-0.1583291096	1.1661680418
H	-0.5991453717	0.8822427899	2.3493090928
C	-0.8774318364	-1.2050516056	2.7452761487
H	-0.4889641543	-2.2056428085	2.5895497146
C	-1.8921385527	-0.9782416931	3.6584781811
H	-2.474989952	-1.8148195115	4.0384650138
H	-2.4104317085	-0.0194778453	3.6488706677
C	-0.7477729357	-1.3080827449	-0.3825434021
O	-0.4318172518	-2.4037979334	0.0912506082
C	-0.8816743821	1.2303795869	-0.1551361289
O	-2.0602339824	1.3406649255	-0.4965847759
H	1.4035760703	-1.1152122719	1.1050320234
H	1.5555993641	0.6994025479	1.1915709403
C	0.0008114748	-1.5519275907	7.4630565071
C	-0.4556824797	-1.7914874527	5.9952956318
H	1.0747661645	-1.7298647267	7.5697311127
H	-0.527742406	-2.222322854	8.1473289674
H	0.3939551263	-1.9306435948	5.3188762176
H	-1.1133909994	-2.6626568769	5.9123129866
C	0.337452902	0.7401564207	6.8940753313
H	0.2072296673	1.7694439293	7.2403918177
H	1.4088489782	0.5200240004	6.8670053086
C	-0.3050175795	0.5503631104	5.4900757341
H	-0.9032533373	1.4195074266	5.1972778367
H	0.4486519119	0.3675749897	4.7160494536
C	-2.3204218023	-0.3436672663	6.4440227297
H	-2.9217985884	0.4656529122	6.0177196941
H	-2.934888066	-1.2481940968	6.4920075645
C	-1.7441427504	0.0385484592	7.8397196613
H	-1.9406104383	1.0908885792	8.0655299859
H	-2.1984092543	-0.5717790061	8.6254319403
N	-1.1994396219	-0.6118561396	5.5336196178
N	-0.2889311032	-0.1682908957	7.8648614465
C	-1.5745559404	-1.3028484989	-1.6406218705
C	-1.2377872378	-0.5022913345	-2.7366831034
C	-2.639778792	-2.2010434422	-1.7459595803
C	-1.9642506134	-0.5951488382	-3.9218756708
H	-0.4008814633	0.1886501378	-2.6662511343
C	-3.3836207083	-2.2760047348	-2.9230561043
H	-2.8858373249	-2.8342447607	-0.8982349649
C	-3.0448029887	-1.4750983163	-4.0143007237
H	-1.6893820715	0.0202566023	-4.7734857356
H	-4.2216398218	-2.963561333	-2.9910028759
H	-3.6166971879	-1.5391214048	-4.9353760496
C	-0.0484756188	2.4810841753	-0.0563947348
C	1.2529662195	2.5282763173	-0.5678679131
C	-0.6221320756	3.6375083118	0.4814954977
C	1.9705916071	3.7231480852	-0.5461154686
H	1.6973650839	1.632631599	-0.9957619469
C	0.1044447673	4.8257078644	0.5264014373
H	-1.6378634626	3.5959975221	0.8649283795
C	1.4004653015	4.8700952877	0.0090037242
H	2.9744113394	3.7586411158	-0.9585991558
H	-0.340094231	5.717072967	0.9589678169
H	1.9647257126	5.7976094343	0.0361258865

TS1d-Cbetasyn
E(RM062X) = -1228.93434867
Sum of electronic and thermal Free Energies = -1228.506690
Charge = 0 Multiplicity = 1

C	0.0383991958	0.0016830405	0.1360969833
C	-0.0789416461	0.0266522805	1.9099168811
C	1.1631603501	-0.1201864722	1.1457959936
H	-0.5862528019	-0.9051390926	2.1546294103
C	-0.5301255417	1.1927773138	2.6085368425
H	-0.0227613806	2.1421052063	2.4469320664
C	-1.6675005051	1.1319409455	3.4043134077
H	-1.8676033103	1.9519682941	4.0923470444
H	-2.0014345435	0.154273801	3.753793707
C	-0.5783598567	-1.2357376624	-0.3287788206
O	-0.3698577812	-2.3123162129	0.2415982577
C	-0.311736882	1.3125510295	-0.4443214621
O	-1.4634727994	1.5979604121	-0.7630391162
H	1.64236852	-1.0960516741	1.1557348431
H	1.8489992659	0.721912509	1.1853896045
C	-4.6459380486	2.7991515751	0.8963671347
C	-3.368860438	2.6892589001	1.775085101
H	-4.3887928849	2.7845520499	-0.1671839859
H	-5.1802496019	3.7301029421	1.1072633493
H	-2.4552463424	2.7454676544	1.1748239848
H	-3.3387223055	3.4645097416	2.547868606
C	-4.8733703084	0.4273278124	0.751792445
H	-5.5240838403	-0.4148715121	1.0068108026
H	-4.7438470525	0.4426229415	-0.3353279714
C	-3.4963075517	0.3040103238	1.4621901769
H	-3.3926913228	-0.6501579305	1.9905900132
H	-2.6695266504	0.4181775472	0.7569078165
C	-4.5071692108	1.3251281825	3.3932701503
H	-4.5088330165	0.3285363066	3.8463756499
H	-4.3403528852	2.0678721002	4.1798688489
C	-5.8203613174	1.6045809592	2.6038840719
H	-6.5555229999	0.8155950737	2.7863995026
H	-6.2589031695	2.5595325754	2.9084029245
N	-3.3766514263	1.3872241637	2.455370582
N	-5.5477404365	1.6680241604	1.1609819906
C	-1.4833145106	-1.233467701	-1.5299218877
C	-1.0876711401	-0.6672431008	-2.7449796798
C	-2.7175913949	-1.8827258882	-1.4381598539
C	-1.9221294157	-0.7474195698	-3.8580174224
H	-0.12422885	-0.1682415867	-2.8225581362
C	-3.5644859957	-1.9388992832	-2.5446648917
H	-3.015159334	-2.3321100767	-0.4941676445
C	-3.1658204559	-1.3745515217	-3.7571135399
H	-1.6044534297	-0.3172984934	-4.8032245111
H	-4.5311032677	-2.4272572298	-2.4619547903
H	-3.8204187702	-1.425277879	-4.6222217035
C	0.7552208963	2.3559530619	-0.6124358584
C	0.4235639244	3.6974020451	-0.3957488306
C	2.0418772133	2.0215854135	-1.0491054083
C	1.3751651382	4.6953226445	-0.5945385113
H	-0.5812522279	3.9481498957	-0.0665231438
C	2.9853293776	3.0223906942	-1.2731640086
H	2.2991047982	0.9799165704	-1.2251544708
C	2.6555550237	4.3583887354	-1.0375621827
H	1.1186796709	5.7341971041	-0.4101122405
H	3.9779824551	2.7603747773	-1.6265823395
H	3.395662561	5.136376163	-1.2000475006

int1d-C2
E(RM062X) = -1228.97568953
Sum of electronic and thermal Free Energies = -1228.542865
Charge = 0 Multiplicity = 1

C	-0.0171827938	-0.0145447864	0.0117455443
C	-0.0105811682	0.0058537393	2.529777121
C	0.8533198862	-0.0090414393	1.252503265
H	-0.743709519	0.8154524508	2.4601909321
C	-0.7320641146	-1.3031726187	2.694179965
H	-0.1199254749	-2.202078959	2.7459487429
C	-2.060670268	-1.3949142053	2.6621814285
H	-2.5622348326	-2.3578989469	2.7027818766
H	-2.685957736	-0.5068470337	2.5860888215
C	-0.2827650929	-1.2963974323	-0.5681565614
O	0.3328097283	-2.3350217964	-0.2424905955
C	-0.4915106691	1.2143864809	-0.5323597766
O	-1.0916077472	1.3860015159	-1.6156503814
H	1.4713603862	-0.9115507186	1.2478222659
H	1.5222817865	0.8505430559	1.2422599594
C	2.6749973249	-0.0917856453	5.3145088764
C	1.7821291153	-0.6817944885	4.1966734996
H	3.6405932163	0.2232858942	4.9105148969
H	2.8516625344	-0.8569524754	6.0739382999
H	2.3567319552	-0.9600196031	3.3122679855
H	1.2093573925	-1.5422491288	4.5466688343
C	2.0227929305	2.1742990906	4.9654728614
H	1.3702023599	2.9635681227	5.3484247384
H	3.0313628098	2.5808393204	4.8595044197
C	1.5343260513	1.6714441523	3.5903186517
H	0.8525484442	2.3697551666	3.0965561358
H	2.3780815148	1.4560396189	2.9359754884
C	-0.1738042393	0.5806175441	4.9420397084
H	-0.7475685709	1.478745065	4.6982711301
H	-0.8441992107	-0.2773517671	4.9757883567
C	0.6423118576	0.7297118578	6.2428032445
H	0.1994172053	1.5143305135	6.8609457207
H	0.6355010334	-0.2037244607	6.8119586414
N	2.0335608143	1.073577745	5.9348164527
N	0.7855555776	0.372699803	3.7928497285
C	-1.4010687528	-1.4853645711	-1.5662700259
C	-1.1650952439	-2.2148487321	-2.7338060061
C	-2.7010545964	-1.0555185251	-1.2816524098
C	-2.2054345989	-2.4819591567	-3.6254961196
H	-0.1593100232	-2.5685707763	-2.9444686759
C	-3.7471253976	-1.3456251931	-2.1546067086
H	-2.8884140558	-0.4875506842	-0.3732724373
C	-3.50030554	-2.0516560127	-3.3352246326
H	-2.0070034633	-3.0339932293	-4.539946947
H	-4.7557126065	-1.0175845637	-1.9188475035
H	-4.314152664	-2.2685072192	-4.0211851871
C	-0.2316746902	2.4764794176	0.2768152246
C	0.9758561707	3.1733580608	0.1669780889
C	-1.2384054589	2.9873362746	1.1025739832
C	1.1780144804	4.3562164459	0.879713255
H	1.7641071962	2.7838843591	-0.473897607
C	-1.0383417963	4.1692210937	1.8180265849
H	-2.1822524391	2.4520191463	1.1893670965
C	0.1724251034	4.8554717797	1.7098030886
H	2.1212699586	4.887376556	0.7880051287
H	-1.8268448669	4.5530198815	2.4593949623
H	0.3304945484	5.7741432863	2.2671286963

int1d-Cbetaanti
E(RM062X) = -1228.97910707
Sum of electronic and thermal Free Energies = -1228.548741
Charge = 0 Multiplicity = 1

C	-0.0324029534	-0.0355527304	-0.0050407288
C	-0.0494572773	-0.076088209	2.4985273705
C	0.8074030593	-0.0669677028	1.2623189586
H	-0.7067660328	0.7883947169	2.6316366907
C	-0.0878444467	-1.0558677403	3.4066722633
H	0.5422343012	-1.9352188952	3.281338547
C	-1.0255428704	-1.0358976582	4.5729778632
H	-1.5503394029	-1.989590892	4.6804671071
H	-1.7579945232	-0.2301311822	4.4783809834
C	-0.3365414247	-1.3086910672	-0.5766818911
O	-0.0514021303	-2.3949889111	-0.0222616777
C	-0.650762151	1.176482566	-0.4405521451
O	-1.6234563696	1.2808699873	-1.2190545186
H	1.4336044669	-0.9642074984	1.2564519682
H	1.4705519046	0.8034980968	1.3141007808
C	1.246994467	-1.6773412154	7.5900021623
C	0.7859883833	-1.7871374335	6.1181484131
H	2.3385979655	-1.6794924954	7.6298356541
H	0.8769673014	-2.5243211714	8.1735306659
H	1.5763428413	-1.5098277778	5.4187867104
H	0.4089188565	-2.7808794565	5.8675714227
C	1.0393156633	0.6812215734	7.3071239197
H	0.8129815408	1.6196682388	7.8184606561
H	2.107888313	0.6608910944	7.078280253
C	0.204307186	0.5866400924	6.0095217622
H	-0.6607082691	1.253872405	6.0157286213
H	0.80086174	0.7729720694	5.1157413025
C	-1.3532967057	-1.0081437728	7.0072479294
H	-2.23539926	-0.4216104029	6.7443435351
H	-1.6084315967	-2.0701728226	7.0026346386
C	-0.7160133179	-0.5561377117	8.3399119773
H	-1.1078566084	0.417984335	8.6433586875
H	-0.9496712776	-1.2826203246	9.1215734071
N	-0.3425887024	-0.811931555	5.9068891099
N	0.739211692	-0.44367532	8.1991479308
C	-0.9413563103	-1.4171505463	-1.9584841962
C	-0.4092114542	-0.7200014405	-3.0481555198
C	-1.9815920967	-2.3240729153	-2.1763447479
C	-0.9142396067	-0.9203857029	-4.3309468412
H	0.4025439386	-0.0152857804	-2.8855753055
C	-2.5081528229	-2.5085056575	-3.4559050054
H	-2.3807330561	-2.8831783445	-1.3343535946
C	-1.9729671463	-1.808825593	-4.5377554499
H	-0.4856341012	-0.3816022538	-5.1714997713
H	-3.3288343937	-3.2038041381	-3.6092026605
H	-2.3726293044	-1.958147745	-5.5367153602
C	-0.1131176417	2.487768651	0.1053137199
C	1.2066003487	2.8892571872	-0.1279542796
C	-0.9769483665	3.3544569844	0.7822411047
C	1.654848612	4.1368627245	0.3065979027
H	1.8832559667	2.2224764814	-0.6584921871
C	-0.5255172648	4.5929908667	1.2385550225
H	-2.0070632142	3.0513771106	0.9522281937
C	0.7916210605	4.9884344912	0.9983760507
H	2.6779119826	4.4432864874	0.1077325841
H	-1.2019993515	5.2516982455	1.7758865115
H	1.1424995637	5.9561407831	1.3448735735

int1d-Cbetasyn
E(RM062X) = -1228.98309588
Sum of electronic and thermal Free Energies = -1228.550437
Charge = 0 Multiplicity = 1

C	0.2070684267	0.047415164	0.1821710213
C	-0.1012435445	0.0131734623	2.62758864
C	0.9211215402	0.0913044859	1.5275008698
H	-0.7350028242	0.8905811145	2.7757616085
C	-0.3717280862	-1.1038380372	3.3085294578
H	0.2450178699	-1.984768058	3.1466231715
C	-1.5635081974	-1.2503902903	4.1993080049
H	-1.9381611817	-0.2828637786	4.5439253287
H	-1.362777133	-1.8916304379	5.0613175287
C	-0.4403296181	-1.190161252	-0.1225586007
O	-0.2264109335	-2.2445195161	0.5212800347
C	0.2883155289	1.111789113	-0.7684285565
O	0.0482966477	1.010766214	-1.9914963227
H	1.6007691781	-0.7656561034	1.6160907945
H	1.5205906126	1.0012080175	1.6163664592
C	-4.5226314776	-1.6526449587	1.7839196566
C	-3.1948750937	-1.0640876266	2.3149875902
H	-4.4815736305	-1.7113028098	0.6924112391
H	-5.3658560173	-1.0165540286	2.0652120039
H	-2.3976565966	-1.1080394126	1.5718355951
H	-3.302418015	-0.0383035195	2.6746261777
C	-3.5504488373	-3.8012561767	2.1174362693
H	-3.7588187924	-4.8383641959	2.3906160822
H	-3.3018212226	-3.7669509436	1.0524962618
C	-2.3668243913	-3.2681437348	2.9595922412
H	-2.158321958	-3.8851156997	3.8368853636
H	-1.4676694924	-3.1526161545	2.3516530012
C	-3.8811099145	-2.0463915921	4.4466422485
H	-3.4878851482	-2.5296630392	5.3426273536
H	-4.2043993389	-1.0327584857	4.6934865565
C	-4.9957354257	-2.8780070184	3.7714668161
H	-5.03080676	-3.8851931763	4.1942297646
H	-5.9630776763	-2.3984919815	3.9383411464
N	-2.736274009	-1.9027511904	3.480141921
N	-4.7542398894	-2.9927634422	2.3307214793
C	-1.5439290916	-1.2690820258	-1.1549042012
C	-1.6549300906	-2.4154938801	-1.9466711908
C	-2.5405297876	-0.2897329517	-1.2378513247
C	-2.7226926701	-2.5686251128	-2.8323500671
H	-0.8957848699	-3.1883969072	-1.8635324019
C	-3.6198718674	-0.4487039617	-2.1045189313
H	-2.4684421847	0.5988410079	-0.6147148116
C	-3.7106977781	-1.5865026638	-2.910678949
H	-2.7883893264	-3.4575588168	-3.4534656944
H	-4.3927700931	0.3135232669	-2.1508149881
H	-4.5491926661	-1.7080734137	-3.5904087885
C	0.7321332643	2.4832750245	-0.298466111
C	1.7217448532	3.1607426637	-1.0187422787
C	0.1231240924	3.1251169699	0.7847672127
C	2.1168224527	4.4450432834	-0.6466157044
H	2.1820911002	2.6701051916	-1.8720193977
C	0.4991856282	4.4195118104	1.1456573448
H	-0.6612104713	2.6148269216	1.338332832
C	1.503619966	5.0789367527	0.4359702846
H	2.8980001566	4.9544942669	-1.2036957017
H	0.0086616106	4.912507363	1.9801305906
H	1.8040343123	6.0828290688	0.7216960562

TS2d-C2
E(RM062X) = -1228.92668779
Sum of electronic and thermal Free Energies = -1228.498558
Charge = 0 Multiplicity = 1

C	-0.009951874	-0.0601431872	-0.0296475575
C	-0.0332634862	-0.0941072751	2.4418154717
C	0.8540867179	-0.0295853812	1.2214978323
H	-0.1022292314	0.7300668396	3.1393055291
C	-0.8387815318	-1.3043753897	2.676968976
H	-0.5859542897	-2.1735770039	2.0708363035
C	-1.8274017722	-1.3631999564	3.5715820603
H	-2.4004604643	-2.274074265	3.7193815369
H	-2.1024706598	-0.4947194231	4.1662456885
C	-1.2359391939	0.5603366655	0.2198199895
O	-1.4845849565	0.9099447915	1.4242114914
C	0.3490537147	-0.7584684261	-1.2365279549
O	-0.4447869973	-1.0718149316	-2.1380017305
H	1.5283175728	-0.8901927159	1.233170294
H	1.4656692058	0.8784965429	1.279036741
C	3.7544559358	-0.0791381348	4.7323591429
C	2.7443824972	0.1251404518	3.5680215939
H	3.7783743118	0.7979148093	5.3864042232
H	4.7621657066	-0.2409363498	4.3388983402
H	2.4252980811	1.1695931965	3.4906182163
H	3.1804819172	-0.1794682877	2.6123581877
C	2.091738517	-0.9342328954	6.2082555242
H	1.7397628584	-1.8453731259	6.7017034846
H	2.2650374487	-0.1743039815	6.9757192494
C	1.0517350317	-0.4310915766	5.1690058056
H	0.0884208895	-0.9338864788	5.2957072732
H	0.8961299196	0.6497192824	5.2486540022
C	1.9308159143	-2.1319933135	3.7240238094
H	1.0677375848	-2.724589785	4.040458382
H	2.1569308573	-2.3751443857	2.6805444441
C	3.1581163397	-2.3854419437	4.644522043
H	3.0039249186	-3.286194072	5.2457932228
H	4.0689194345	-2.5200787766	4.0530749552
N	1.5504809842	-0.7097851922	3.8103928145
N	3.3649067453	-1.2405322213	5.5423198587
C	1.7925186769	-1.1746401898	-1.4147844969
C	2.0655351861	-2.4287094777	-1.97159496
C	2.8557822374	-0.321538229	-1.0992783819
C	3.380940954	-2.8357417492	-2.1867522186
H	1.2366137107	-3.0812475042	-2.2306737826
C	4.1722372899	-0.7180054482	-1.3353541029
H	2.6559349137	0.6635453954	-0.6856867187
C	4.4373867516	-1.9793191062	-1.8703135556
H	3.5829374328	-3.8175380033	-2.6050971708
H	4.9900590089	-0.0425535891	-1.1016525838
H	5.4628170121	-2.2920169419	-2.0442411039
C	-2.2740247902	0.8986875367	-0.7986975195
C	-1.9334786924	1.5037944908	-2.0127691123
C	-3.6195863509	0.6732106772	-0.4947396949
C	-2.9283780946	1.8714009463	-2.9145858147
H	-0.8881545222	1.6845491108	-2.2464764452
C	-4.6152697374	1.019747823	-1.4085029499
H	-3.8797832595	0.2181159935	0.4568591844
C	-4.2712294325	1.6226245349	-2.6182342124
H	-2.6584465525	2.3503903944	-3.8512540626
H	-5.6576042914	0.8266596191	-1.1722387022
H	-5.0453577022	1.902919638	-3.3266260917

TS2d-Cbetaanti-Re
E(RM062X) = -1228.93704161
Sum of electronic and thermal Free Energies = -1228.509270
Charge = 0 Multiplicity = 1

C	0.0905179734	-0.0742315826	0.0579495605
C	-0.0135099626	-0.0708035495	2.4571979931
C	0.9694702638	-0.1692130954	1.2900067493
H	-0.3397103369	0.9667574621	2.6088305625
C	0.3106731592	-0.737759473	3.6953535091
H	0.9013340665	-1.6508424001	3.6514990083
C	-0.1186966101	-0.2381491147	4.9566563602
H	-0.2355978305	-0.9681418267	5.7636924341
H	-0.9654281662	0.4554998792	4.9292000228
C	-1.1790233725	-0.4286281659	0.4424584713
O	-1.3211639734	-0.6460414874	1.7388964456
C	0.4826208137	0.4325852195	-1.2485061766
O	-0.3109286597	0.899384205	-2.0697992618
H	1.5033547431	-1.1284702512	1.3498374015
H	1.7134350504	0.6305680145	1.3557291424
C	3.1792956753	0.963563179	6.894228979
C	2.2660764657	0.0780684825	6.0081640954
H	4.1802786542	1.0300795756	6.4597021137
H	3.2690373861	0.5404706029	7.898734044
H	2.6989395966	-0.1152168566	5.0235595265
H	2.0303284333	-0.8780147406	6.4842472845
C	2.3636891657	2.8338157237	5.6657060217
H	2.0699687194	3.8844995915	5.736448503
H	3.2950919324	2.7753620516	5.0952775195
C	1.244528864	2.011651279	4.9769561023
H	0.3017939955	2.5638604328	4.9192411563
H	1.5272977014	1.6819649922	3.9732751777
C	0.414091494	1.1892381908	7.0940775251
H	-0.5888278475	1.5865667528	6.9145779657
H	0.3338746476	0.2800874081	7.6966382653
C	1.3516539978	2.2409267283	7.7444812451
H	0.8898346383	3.2322564131	7.7260553752
H	1.5543070793	1.9757201796	8.7854052557
N	0.9868761409	0.7945464378	5.7832314255
N	2.6235120174	2.3167921291	7.0150597344
C	-2.3916437996	-0.6548017118	-0.3749264432
C	-2.3223050905	-1.3142053264	-1.6075692219
C	-3.6352504377	-0.247532969	0.1208906297
C	-3.4815823906	-1.5470348646	-2.3412103028
H	-1.3603545329	-1.6486574653	-1.9848885441
C	-4.7935433632	-0.4677058392	-0.6229311012
H	-3.6894336697	0.250628513	1.084450986
C	-4.7190250087	-1.1186745828	-1.8540502976
H	-3.4216878735	-2.0667783403	-3.292916776
H	-5.7532441165	-0.1354515845	-0.2383987665
H	-5.6219294576	-1.299478667	-2.4300775147
C	1.9490700248	0.432003338	-1.59335723
C	2.4627602154	1.4879287564	-2.3538596811
C	2.7907748353	-0.6224630533	-1.2241650779
C	3.8062693896	1.501957955	-2.7211843822
H	1.8014634774	2.2980724391	-2.6474758773
C	4.1303521795	-0.6202467847	-1.6117425483
H	2.3964973206	-1.4598993103	-0.6554940617
C	4.6417986689	0.4456118508	-2.3522224989
H	4.2018181567	2.3327638403	-3.298277618
H	4.7730141329	-1.4510077334	-1.3357801918
H	5.6878311226	0.4521464434	-2.6446361716

TS2d-Cbetasyn-Si
E(RM062X) = -1228.93953022
Sum of electronic and thermal Free Energies = -1228.510278
Charge = 0 Multiplicity = 1

C	0.1043313366	0.0197983639	0.0200591052
C	-0.0193883654	0.0935669089	2.4146149584
C	0.9776734334	0.151626891	1.2535373504
H	-0.3282876035	-0.9430260672	2.6132998467
C	0.3306345089	0.789592784	3.6360936606
H	0.8546206531	1.7401030993	3.5469450747
C	-0.1407196749	0.3722387617	4.9109663415
C	-1.1647287431	0.3799572767	0.387967181
O	-1.3064767532	0.6350556899	1.6828315867
C	0.5039423148	-0.5166352882	-1.275152738
O	-0.2841512963	-1.0330611039	-2.0700739443
H	1.7183709282	-0.6472132689	1.3485327387
H	1.5132736584	1.1111677058	1.2849490222
C	-3.020135078	3.0092210005	5.8451336023
C	-1.6034399455	2.4691073104	5.5258883578
H	-3.4659023724	3.4748428849	4.9613730489
H	-2.9687422827	3.7588717318	6.6393826347
H	-1.2276065773	2.8244465956	4.562716185
H	-0.8794406274	2.7237684053	6.3057695689
C	-4.1060151775	1.0097615136	5.1356848942
H	-4.65811745	0.1357015257	5.4931961385
H	-4.71635291	1.5168640115	4.383222826
C	-2.7450170721	0.5886618842	4.5250224318
H	-2.67368623	-0.4942064172	4.3820525626
H	-2.5406086534	1.0768486633	3.5706765541
C	-1.9671220701	0.4548197589	6.8090845684
H	-2.114693194	-0.6236709929	6.701972911
H	-1.0945543731	0.6324724779	7.4440049642
C	-3.2342777963	1.1687829161	7.3490905935
H	-3.9342023732	0.4374404542	7.761722161
H	-2.9696481146	1.8758632701	8.1405615239
N	-1.6650656358	0.9903373206	5.4586754845
N	-3.900342695	1.9148180205	6.2739496731
H	-0.3907252028	-0.6914566299	4.9936553024
H	0.443015987	0.7147378637	5.7705128563
C	-2.3827979464	0.5836069353	-0.4249660002
C	-2.3219244411	1.1813679213	-1.6890357537
C	-3.6252179781	0.2229717102	0.1093176422
C	-3.4886548618	1.3953017061	-2.4167158757
H	-1.361346312	1.4844399974	-2.0952625754
C	-4.7911104694	0.4252674419	-0.6274769576
H	-3.6723246925	-0.2241571668	1.0983639628
C	-4.7248735748	1.011827739	-1.8909550685
H	-3.4349112818	1.8661346317	-3.3938659716
H	-5.7501764232	0.1295221437	-0.2125585265
H	-5.6336250514	1.1779303065	-2.4621756508
C	1.9645777596	-0.4759432503	-1.6364355059
C	2.7752529582	0.6103394147	-1.2905701284
C	2.5029981831	-1.5244542329	-2.390245903
C	4.1091241802	0.6475775852	-1.6954203693
H	2.359573781	1.4416747541	-0.7282716944
C	3.8410379372	-1.4978776744	-2.7756364501
H	1.8656276476	-2.3599392129	-2.6648446815
C	4.6456118974	-0.4098238397	-2.4302976079
H	4.7276382922	1.5019487149	-1.4370955515
H	4.2572160904	-2.3219927804	-3.3477236302
H	5.6873999132	-0.3857388483	-2.7365958484

TS2d-Cbetasyn-Re
E(RM062X) = -1228.93985228
Sum of electronic and thermal Free Energies = -1228.509831
Charge = 0 Multiplicity = 1

C	0.1542849806	-0.0654112612	0.1187788611
C	0.1508263011	-0.1012198384	2.5157382935
C	1.0916746206	-0.1446089254	1.3094693465
H	-0.1855320383	0.9279390845	2.7102255375
C	0.5680868273	-0.7659854012	3.7331515542
H	1.0447885548	-1.7408191335	3.6313119744
C	0.083858266	-0.3705436495	5.0118500306
H	-0.1320590185	0.697128368	5.1301536371
H	0.6466373242	-0.7599266747	5.8648723977
C	-1.0795929139	-0.4729712239	0.5530617584
O	-1.1457641565	-0.7110167146	1.8583790103
C	0.4666144614	0.483054889	-1.1951842752
O	-0.3828400518	0.9377554748	-1.9640895059
H	1.661927677	-1.0844052288	1.3281483054
H	1.806704924	0.6814364293	1.3545899858
C	-3.9042774316	-0.8720330532	5.123042865
C	-2.5426416938	-0.2091612504	4.7946190067
H	-4.2738865334	-1.4427668804	4.2654420795
H	-4.6463117708	-0.1085997942	5.3719300064
H	-2.3124227861	-0.2444986478	3.7272636247
H	-2.4978161201	0.8280961842	5.1391640421
C	-2.9116024337	-2.9159563058	5.8402091283
H	-2.701582123	-3.5283807357	6.7218317715
H	-3.4595048296	-3.5288619469	5.1193554744
C	-1.5952921828	-2.3949372871	5.2096587692
H	-0.7134684425	-2.896336653	5.6192924559
H	-1.5802975649	-2.4938414967	4.1215238977
C	-1.6347882305	-0.7384804247	6.9693479165
H	-0.9135639627	-1.389448426	7.4715308162
H	-1.3825976458	0.303577072	7.1869875417
C	-3.0961936375	-1.0834213583	7.3569847479
H	-3.1105680305	-1.713013531	8.2505255307
H	-3.6658562514	-0.1742126882	7.5695482056
N	-1.472460014	-0.9492235282	5.5096521571
N	-3.7625663922	-1.7938430351	6.2574406083
C	-2.3259687886	-0.7443136463	-0.1941756046
C	-3.556626221	-0.4467788743	0.4028887179
C	-2.2995884995	-1.3487037785	-1.4562127442
C	-4.7471254932	-0.7192445589	-0.2691907912
H	-3.5763175141	0.0059071036	1.3902806859
C	-3.4896780145	-1.633128287	-2.1189595991
H	-1.3469041397	-1.6016361213	-1.9119487514
C	-4.7155903724	-1.3130448602	-1.5305056852
H	-5.697565606	-0.4740711798	0.1955158281
H	-3.4620614278	-2.1093198376	-3.0945980686
H	-5.6423815223	-1.5334652775	-2.0521556124
C	1.9127261839	0.5444866246	-1.6092110348
C	2.8114090706	-0.4814102912	-1.2994888356
C	2.3462565547	1.6325815928	-2.3745957031
C	4.1285256819	-0.4197041072	-1.7534844211
H	2.4791090838	-1.341461314	-0.7251355671
C	3.6679164788	1.7061762594	-2.8072973455
H	1.639017761	2.4196476022	-2.6201788771
C	4.5605581803	0.677468817	-2.4991620682
H	4.8169908843	-1.227690259	-1.5242139396
H	4.0009860444	2.5615445571	-3.3877825404
H	5.5897330131	0.7295243165	-2.8423229875

TS2d-Cbetaanti-Si
E(RM062X) = -1228.93708855
Sum of electronic and thermal Free Energies = -1228.507150
Charge = 0 Multiplicity = 1

C	0.078360936	-0.0052369987	0.0246145253
C	0.0152289276	0.0697550053	2.423493112
C	0.9805291157	0.1071659582	1.2383407976
H	-0.33653124	-0.9536185031	2.6099860403
C	0.3766227388	0.763272956	3.6363080162
H	0.9938621106	1.6560562042	3.5570547188
C	-1.1750405044	0.3945177827	0.4177804066
O	-1.288429437	0.6586771137	1.7082608738
C	0.4369009899	-0.5653441791	-1.2695826794
O	-0.3797761808	-1.0390178774	-2.0637226945
H	1.7059403652	-0.7082860573	1.3170039846
H	1.538701913	1.0543428973	1.257967663
C	1.362947385	-2.1118147891	7.7761729516
C	0.4510067388	-1.0592579152	7.0918197197
H	0.8645281803	-3.0844158427	7.8169244869
H	1.596476144	-1.8033774165	8.7985710859
H	-0.5635544317	-1.4351520471	6.9326428638
H	0.3980589068	-0.1273896112	7.6615494142
C	2.3108942398	-2.8297744386	5.7062848265
H	3.2354411278	-2.8459476195	5.1224287363
H	1.9629706066	-3.8592171716	5.8263006813
C	1.2268385913	-1.9796314	4.9949535076
H	1.5198679993	-1.6931174869	3.9811405807
H	0.2607895685	-2.4914428146	4.9551358937
C	2.3306664946	-0.0531816851	5.9529148448
H	2.7546171065	0.1028339835	4.9580281904
H	2.1357463534	0.9212728318	6.4097564608
C	3.2275153	-0.949396868	6.8456197616
H	4.2123280338	-1.0760345627	6.388297665
H	3.3632391637	-0.4964987838	7.8316306808
N	1.0240501769	-0.728533927	5.7637628436
N	2.6164576255	-2.271272087	7.0291343743
C	-0.0536406417	0.3172049318	4.9173019572
H	-0.134468708	1.0754860219	5.7024440217
H	-0.9251711714	-0.3455842998	4.9221095542
C	-2.3964351211	0.6232285269	-0.3857634544
C	-2.3333937573	1.237203209	-1.6419336248
C	-3.6404786417	0.2659740032	0.1459043424
C	-3.4997611557	1.4735847569	-2.3632052268
H	-1.3705914057	1.5346645122	-2.0471611669
C	-4.8063769006	0.4893650194	-0.5850537365
H	-3.6897663629	-0.1959882563	1.1275545874
C	-4.7382250001	1.0942127926	-1.8397534964
H	-3.44390209	1.9577448513	-3.3337379304
H	-5.7669624194	0.1956470138	-0.1721587937
H	-5.6467693764	1.2772204224	-2.4061313728
C	1.897742428	-0.6134399229	-1.634454036
C	2.7682607213	0.4358519453	-1.321879384
C	2.3761405886	-1.7128734881	-2.3551039938
C	4.1013743072	0.3857998502	-1.728083787
H	2.40112832	1.3047962841	-0.7829711268
C	3.7134439042	-1.7744826155	-2.7396000835
H	1.6920139825	-2.5190770613	-2.603994549
C	4.5777005095	-0.7226367179	-2.4285931992
H	4.7673010964	1.2119131928	-1.4966751111
H	4.0812025713	-2.6387305113	-3.2851004109
H	5.6189349469	-0.7654538142	-2.7347241524

2d
E(RM062X) = -883.756894313
Sum of electronic and thermal Free Energies = -883.505055
Charge = 0 Multiplicity = 1

C	-0.1683625134	0.6139146242	0.1670390124
C	-0.2078273955	0.3280762764	2.5006738029
C	0.7667010086	0.6808421507	1.3630967124
H	-0.668337739	1.247947998	2.8871738439
C	0.3905823948	-0.4536867708	3.6245353419
H	1.1513696313	0.0875831541	4.1856544574
C	0.0697031819	-1.7048643176	3.9494010037
H	0.5617894268	-2.2070516684	4.7768646474
H	-0.6896443116	-2.2566641005	3.4028660004
C	0.0373752822	1.2871360579	-1.1226260128
O	-0.8938022252	1.6790333957	-1.819639375
C	-1.2698220265	-0.0831134219	0.5445184232
O	-1.2673231958	-0.4220266797	1.8563801297
H	1.5745036381	-0.0580175415	1.2998994393
H	1.2131033156	1.6667253375	1.5116000658
C	-2.4272497265	-0.5860071726	-0.2126960318
C	-2.2796135073	-1.047214809	-1.5265411416
C	-3.6826893353	-0.64021971	0.4048926341
C	-3.3814044921	-1.5369090143	-2.2203566723
H	-1.301292834	-1.0309503508	-1.9970801126
C	-4.7856984672	-1.1235559261	-0.2967329327
H	-3.7929054526	-0.2940765374	1.4279326311
C	-4.6372741077	-1.5709660037	-1.609213704
H	-3.2605736465	-1.8988076553	-3.2367926326
H	-5.7589543259	-1.1536994376	0.1833893445
H	-5.4958164511	-1.9520698682	-2.1540442537
C	1.448184228	1.544339029	-1.5656879445
C	2.4670972739	0.6156984025	-1.3291849238
C	1.7283052017	2.7146452999	-2.2800340434
C	3.7554751215	0.8561318432	-1.805027158
H	2.2503673862	-0.3080547587	-0.8002526457
C	3.0203211536	2.9643443148	-2.7347084253
H	0.9286180459	3.4253187641	-2.4671766699
C	4.034821045	2.0337251051	-2.4989680262
H	4.539542345	0.1248419019	-1.6339000202
H	3.2372656854	3.8807335568	-3.2752293949
H	5.0413040804	2.2254350953	-2.8589922478

int1d-zw
E(RM062X) = -883.689569895
Sum of electronic and thermal Free Energies = -883.440686
Charge = 0 Multiplicity = 1

C	0.2060878357	0.2779492298	-0.0690963437
C	0.0513978106	0.4039413706	1.4334689537
C	0.892779178	-1.0426181021	3.3509565767
H	-0.1119513617	-1.1506902914	3.75011033
C	1.9525318224	-1.6893514977	3.9018312806
H	1.8417205853	-2.3139828183	4.7839351223
H	2.9471630962	-1.5901131969	3.4729335148
C	-0.0890075113	1.4710903074	-0.8039911564
O	-0.5389914738	2.4953682619	-0.2494379064
C	0.4926686794	-0.9994214055	-0.6437128739
O	0.5524747559	-1.290713578	-1.8524519021
H	0.2182206658	1.4739533239	1.6685068009
H	-0.9422294371	0.1560789628	1.8259072381
C	0.1704522582	1.5731203538	-2.2856308763
C	-0.8397940899	2.0536471135	-3.1218517521
C	1.4367208906	1.3142580091	-2.8180129206
C	-0.5981071773	2.246626946	-4.4829792633
H	-1.8182608148	2.2727266697	-2.7029857499
C	1.6890004552	1.5351932381	-4.1699864739
H	2.2230663229	0.9370152546	-2.1696627448
C	0.6687998133	1.9926680555	-5.0083221441
H	-1.3948019069	2.6039672903	-5.1291898577
H	2.6793736909	1.3445236881	-4.5736467609
H	0.863421423	2.1538331365	-6.0647329605
C	0.7272183021	-2.1412567492	0.3332299686
C	-0.3483821659	-2.7346056228	1.0130233412
C	2.0194194051	-2.652672873	0.5095954438
C	-0.1266709545	-3.7938088951	1.8877644724
H	-1.3543091962	-2.3481134727	0.8652757224
C	2.2439157537	-3.6985752866	1.400485941
H	2.8494623182	-2.2056349589	-0.0322723084
C	1.1726349959	-4.2629462637	2.1007104389
H	-0.9611176926	-4.2431453217	2.4179683967
H	3.2514144077	-4.0736774656	1.5547533787
H	1.3488001648	-5.0773674691	2.7973036735
C	1.0960811318	-0.2920323261	2.177466419
H	2.1227734514	-0.167734116	1.8293906811

TS2e-CbetaMe-anti
E(RM062X) = -1037.26492121
Sum of electronic and thermal Free Energies = -1036.883899
Charge = 0 Multiplicity = 1

C	0.1194714538	-0.0426893073	0.0581957239
C	0.0165364819	-0.0475217555	2.4630364654
C	0.9993782	-0.0922027681	1.2905902743
H	-0.3294011456	0.9771156224	2.6504254601
C	0.3586173461	-0.7495272369	3.6762633044
H	0.9610484943	-1.6525184018	3.5970510896
C	-0.0729370846	-0.3041705447	4.9584198384
H	-0.1797959159	-1.0674813986	5.7356918339
H	-0.9306305477	0.3766729408	4.9599842678
C	-1.1485479848	-0.4168016139	0.4413340403
O	-1.2939796396	-0.6188530196	1.7336152268
C	0.4731073363	0.3345900885	-1.2905528912
O	-0.362080352	0.5644587727	-2.1768896278
C	-2.3585043256	-0.6431916315	-0.4011176558
H	-3.0111528433	-1.3707785785	0.0871371481
H	-2.9121245309	0.2966267106	-0.5073767409
H	-2.0853567729	-0.9783122817	-1.4016838699
H	1.5812413628	-1.0226420329	1.3507911983
H	1.7041353363	0.7423653487	1.3621173049
C	3.2237209052	0.8614686732	6.9104321921
C	2.3104502904	-0.0032191674	6.0038358455
H	4.2139677103	0.96797563	6.4597505493
H	3.3428865988	0.3958806213	7.8927023047
H	2.7340538574	-0.1572302318	5.0085793125
H	2.0904606985	-0.9781560394	6.4481647988
C	2.3588706295	2.7732859086	5.7837424556
H	2.0395349361	3.8111641765	5.9105722695
H	3.2854585985	2.7680522765	5.2029160604
C	1.2541060195	1.9592054839	5.0627671222
H	0.301147999	2.4953817866	5.029859181
H	1.5393754954	1.6770244751	4.0455686357
C	0.4508770935	1.0353733139	7.1476396175
H	-0.5549119691	1.4344343345	6.989780171
H	0.3769202953	0.0993881022	7.7082498357
C	1.3841247106	2.0614533235	7.8432602536
H	0.9087848275	3.0453633521	7.8885910603
H	1.6051971616	1.7401677798	8.864481859
N	1.0200863753	0.7047955014	5.817443977
N	2.6444850742	2.1963022613	7.1030454583
C	1.9309586766	0.4997834132	-1.6366148395
C	2.3016192831	1.5558377439	-2.476786645
C	2.9083511198	-0.3954580255	-1.189623089
C	3.6336073396	1.7304214801	-2.8447287661
H	1.5367921243	2.2395044078	-2.8340855128
C	4.238640755	-0.2348900606	-1.5769294747
H	2.6298568348	-1.2365614708	-0.5618098395
C	4.6049778162	0.833280968	-2.3961758022
H	3.9146715368	2.5629060215	-3.4831653066
H	4.9880256694	-0.9441261827	-1.2377983563
H	5.6430842826	0.9643517305	-2.6877138426

TS2e-CbetaMe-syn
E(RM062X) = -1037.26712537
Sum of electronic and thermal Free Energies = -1036.885640
Charge = 0 Multiplicity = 1

C	0.1548898398	-0.0683904175	0.091473566
C	0.079733513	-0.1174142447	2.4958775813
C	1.0518742527	-0.1338016271	1.3107093327
H	-0.2442126187	0.9070688405	2.7268152992
C	0.4719721227	-0.8368254713	3.688980397
H	0.9929862133	-1.7844907586	3.5601494146
C	0.0570051475	-0.4468644022	4.994078422
H	-0.167288237	0.6188084682	5.1173205552
H	0.6776577308	-0.8192293825	5.8146230659
C	-1.1053606551	-0.444868309	0.4871845942
O	-1.2287810474	-0.6618614593	1.7838003946
C	0.4849722319	0.3315771172	-1.2586478433
O	-0.3677059641	0.5603947931	-2.1272892001
C	-2.3326966211	-0.6609214895	-0.3312547264
H	-2.9776700621	-1.3902847188	0.164547446
H	-2.8851670872	0.2813544309	-0.4201131673
H	-2.0805310941	-0.9897056456	-1.33937631
H	1.6396706254	-1.0616674884	1.3456854372
H	1.7519394546	0.7032936107	1.3877926255
C	-3.8974347683	-0.9825581837	5.4042569911
C	-2.5595593761	-0.5685263649	4.7405420113
H	-4.5618150321	-1.4373909566	4.6645858355
H	-4.4043172059	-0.1118068756	5.830108212
H	-2.4218588075	-1.0105779221	3.7521192854
H	-2.4624998737	0.5177551279	4.6518413928
C	-2.8390103134	-3.0442838716	5.9630739078
H	-2.7636976384	-3.8270579742	6.7224876593
H	-3.3489853725	-3.4611494108	5.0897716468
C	-1.4268317331	-2.5318597034	5.5816540042
H	-0.6648225925	-2.8484998722	6.3002698021
H	-1.1226259992	-2.8481007604	4.5803516791
C	-1.650418936	-0.5735149896	6.9800729559
H	-0.7508283281	-0.8130940046	7.5535229517
H	-1.7637079237	0.5132982366	6.9336496321
C	-2.910913945	-1.2697110704	7.5561141788
H	-2.6294638453	-2.0203100834	8.3002904439
H	-3.5621604108	-0.5352958509	8.0375002903
N	-1.4427878236	-1.0501324891	5.5901069373
N	-3.6587545191	-1.9443576339	6.4880087478
C	1.9337102697	0.5256249691	-1.6245352424
C	2.935133638	-0.3543346474	-1.2016806466
C	2.2696665293	1.5973441718	-2.4596776803
C	4.2553598206	-0.1642018558	-1.6097239812
H	2.6832292704	-1.2058362677	-0.5767064583
C	3.591601835	1.8015876058	-2.8474206775
H	1.4856590287	2.2695214431	-2.7966053891
C	4.5870693224	0.9187103892	-2.4244394357
H	5.0241286128	-0.8616690259	-1.2902123465
H	3.8464806347	2.6457616113	-3.4814568285
H	5.6174667386	1.0727501522	-2.7316816668

TS2e-CbetaPh-anti
E(RM062X) = -1037.26238262
Sum of electronic and thermal Free Energies = -1036.880538
Charge = 0 Multiplicity = 1

C	0.0299067845	0.0344493202	-0.0702000143
C	0.0096255037	0.0293474431	2.3483179554
C	0.9441797558	0.0455070399	1.1372708794
H	-0.3478209404	1.0411742539	2.5788139622
C	0.41847663	-0.696170261	3.5267190005
H	1.0409601986	-1.5798026249	3.3997726352
C	-0.0095580207	-0.317082301	4.835133078
H	-0.0795463021	-1.1177627137	5.5788208473
H	-0.8993207579	0.320592322	4.8717411034
C	-1.2150496079	-0.3531694319	0.3585975458
O	-1.316865279	-0.5425487088	1.6644489194
C	0.5076202516	0.1686934652	-1.4395033182
O	-0.2084983807	0.115552948	-2.4416620419
C	1.9988822332	0.3866035061	-1.591553372
H	2.5616710266	-0.4033369272	-1.0825352111
H	2.2613805782	0.400887015	-2.650373094
H	2.2902199058	1.3374125206	-1.1309075225
H	1.591985065	-0.8440310897	1.1525359382
H	1.5975016704	0.9231176627	1.1838101276
C	3.2370317855	0.8956882588	6.8145896491
C	2.3566266742	0.0257684661	5.8810649903
H	4.2258551192	1.0431000203	6.3724791221
H	3.3645181144	0.409613426	7.7857724557
H	2.7877294459	-0.0869566641	4.8836074279
H	2.1688215016	-0.9676125248	6.2981269185
C	2.3148072811	2.8050054353	5.7310072181
H	1.9652413019	3.8300603729	5.8799301006
H	3.2420839793	2.8389797034	5.1522674471
C	1.2352388148	1.9769985375	4.9891394109
H	0.2652711678	2.4825826297	4.9727275851
H	1.5263304105	1.7311835085	3.9643072209
C	0.4625519385	0.9716484263	7.0489405719
H	-0.5568304399	1.3382712146	6.9005556603
H	0.4219296603	0.0199236988	7.585727637
C	1.3580884228	2.0130077356	7.7694551412
H	0.84923017	2.9788529888	7.8335014016
H	1.5860342301	1.6772592854	8.7844547078
N	1.0418910553	0.6942316399	5.7096089613
N	2.6155348422	2.2065744503	7.0373418982
C	-2.4571966353	-0.6338396067	-0.4004266616
C	-3.2042319058	-1.7722937736	-0.0709967617
C	-2.9341466254	0.2422391232	-1.3806363058
C	-4.3965840362	-2.0475513457	-0.7371807899
H	-2.8425437637	-2.4435689077	0.7024372427
C	-4.1376503862	-0.0241046174	-2.0302012301
H	-2.3627639771	1.1290786839	-1.6302551447
C	-4.866770074	-1.1724403621	-1.717046679
H	-4.9612761141	-2.9405756663	-0.4862715764
H	-4.5072232658	0.6663909625	-2.782640637
H	-5.8005182889	-1.3811616323	-2.23122406

TS2e-CbetaPh-syn
E(RM062X) = -1037.26565609
Sum of electronic and thermal Free Energies = -1036.885494
Charge = 0 Multiplicity = 1

C	0.0435122607	-0.0828891577	-0.0543198522
C	0.0376073297	-0.106427488	2.3544935391
C	0.9710039229	-0.1496840188	1.1405595005
H	-0.2578364547	0.9280109559	2.5811655515
C	0.4520165127	-0.8154550141	3.5460984918
H	0.9594613527	-1.7705787171	3.4176855473
C	0.0593940229	-0.406604302	4.8557495996
H	-0.157709313	0.6628973376	4.9606952314
H	0.7020823933	-0.7584040038	5.6688718678
C	-1.2063942172	-0.4366499837	0.3796347416
O	-1.2971875811	-0.6318865232	1.6898164449
C	0.4576700998	0.3012419244	-1.3975860848
O	-0.3197068889	0.4983960768	-2.3331059051
C	1.9455578883	0.4983210531	-1.5964211474
H	2.5136610006	-0.3485688602	-1.197706936
H	2.160361272	0.625575949	-2.658475941
H	2.2743465658	1.3930020274	-1.055010404
H	1.5554603761	-1.0823023621	1.1483727711
H	1.6793435941	0.6822684289	1.1888049844
C	-3.8716783892	-0.9739773999	5.3396443137
C	-2.551385767	-0.5702699809	4.6356112853
H	-4.540430768	-1.4706481774	4.6314371407
H	-4.3844227572	-0.0925754071	5.7354737262
H	-2.4223990711	-1.0512824077	3.6645147133
H	-2.4713464046	0.5125647034	4.4986006098
C	-2.7746908702	-2.993844518	5.9690440153
H	-2.6769648901	-3.7433352935	6.758827788
H	-3.2917708044	-3.4536985061	5.1220282129
C	-1.3758587368	-2.4796806544	5.5447765799
H	-0.5996734851	-2.7516247146	6.2663407076
H	-1.0803781539	-2.835895994	4.5544749206
C	-1.6073300099	-0.4642878097	6.8597217391
H	-0.694555427	-0.6649333995	7.4271716621
H	-1.7387931499	0.6175321519	6.7675185609
C	-2.8466381907	-1.1537634591	7.4859942016
H	-2.5414556014	-1.8681521601	8.2559202796
H	-3.5008433065	-0.4091362691	7.9470710107
N	-1.4131131941	-0.9979460587	5.4875922712
N	-3.6015030777	-1.8838280498	6.4600495933
C	-2.4644517292	-0.6736932405	-0.3639214174
C	-3.6729841491	-0.2323889332	0.1887392336
C	-2.4757856242	-1.3846599953	-1.5687980539
C	-4.8761982325	-0.4708345496	-0.4733734653
H	-3.6636973185	0.3053607904	1.1325828116
C	-3.680532421	-1.6384812128	-2.2186905247
H	-1.5408232762	-1.7385133045	-1.9905101853
C	-4.8819414595	-1.1758848601	-1.6770125988
H	-5.808088986	-0.1112988797	-0.0471600675
H	-3.6834237335	-2.1998627188	-3.1482722786
H	-5.8197468806	-1.3694251987	-2.1897135127

TS2e-C2Ph
E(RM062X) = -1037.25165379
Sum of electronic and thermal Free Energies = -1036.872222
Charge = 0 Multiplicity = 1

C	0.0477407474	0.0124194645	-0.0426972583
C	-0.0086105226	-0.0104430133	2.4354875653
C	0.8881501733	0.0622076541	1.2233604838
H	-0.1253281667	0.8249379031	3.1132703109
C	-0.7777239627	-1.2447935001	2.672692467
H	-0.4885374522	-2.1106533934	2.0778439406
C	-1.7754065164	-1.3306244285	3.554831422
H	-2.3174892078	-2.2604612376	3.7023608133
H	-2.0892004824	-0.4681035722	4.1388031296
C	-1.2053323396	0.5789679487	0.1807870881
O	-1.4993111151	0.9153746913	1.3810967285
C	0.5368187476	-0.5767606636	-1.2658848973
O	-0.1346430435	-0.7289694423	-2.2972838393
C	1.9752984265	-1.0734150171	-1.262388314
H	2.0618183977	-1.973730408	-0.6419790805
H	2.2643737769	-1.32463778	-2.2843392063
H	2.6675726101	-0.3287164228	-0.8566870872
H	1.5726553506	-0.7896556136	1.257145806
H	1.4906251012	0.9775457188	1.2728576182
C	3.7139420854	0.1953659545	4.7972328465
C	2.718945762	0.3496227872	3.6125334453
H	3.6780934963	1.0691512932	5.4551165236
H	4.7367444162	0.0906886599	4.4234854856
H	2.3501050454	1.3767638834	3.5246636522
H	3.1881176735	0.0640551544	2.6671740122
C	2.0717834743	-0.7525550781	6.2379926959
H	1.7548808072	-1.6856173003	6.7142455401
H	2.1925782435	0.0055220329	7.0172325213
C	1.0287095873	-0.2887385385	5.1839574912
H	0.0850110425	-0.8306540657	5.2939834047
H	0.827000216	0.7844605341	5.26346738
C	2.0106119631	-1.9453327414	3.7536364495
H	1.1734757233	-2.5780091824	4.0623265283
H	2.2582042426	-2.1793982508	2.7131380336
C	3.2387184286	-2.1374913075	4.6856945023
H	3.1287493566	-3.0506008799	5.2782133817
H	4.1628302803	-2.2156007555	4.1045639775
N	1.5621027134	-0.5426169449	3.833212517
N	3.3704936521	-0.9892456794	5.5934364141
C	-2.2528059076	0.8529332929	-0.850129055
C	-3.5671253806	0.4482577318	-0.6001824239
C	-1.9641297957	1.5750443247	-2.0117123279
C	-4.5790188371	0.7353739638	-1.5166786828
H	-3.791704144	-0.0988166601	0.3115684052
C	-2.9784606153	1.8844363736	-2.9144390088
H	-0.9427253297	1.8888781186	-2.2060545908
C	-4.2868631352	1.4580347259	-2.6733436069
H	-5.5948023393	0.4024589211	-1.3234168965
H	-2.7485407521	2.4545484216	-3.8100172521
H	-5.0751512062	1.6908093214	-3.3835626731

TS2e-C2Me
E(RM062X) = -1037.25471608
Sum of electronic and thermal Free Energies = -1036.875740
Charge = 0 Multiplicity = 1

C	0.0671013661	0.0337166571	0.0288080915
C	-0.0049546336	0.0413564975	2.5012169859
C	0.9020198692	0.0833286779	1.2968463223
H	-0.0951902885	0.8836101337	3.1748917988
C	-0.8022030221	-1.1695594312	2.7560595009
H	-0.5448267074	-2.0470596753	2.1638469894
C	-1.7890493431	-1.2200405176	3.6531758512
H	-2.3554389439	-2.1325627679	3.8167138022
H	-2.0700952597	-0.3442283604	4.2339958656
C	-1.184798562	0.6301438669	0.2357882947
O	-1.4632129965	0.9969118781	1.4247158368
C	0.4788595935	-0.5654639167	-1.2024477559
O	-0.2641390468	-0.7344496094	-2.190100214
C	-2.2167843607	0.8633557698	-0.8327116047
H	-2.9443538072	1.5944875497	-0.4737370506
H	-1.7589712091	1.1942889782	-1.7662776997
H	-2.7343320533	-0.0771207692	-1.0505253455
H	1.5761467069	-0.7767759716	1.3384590095
H	1.5127972499	0.991930665	1.351275241
C	3.7461237747	0.1716692425	4.8543118228
C	2.7438664239	0.347154718	3.678291476
H	3.7391083292	1.0491653501	5.5081646369
H	4.7623332314	0.0383134347	4.4718558019
H	2.3952772533	1.3817382823	3.5944403242
H	3.1991865575	0.0533189715	2.7280893712
C	2.0910150445	-0.7300596771	6.3109351012
H	1.7590528194	-1.6517322726	6.7990048145
H	2.2334698917	0.0328299971	7.0818191025
C	1.0490617146	-0.2556741099	5.2598833774
H	0.0989802937	-0.7850838361	5.3766564994
H	0.8614525361	0.8203486948	5.3368283229
C	1.9913813187	-1.9323830902	3.8277750385
H	1.1415668873	-2.5484217708	4.1357413489
H	2.2352233632	-2.1707979944	2.7873140296
C	3.2157529636	-2.1504183943	4.759885243
H	3.08280944	-3.055851865	5.3594788524
H	4.1365223701	-2.2572765599	4.1779616014
N	1.5712919738	-0.5214949929	3.9078080726
N	3.3794259899	-0.9999155666	5.6591999869
C	1.9026748949	-1.0630945076	-1.3243222882
C	2.1265003619	-2.310682046	-1.9172670193
C	3.0004345992	-0.2961255381	-0.9185341131
C	3.4227334789	-2.7972202445	-2.0773993376
H	1.273783496	-2.8977722974	-2.2467136502
C	4.299760995	-0.7713267656	-1.0987548395
H	2.8442717517	0.6842733905	-0.4764255931
C	4.513253162	-2.0266815028	-1.6691474936
H	3.5830212473	-3.7743341092	-2.5241945418
H	5.1454055804	-0.1611885322	-0.7944892233
H	5.5243637964	-2.4015517523	-1.7990284288
